# High-throughput glycolytic inhibitor discovery targeting glioblastoma by graphite dots–assisted LDI mass spectrometry

**DOI:** 10.1126/sciadv.abl4923

**Published:** 2022-02-16

**Authors:** Rui Shi, Peichen Pan, Rui Lv, Chongqing Ma, Enhui Wu, Ruochen Guo, Zhihao Zhao, Hexing Song, Joe Zhou, Yang Liu, Guoqiang Xu, Tingjun Hou, Zhenhui Kang, Jian Liu

**Affiliations:** 1 Institute of Functional Nano and Soft Materials (FUNSOM), Jiangsu Key Laboratory for Carbon-Based Functional Materials and Devices, Soochow University, Suzhou, Jiangsu 215123, China.; 2 College of Pharmaceutical Sciences and State Key Lab of CAD&CG, Zhejiang University, Hangzhou, Zhejiang 310058, China.; 3 Department of Cancer Biology, Dana-Farber Cancer Institute, Boston, MA 02215, USA.; 4 Department of Biological Chemistry and Molecular Pharmacology, Harvard Medical School, Boston, MA 02115, USA.; 5 College of Information and Electrical Engineering, China Agricultural University, Beijing, China.; 6 Jiangsu Key Laboratory of Neuropsychiatric Diseases and College of Pharmaceutical Sciences, Soochow University, Suzhou, Jiangsu 215123, China.; 7 Macao Institute of Materials Science and Engineering, Macau University of Science and Technology, Taipa, Macau SAR 999078, China.

## Abstract

Malignant tumors will become vulnerable if their uncontrolled biosynthesis and energy consumption engaged in metabolic reprogramming can be cut off. Here, we report finding a glycolytic inhibitor targeting glioblastoma with graphite dots–assisted laser desorption/ionization mass spectrometry as an integrated drug screening and pharmacokinetic platform (GLMSD). We have performed high-throughput virtual screening to narrow an initial library of 240,000 compounds down to the docking of 40 compounds and identified five previously unknown chemical scaffolds as promising hexokinase-2 inhibitors. The best inhibitor (Compd 27) can regulate the reprogrammed metabolic pathway in U87 glioma cells (median inhibitory concentration ~ 11.3 μM) for tumor suppression. Highly effective therapy against glioblastoma has been demonstrated in both subcutaneous and orthotopic brain tumors by synergizing Compd 27 and temozolomide. Our glycolytic inhibitor discovery can inspire personalized medicine targeting reprogrammed metabolisms of malignant tumors. GLMSD enables large, high-quality data for next-generation artificial intelligence–aided drug development.

## Teaser

A high-throughput platform enables discovering HK2 inhibitors against glioma reprogrammed metabolisms and acquiring large PK data.

## INTRODUCTION

Reprogrammed metabolisms are critical in the energy supply and material resourcing of cancer cells during their survival, proliferation, and metastasis (*1*). Cancer cells tend to have higher glucose uptake to satisfy their abnormal metabolic needs by glycolysis (*2*, *3*). Hexokinase (HK), as a rate-limiting enzyme, catalyzes the conversion of glucose to glucose-6-phosphate in the glycolysis pathway. Among the subtypes of HK enzyme family, HK2 is overexpressed in malignant tumors, including glioblastoma, up to sevenfold higher than normal cells (*4*). The previous studies suggest that abnormal HK2 gene expression can be activated by p53 mutation, hypoxia-inducible factor 1 a (*5*), or other signaling molecules (e.g., insulin) (*6*). Consequently, it can assist tumor cells to harness the shortcuts of adenosine triphosphate (ATP) generation by aerobic glycolysis (notoriously as the Warburg effect) and change the metabolic profiles required by tumor proliferation and construction of tumor microenvironment (*7*). HK2 has been proposed as a pivotal target of the reprogrammed metabolisms for the development of anticancer drugs (*8*–*11*). Clinical trials have been initiated by using 2-deoxyglucose (2-DG) as a mimic of glucose to compete with the binding site of HK2 (*12*). Occasional case in hospitals has been reported to treat liver cancers with 3-bromopyruvate (3-BP) as a potent HK2 inhibitor (*13*). However, concerns on the side effects of these two drugs have been raised by clinical research, including uncontrolled inactivation, off-target interactions, promoted drug resistance, and other undesired physiochemical parameters (e.g., unable to cross the blood-brain barrier). There is an urgent need to screen new anticancer drug molecules to target HK2 with high inhibition efficiency and low side effect.

The widely used methods for high-throughput screening of drug molecules are based on fluorescent assays, colorimetric detection, and liquid chromatography–mass spectrometry (LC-MS) (*14*–*16*). However, the substrates for fluorescent assays must be molecularly labeled with aromatic groups, which might induce unexpected interactions with the targeting enzyme or uncontrollable molecular aggregation, thus generating bias in the screening (*17*). For instance, on the basis of the reports by independent groups, the compounds (e.g., resveratrol) screened out by the fluorescent assays exhibit diverse off-target activities and indirectly “activate” sirtuin (SIRT) family proteins to bind the artificially labeled peptide substrates (*18*). The colorimetric methods based on ultraviolet-visible (UV-Vis) absorption changes suffer from “blind zones” in screening when any candidate molecules exhibit overlapping UV-Vis absorption with the probes. Therefore, there is a serious risk of missing good drug candidates by using the colorimetric methods. Drug screening based on LC-MS has successfully been used to deliver hits for various challenging protein targets, including high-affinity binders of inhibiting Murine double minute 2-p53 (MDM2-p53) interaction or HIV capsid protein C-terminal domain dimerization (*19*), highly specific inhibitors to tropomyosin-related kinase A (*20*, *21*), and ginsenoside Rb2 as an activator for SIRT1 (*22*, *23*). However, the sample pretreatment steps in this technique typically require a long and tedious procedure (*24*, *25*) to remove the residual salts from the samples and calibrate the potential signal shifts very carefully. For instance, the core innovation of state-of-the-art ultrafast LC-MS has been proposed to cut the standard sample processing time (~5 min per sample, 8 hours per 96-well plate) yet only improve the throughput by fourfold (84 s per sample, 2.3 hours per 96-well plate) (*20*). To date, matrix-assisted laser desorption/ionization (MALDI) MS has little contribution to the field of drug screening, especially in the small-molecule drugs, because of the severe noise interference in the mass/charge ratio (*m*/*z*) 0 to 800 by the background fragmented organic matrices (*26*). It is the key element in pharmaceutics to develop sensitive, fast, and reliable screening methods for drug screening.

Here, we report a new chemical compound as an HK2 inhibitor for glioblastoma therapeutics, by using an integrated platform of high-throughput virtual screening (HTVS) and graphite dots (GDs)–assisted laser desorption/ionization MS for drug discovery (GLMSD). This integrated platform allows for rapid screening and successful identification of a panel (*n* = 5) of small molecular inhibitors targeting HK2 from initial 240,000 compounds. In comparison to the conventional methods for drug screening, GLMSD features the following merits: (i) label-free and no limitation of “blind-zones”; (ii) high-sensitivity, high-salinity tolerance leading to simplified sample pretreatment; (iii) improved throughput performance 12 times faster than the traditional LC-MS screening; (iv) minimal sample volume (≤10 μl per test), and the fewest sacrificed animals contributed by “one mouse for one complete pharmacokinetic profile”. As a brand-new HK2 inhibitor screened out by our platform, Compd 27 has been tested to treat glioblastoma in vitro and in vivo. Our experiments have revealed the mechanisms of Compd 27 in normalizing the reprogrammed glycolysis pathways of glioblastoma cells. We have demonstrated effective glioblastoma suppression by synergizing the HK2 inhibitor (Compd 27) and temozolomide (TMZ) in a subcutaneous tumor mouse model and an orthotopic mouse brain tumor model.

## RESULTS

### High-throughput screening of HK2 inhibitors by GLMSD

**Fig. 1.**
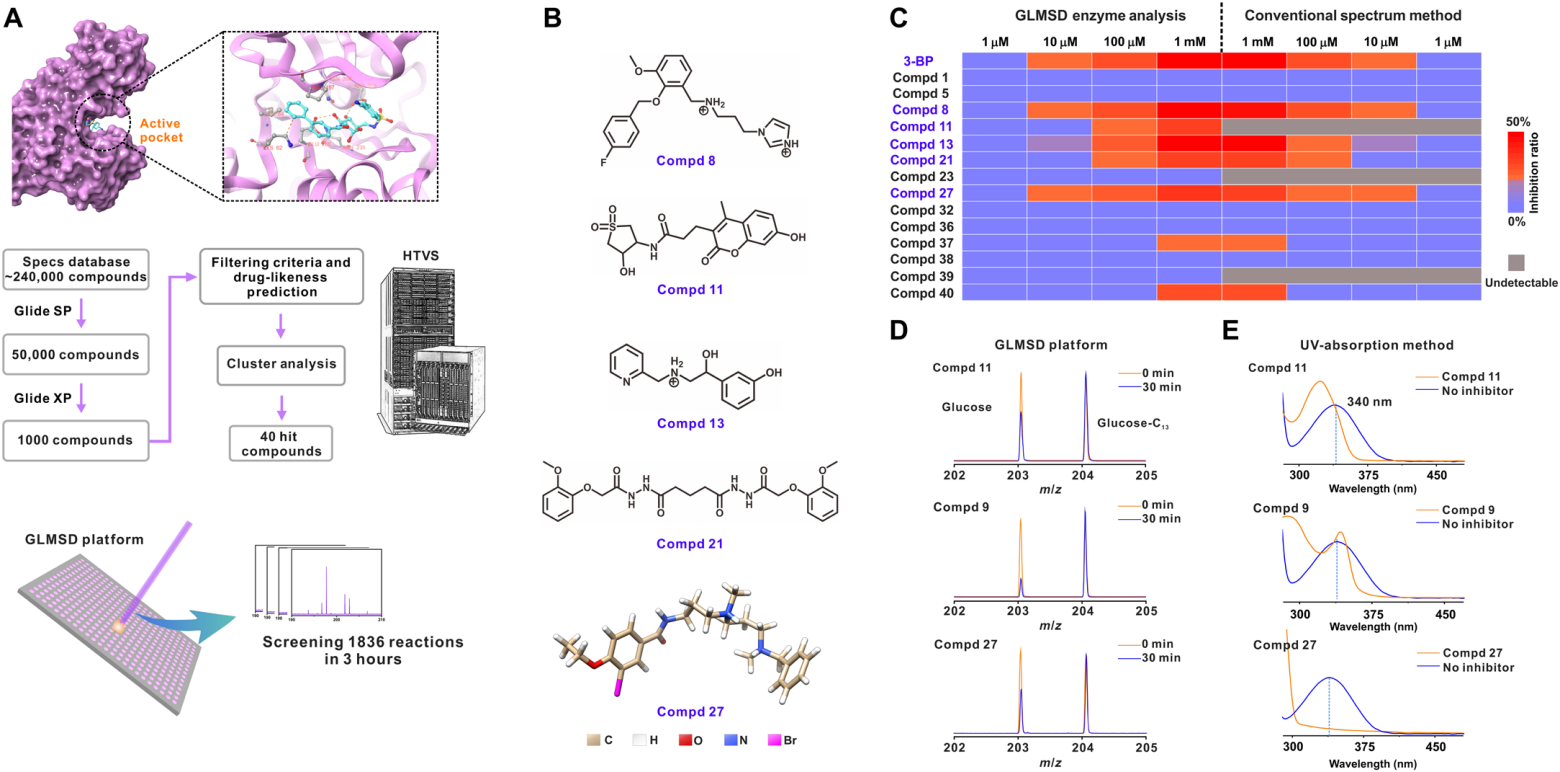
GLMSD platform for high-throughput screening of the HK2 inhibitors. (**A**) Schematic workflow of computer-aided simulations and GLMSD screening for HK2 inhibitor discovery. (**B**) Chemical structures of the five best new inhibitors (Compd 8, Compd 11, Compd 13, Compd 21, and Compd 27) obtained by GLMSD screen. (**C**) Heatmap of the inhibition efficiency of 15 soluble chemicals as HK2 inhibitor candidates at the gradient concentrations for the comparison between GLMSD and a conventional absorption-based screening method. Color codes: red for high inhibition efficiency; blue for low inhibition efficiency; and gray for the nondetectable effect (blind-zone). The complete datasets for comparison are available in the supporting information. (**D**) The mass spectra by GLMSD for Compd 11, Compd 9, and Compd 27. Color codes: orange (inhibition at 0 min); blue (inhibition at 30 min). Glucose at *m*/*z* 203.04; glucose-C_13_ (isotopic internal standard) at *m*/*z* 204.06. (**E**) The spectra using the conventional absorption-based screening method with a commercial HK assay kit. Color codes: orange (the UV absorption of candidate compounds); blue (the UV absorption of the probe from the commercial kit). Note: remarkable overlap between the orange and blue curves around 340 nm (the suggested wavelength for quantification by the protocol of the commercial kit) for Compd 11 or Compd 9.

An initial library of 240,000 chemical compounds was tested for docking to the protein structure of HK2 with an HTVS method (Fig. 1A). It was narrowed down to an intermediate library of 1000 top-ranked compounds by the docking simulations with the Glide module in Schrödinger, including the standard precision (SP) and extra precision (XP) modes sequentially. After being filtered with the drug-likeness prediction and clustering analysis, 40 chemical compounds with the lowest docking scores were selected into the final panel for the subsequent benchtop validation with the GLMSD platform.

We attempted to evaluate the performance of GLMSD in quantifying the biochemicals involved in the glycolytic reaction catalyzed by HK2 and demonstrated the following technical advantages: (i) an ultrahigh signal-to-noise ratio (10- to 340-fold better than α-cyano-4-hydroxycinnamic acid, 2-5-dihydroxybenzoic acid, or sinapinic acid; data S1); (ii) tolerance to the high salt contents required by the enzymatic reaction buffers (table S1); (iii) reproducible signal readouts (coefficient of variation lower than 6% in spot-to-spot evaluation; figs. S1 and S2); and (iv) accurate and precise quantification (relative error <7%, relative SD of <6%) with a linearity [coefficient of determination (*R*^2^) = 0.995] in the range of 10 nM to 10 μM using d-glucose-1-^13^C as the internal standard (fig. S3).

We tested the final panel of chemical compounds in gradient concentrations in the HK2- catalyzed reaction by quantifying the concentration changes of glucose using GLMSD. Our approach allowed for liquid sampling of only 1 μl of the mixture from each reaction for deposition on the MALDI plate and direct measurement of glucose without any requirement of sample purification. We demonstrated that GLMSD enabled high-throughput screening of the inhibitor candidates by collecting 1836 (= 102 × 6 × 3) mass spectra in 5.1 hours. In contrast, the standard LC-MS technique would take 61.2 hours to complete testing the same set of reactions. Figure 1B lists the chemical structures of Compds 8, 11, 13, 21, and 27, which exhibit the highest inhibition efficiency for HK2 detected by GLMSD screening. They represent brand-new chemical scaffolds for drug development, which have never been reported in the literature. Their structures feature a carbon-carbon chain containing the secondary/tertiary amines or amides and terminals with aryl groups or heterocyclic groups. Among them, Compd 27 was selected as the focus of this study because of its outstanding performance in terms of solubility and tumor cell suppression.

For the purpose of comparison, the conventional HK colorimetric assay was used to screen the same panel of inhibitor candidates. The results verified that the inhibition efficiency quantified by GLMSD was accurate and reliable (a summary heatmap in Fig. 1C and the representative raw spectra in Fig. 1, D and E). More detailed data of the comparison were available in figs. S4 and S5. Noteworthy, the colorimetric method suffered from the overlap of the UV absorption between the candidate compounds and the probe molecules near the wavelength of 340 nm, thus becoming inapplicable to identify several compounds in the conventional screening, including 10 compounds 3, 4, 6, 9, 11, 23, 25, 26, 28, and 39 (Fig. 1E and fig. S6). Therefore, 10 of 40 compounds were not able to be tested for HK2 inhibition by using the conventional colorimetric assay, missing a potentially good inhibitor candidate (Compd 11). In contrast, GLMSD was featured with probe/label-free detection, enabling a complete coverage of all the compounds in the tests (Fig. 1C and fig. S6).

### HK2 inhibitory potency on U87 cells and structure-activity relationship studies

**Fig. 2.**
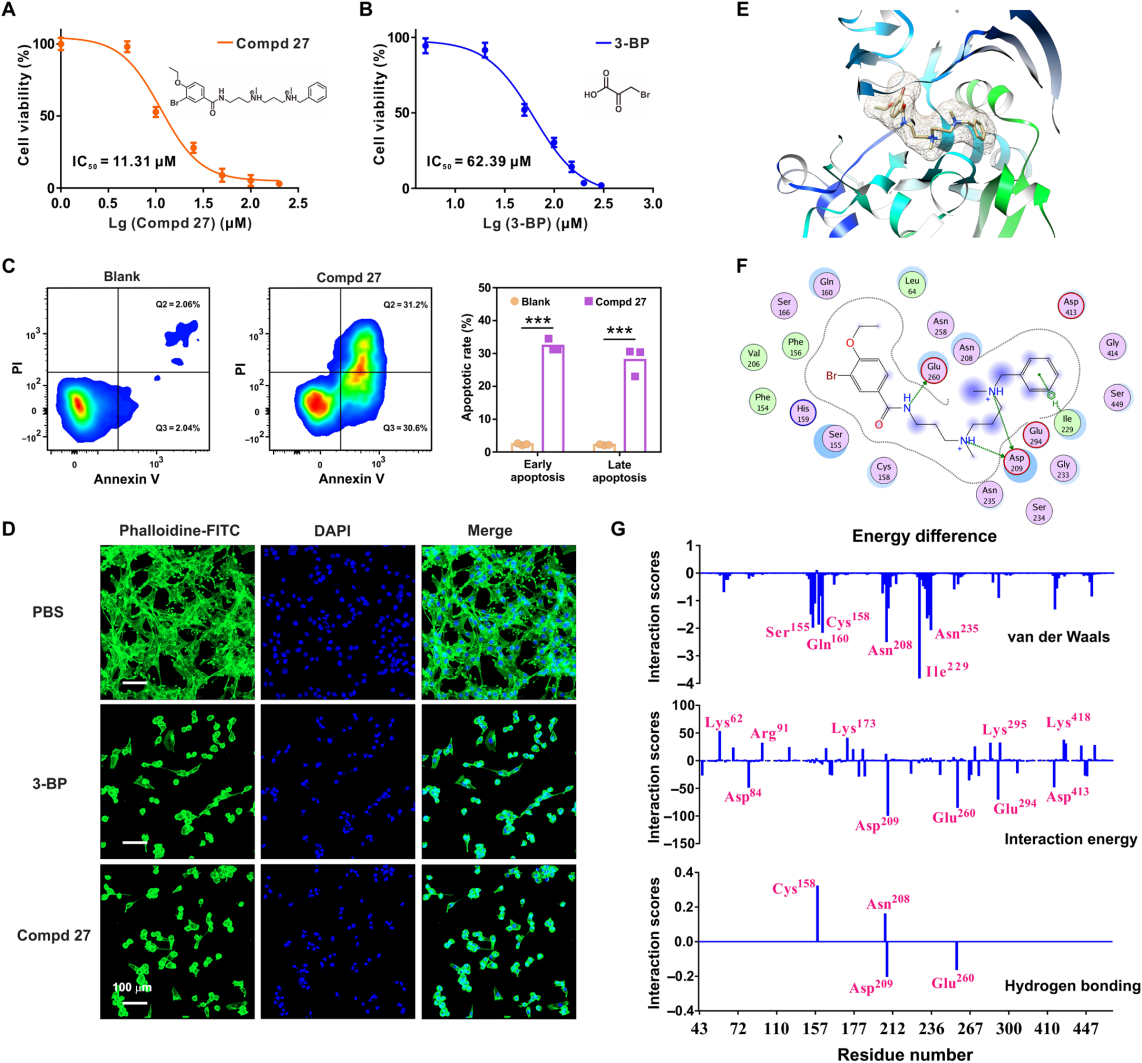
Glycolytic inhibiting activities of Compd 27. (**A** and **B**) Antiproliferation activity of Compd 27 or 3-BP (the known standard control as the HK2 inhibitor) against glioma U87 cells (*n* = 6). (**C**) U87 cell apoptosis triggered by Compd 27 with flow cytometry analysis. Cell staining by annexin V–FITC/PI. Statistical significance was calculated using the unpaired two-tailed Student’s *t* test. ***P < 0.001 (*n* = 3). (**D**) Fluorescent images of U87 cells treated by Compd 27 and 3-BP. Cell nuclei–stained by 4′,6-diamidino-2-phenylindole (DAPI) (blue); cytoskeleton stained by phalloidine-FITC (green). Scale bar, 100 μm. (**E**) Schematic representation of the binding structures of Compd 27-HK2 complex predicted by Glide XP docking simulations. (**F**) Two-dimensional schematic diagram of the binding patterns of Compd 27-HK2 complex, highlighting hydrogen bonds and dominant hydrophobic interactions. Color codes: pink (polar amino acids); green (greasy residues). (**G**) Differences of the interaction scores (Eint_Compd 27_ − Eint_3-BP_) between Compd 27 and 3-BP on a per-residue basis with the important residues highlighted. Calculations of total interaction energy, van der Waals interaction, and hydrogen bonding scores between the ligands and the protein residues by Glide module in Schrödinger.

The top five compounds ranked in the HK2 inhibitor screening (Compds 8, 11, 13, 21, and 27) were further tested against the proliferation of U87 cells. After incubation with the individual compounds in serial dilutions from 500 to 0.5 μM, the cell viabilities were evaluated with the CTG (CellTiter-Glo) assays. As shown in fig. S7, the results suggested three tiers of antiproliferation efficiency among this panel of compounds, including tier I: Compd 27 with an outstanding inhibition in U87 proliferation (> 90%, *P* < 0.05) at the least concentration of 50 μM; tier II: Compd 8 and the traditional inhibitor (3-BP as a control); and tier III: Compds 11, 13, and 21. In Fig. 2 (A and B), the median inhibitory concentration (IC_50_) values of Compd 27 and 3-BP against U87 proliferation were further determined, indicating a notable improvement by nearly fivefold (11.3 μM for Compd 27; 62.4 μM for 3-BP). Reverse transcription quantitative polymerase chain reaction (PCR) was used to profile the HK2 gene expression of the glioblastoma cell lines, including LN229, U251, and U87 cells. The results suggested that the effectiveness of Compd 27 on glioma cell suppression was positively correlated to the cellular HK2 expression levels (fig. S8). The flow cytometry data revealed the apoptotic responses of U87 cells resulting from the glycolytic pathway blockage by Compd 27 (Fig. 2C). Quantitative analysis of the staining with annexin V/propidium iodide verified the cellular membrane damages and phosphatidylserine exposure, indicating a high glycolytic inhibitory potency of Compd 27 on U87 cells. There were two primary cell subpopulations in the flow cytometry analysis: The early-stage [annexin–fluorescein isothiocyanate–positive (FITC^+^)/propidium iodide–negative (PI^−^)] and late-stage (annexin-FITC^+^/PI^+^) apoptotic cells treated by the inhibitor of Compd 27 accounted for 31.2 and 30.6%, respectively, while in the blank control, the percentages of U87 cells in these two stages were only 2.06 and 2.04%, respectively (Fig. 2C). Because of the effect of the glycolytic inhibition, the U87 cellular shape was notably changed from spreading/interweaving (the blank control) to rounded polygonal shrinkage (with the treatment of Compd 27 or 3-BP) observed under a confocal fluorescence microscopy (Fig. 2D), indicating the characteristic morphology of apoptosis.

As a mechanistic revelation of the inhibitory effect, the interactions between HK2 and Compd 27 or 3-BP were investigated using the Glide XP docking simulations (Fig. 2, E and F, and figs. S9 and S10). As shown in Fig. 2 (E and F), the active site residues Phe^156^, His^159^, Ser^155^, Cys^158^, Asn^235^, Asp^209^, Glu^294^, Ile^229^, Asn^208^, and Glu^260^ of HK2 were involved in the binding events to Compd 27. The interactions of Asp^209^, Ile^229^, and Glu^260^ were determined to be most critical for the binding between HK2 and Compd 27. A hydrogen bond was observed between Glu^260^ and the nitrogen atom of amide, and two hydrogen bonds were formed with the residue Asp^209^. Hydrophobic contacts (arene C-H interaction) between the residue Ile^229^ and the benzene ring of Compd 27 also contributed to the binding. Differences of the binding mechanisms between Compd 27 and 3-BP were also presented on a per-residue basis (Fig. 2G and fig. S11). Total interaction energy, van der Waals interaction, and hydrogen bonding scores between ligand and protein were predicted by including per-residue interaction scores in Glide. The differences of the interaction scores (Eint_Compd 27_ − Eint_3-BP_) were calculated to determine the unique binding features of Compd 27 in comparison to 3-BP. The van der Waals interactions of the residue Ile^229^ were revealed to be much stronger in the binding to Compd 27 than 3-BP, in well accordance with the observation of intensive arene-H interactions between Compd 27 and the residue Ile^229^. The interactions of Asp^209^ and Glu^260^ residues, featuring multiple hydrogen bonds between Compd 27 and both residues (Fig. 2, F and G), were identified to be the top two contributors in determining the difference of total interaction energy. The predicted hydrogen bonding scores also indicated that the interactions of Asp^209^ and Glu^260^ were only observed in the binding to Compd 27 rather than 3-BP.

### Regulation of the reprogrammed metabolomic pathways of U87 cells

**Fig. 3.**
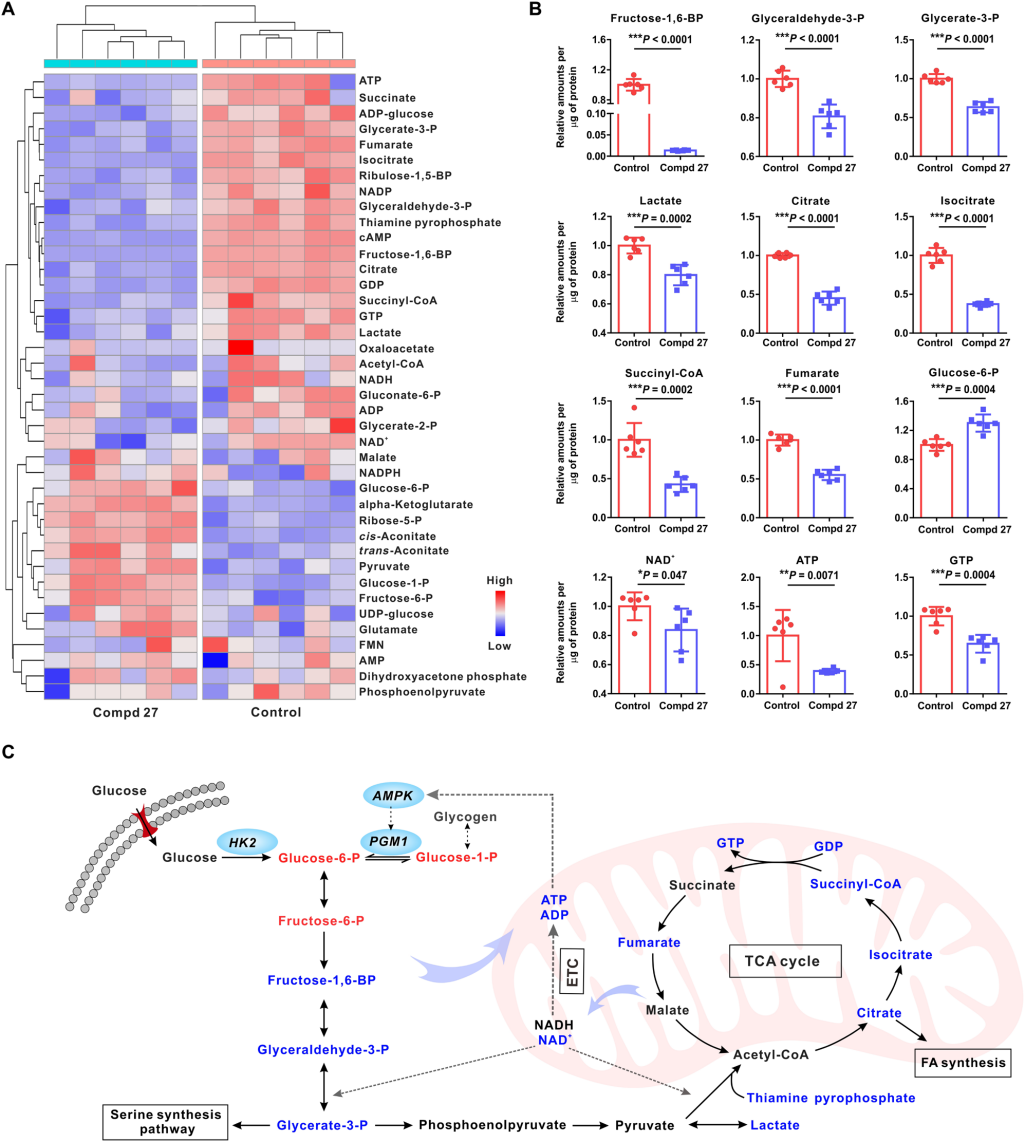
Regulation of the reprogrammed metabolomic pathways of glioma U87 cells with the treatment of Compd 27. (**A**) Relative difference heatmap of the clustered metabolites in U87 cells treated with/without Compd 27 (the blank control). The number of biological repeats *n* = 6. (**B**) Quantitative analysis of the metabolomic changes of U87 cells in the glycolysis and TCA cycle, with/without the treatment of Compd 27. Color codes: red (Compd 27); blue (PBS control). Statistical significance was calculated using the unpaired two-tailed Student’s *t* test. *P < 0.05; **P < 0.01; ***P < 0.001. (**C**) Visualization of differential glycolytic metabolites upon Compd 27 inhibition. Color codes: red (increased metabolites); blue (decreased metabolites).

The changes in the glucose metabolic pathways of U87 cells were monitored with a targeted metabolomic analysis (Fig. 3 and fig. S12) (*27*). We observed significant changes of the reprogrammed metabolomic pathways in the U87 glioma cells treated with the HK2 inhibitor candidate (Compd 27), featuring distinctly different metabolite clusters against the phosphate-buffered saline (PBS)–treated control (Fig. 3A). In the panel of down-regulated metabolites by treatment of Compd 27, we highlighted 12 important substances in the glycolysis and subsequent pathways, including tricarboxylic acid (TCA) cycle, serine synthesis pathway (SSP), and fatty acid (FA) synthesis (Fig. 3, A and C, and fig. S12). The decrease of fructose-1,6-BP, glyceraldehyde-3-P, glycerate-3-P, and lactate suggested that glycolysis of U87 cells was inhibited by Compd 27. The key metabolites in the TCA cycle, such as citrate, isocitrate, succinyl–coenzyme A (CoA), and fumarate, were also significantly decreased. All of these changes led to lowering the fundamental energy reservoir of tumor growth. The previous reports suggested that glycerate-3-P was also an important metabolic intermediate in SSP (*28*). After the treatment of Compd 27, the significant decrease (*P* < 0.0001) of glycerate-3-P revealed that the U87 cells suffered from the deficiency in de novo synthesis of serine and glycine. The lockout of citrate production in TCA cycle (*P* < 0.0001) further suppressed FA synthesis in the U87 cells, leading to the shortage of lipids against the requirements of tumor cell activities. Thiamine pyrophosphate (TPP) is a critical coenzyme for the conversion of pyruvate to acetyl-CoA by regulating the activities of pyruvate dehydrogenase (*29*). In comparison to the PBS control, the treatment of Compd 27 resulted in the decrease of TPP and nicotinamide adenine dinucleotide (NAD^+^, a critical cofactor of the reaction), thus reducing the glucose metabolic flux to TCA (*29*, *30*). The slight increase of glucose-6-P may be derived from accelerated glycogen consumption through the adenosine monophosphate–activated protein kinase (AMPK) pathway, which served as a characteristic compensatory mechanism of tumor cells under severe ATP depletion (*31*). The ATP level was significnatly lower in U87 cells induced by Compd 27 in comparison to PBS control (*P* < 0.01). Together, the data revealed the important changes influencing the abnormal metabolomic pathways of U87 cells after the treatment of Compd 27 (Fig. 3C).

We also compared the metabolic changes of U87 cells treated with 3-BP and Compd 27 with a targeted metabolomic analysis (figs. S13 to S18). As a note, given the fact that IC_50_ value of 3-BP was more than fivefold higher than that of Compd 27, we decided to test a larger dosage of 3-BP (80 μM, four times greater than the dosage of Compd 27) on U87 cells to compensate the lower efficiency of 3-BP in tumor suppression (*32*). The metabolomic data suggested that both Compd 27 (with dosage of 20 μM) and 3-BP (with dosage of 80 μM) can reduce the glycolytic pathways and suppress tumor cell growth. However, there also existed remarkable metabolic differences after U87 cells were treated with Compd 27 or 3-BP (fig. S13). Compd 27 exhibited higher suppression efficiency than 3-BP, according to the profiles of a number of the key metabolites (lactate, TPP, acetyl-CoA, succinyl-CoA, and fumarate) in the glycolytic pathways and TCA cycle. The ultimate level of ATP in U87 cells was significantly lower with the treatment of Compd 27 than that of 3-BP (*P* < 0.0001). The treatment of 3-BP rendered several metabolites to be lowered less than Compd 27, such as fructose-6-phosphate, fructose-1,6-bisphosphate, glyceraldehyde-3-phosphate, and glycerate-3-phosphate, but with the dosage four times greater than the latter. Therefore, the metabolomic data supported that Compd 27 can suppress tumor glycolysis and deprive the tumor cells of their energy supplies.

The effects of Compd 27 regulating the reprogrammed metabolisms of tumors in vivo were further investigated using the tumor specimens from subcutaneous U87 xenografts for targeted metabolomic measurements (figs. S19 to S21). The experiments revealed important metabolomic changes in the subcutaneous U87MG tumors due to the treatment of Compd 27, in contrast to the PBS control. Overall, these changes in vivo were consistent with our findings based on the in vitro experiments using U87 cells, evidenced by the significant metabolic decreases, such as glyceraldehyde-3-P, lactate, citrate, isocitrate, succinyl-CoA, and ATP in the glycolysis and subsequent pathways. The lowered NADH and NADP by Compd 27 treatment weakened the redox homeostasis of the tumors, thus limiting their repair of DNA under chemotherapeutics (*33*). The phenomenon of glucose-6-P increasing was observed in the in vivo metabolomic studies once again, indicating the activation of AMPK pathway–mediated compensation under ATP depletion. Both in vitro and in vivo metabolomic investigations confirmed the high effectiveness of Compd 27 in regulating the reprogrammed metabolisms of U87 as the glycolytic inhibitor.

### Pharmacokinetic profiling of the HK2 inhibitor by GLMSD

**Fig. 4.**
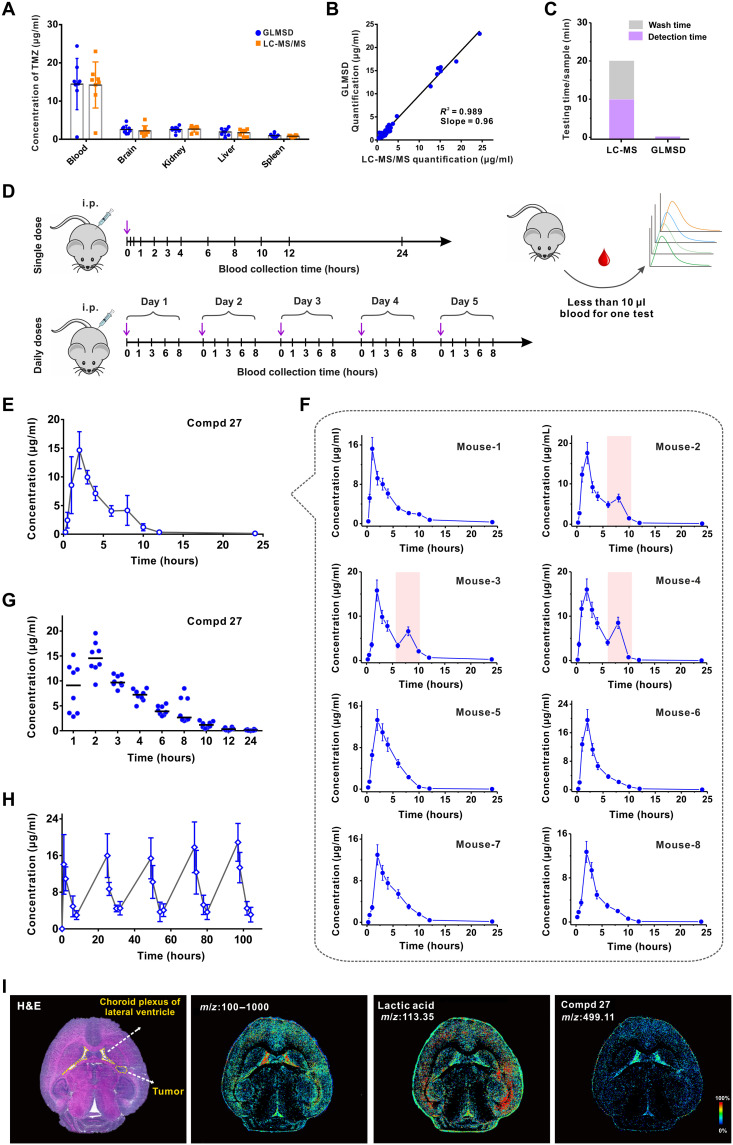
Pharmacokinetic studies of the small molecular compounds by GLMSD. (**A**) Quantification of TMZ in the plasma and different mouse organs by GLMSD or by traditional LC-MS/MS. The biological samples were obtained from eight mice at 3 hours after abdominal administration of TMZ with a dosage of 40 mg/kg. (**B**) Correlation assessment between GLMSD and LC-MS/MS in the tests of TMZ concentrations for varieties of biological samples from mice. (**C**) Comparison of the standard processing time between GLMSD and LC-MS/MS for each sample. (**D**) Schematic diagram of the drug administration in vivo and acquisition of the blood samples during the experimental time course. i.p., intraperitoneally. (**E**) The averaged profiles of Compd 27 concentrations in plasma verse time after intraperitoneal administration with a dosage of 40 mg/kg. Error bar, SD (mouse replicates *n* = 8). (**F**) The pharmacokinetic profiles of Compd 27 in each mouse. Eight mice were tested in parallel. Reabsorption of the drug was highlighted by the pink shades. (**G**) The pharmacokinetic profiles of Compd 27 by GLMSD, after the last dose of TMZ (intraperitoneally) for 5 days successively. Each dosage: 40 mg/kg. Mouse replicates *n* = 8. (**H**) The averaged pharmacokinetic profiles of Compd 27 by GLMSD in mice (*n* = 8) in the successively multiple-dose treatment (intraperitoneally). Each dosage: 40 mg/kg. (**I**) Mass spectral imaging of Compd 27 (*m*/*z* 499.11) and the other metabolites (e.g., lactic acid, *m*/*z* = 113.35) in the brain tissue slice, with an optical micrograph of an H&E-stained consecutive slice as a reference. Yellow dashed rings, the tumor site in an orthotopic mouse brain tumor model or lateral ventricle in the brain section. Signal intensities encoded with a color bar. Image resolution, 10 μm.

To verify the performance of GLMSD in pharmacokinetic study of small molecular drugs, we started with the tests using TMZ because of the facts as follows: (i) Isotope-labeled TMZ was commercially available as a convenient internal standard for quantification; (ii) pharmacokinetic profiles of TMZ documented in the literature reports could serve as good references for our tests; and (iii) TMZ was the chemotherapy of our choice in combination with the HK2 inhibitor to treat glioblastoma in the in vivo experiments. As shown in fig. S22, we demonstrated that GLMSD can sensitively measure the concentration of TMZ (*m*/*z* 216.97) with the limit of detection of 5.2 fmol in mouse plasma, free from the interference of high salt contents during sample conservation. The titration experiments by adding isotopic-labeled TMZ-D3 as the internal standard produced accurate and precise results to quantify TMZ, with a linearity (*R*^2^ = 0.995) in the concentration range from 0 to 50 μg/ml. The concentrations of TMZ in mouse plasma and organ tissue homogenates (brain, kidney, liver, and spleen) were cross-checked by both GLMSD and LC–tandem MS (LC-MS/MS) (end-point measurements at 3 hours after intraperitoneal administration of TMZ, mouse number *n* = 8), suggesting that they were in good agreement with each other (relative percentage difference <8%; Fig. 4A). The averaged concentration of TMZ in mouse blood was approximately 15 μg/ml. We observed large individual mouse variations in the blood-drug concentrations, which were consistently measured by both GLMSD and LC-MS/MS. A correlation analysis was performed for the data points identically collected by these two methods, demonstrating a linear relationship (*R*^2^ = 0.989, slope = 0.96; Fig. 4B). These experiments suggested that GLMSD reliably allowed for accurate quantification of drug concentrations in blood or organ tissues for pharmacokinetic study. In addition, a marked decrease (nearly 120-fold difference) of the instrument operation time was achieved in GLMSD (10 s) versus in LC-MS (20 min) for each MS data point, according to the standard procedures (Fig. 4C).

We further demonstrated the competitive advantages of GLMSD for pharmacokinetic study by tracing the complete drug-blood concentration profiles within each individual and simultaneously minimizing the total number of mice used in the tests. Because of the low consumption of the blood sample (<10 μl blood at each time point) featured by the GLMSD method, an entire pharmacokinetic curve of the drug (11 time points for blood sampling in a period of 24 hours, with a minimal interval of 0.25 hours) can be accomplished using one mouse (Fig. 4D). The pharmacokinetic profile of TMZ (fig. S23) averaged from eight mice (*n* = 8) provided the detailed values of *C*_max_ (the peak plasma drug concentration) (33.66 μg/ml), *T*_max_ (the time of peak plasma drug concentration) (0.5 hours), half-life (*T*_1/2_) (1.72 hours), and area under the curve at 0 to 24 hours (AUC_0–24 hours_) (247.05 μg/ml·hour), which were consistent with the previously reported data (*34*, *35*). The TMZ pharmacokinetic details of each mouse and statistic summary are available in the Supplementary Materials, including two modes of drug administration (single dose or successively multiple doses; figs. S23 and S24).

The pharmacokinetic profiles of Compd 27 were obtained by our GLMSD method after the mice (*n* = 8) were administrated orally with a single dose of 40 mg/kg (Fig. 4, D to G, and fig. S25). They provided the critical pharmacokinetic values of this inhibitor, including *C*_max_ (14.65 μg/ml), *T*_max_ (2 hours), *T*_1/2_ (4.12 hours, and AUC_0–24 hours_ (64.36 μg/ml·hour). Figure 4F presented the detailed plasmatic concentrations of Compd 27 during a period of 24 hours. Characteristic reabsorption peaks of this small molecular inhibitor were identified near the time point of 8 hours in three mice (nos. 2, 3, and 4) while mostly absent in the other five mice. This remarkable phenomenon was associated with drug-uptake difference between individuals, indicating that Compd 27 might regain the access to the blood circulation through a secondary pathway of absorption. Noteworthy, the reabsorption feature in pharmacokinetics was easily masked in the averaged data curves if the conventional MS techniques were applied because the other five mice did not exhibit reabsorbing the inhibitor. In contrast, the advantage of tracing an entire profile of blood-drug concentrations in each mouse by GLMSD can provide researchers with a better tool to clarify the individual difference in pharmacokinetics (Fig. 4G). In the mode of successive multiple doses, the pharmacokinetic data of Compd 27 were collected from each mouse daily (at 1, 3, 6, and 8 hours after the last dose) for 5 days. In Fig. 4H and fig. S26, there were repeatable patterns of plasmatic concentration changes of Compd 27 over the test period of 5 days, with the key pharmacokinetic values in each cycle similar to those in the single-dose test. The results indicated a relatively fast clearance of the inhibitor, minimizing undesired accumulation of the compound in blood circulation on daily dosages. Noteworthy, the conventional LC-MS techniques are limited to acquire a single data point of drug pharmacokinetics by sacrificing one mouse usually. However, GLMSD can break through this limitation by providing a complete pharmacokinetic profile based on a single mouse, no matter in single-dose (Fig. 4E) or successive multiple-dose (Fig. 4H) formats of drug administration.

The distribution of the inhibitor [Compd 27 after intraperitoneal administration for 2 hours] in the U87 xenograft mouse brain tissue slices was mapped by GLMSD as an MS imaging (MSI) technique (Fig. 4I). The hematoxylin and eosin (H&E)–stained consecutive tissue slice in the longitudinal direction served as a reference, indicating the U87 cell–implanted tumor site at the right frontal lobe of the brain. The MS signal intensities were coded by different colors in the heatmap image panels, respectively correlating to all the small molecules in the range of *m*/*z* 100 to 1000 (before deconvolution) on the left, lactic acid at *m*/*z* 113.35 [M + Na]^+^ in the middle, and Compd 27 at *m*/*z* 499.11 [M + Na]^+^ on the right. Here, we demonstrated in situ visualization of these target small molecules in brain tumor with an ultrahigh resolution of 10 μm by GLMSD. The distribution pattern of small molecules before deconvolution suggested the contour edges of corpus callosum and hippocampus. As an important metabolite of tumor glycolysis, lactic acid (*m*/*z* 113.35) was present surrounding the implanted glioma site notably higher than the other brain tissues. It contributed to the acidic tumor microenvironment, being consistent with the well-documented observations in the literature (*36*). The infiltration of Compd 27 in the implanted glioma site was revealed by our method, which suggested a good permeability of this small molecule to break through the blood-brain barrier (Fig. 4I). In addition, the presence of Compd 27 in the lateral ventricle might be attributed to the pooling of this chemical through the blood–cerebrospinal fluid (CSF) barrier. It could also be transported to the arachnoid by CSF circulation (*37*). Our MSI data demonstrated that intraperitoneal injection of Compd 27 led to good absorption into mammalian blood, allowing for effectiveness of the drug molecules in the gliomas by penetrating the blood-brain barrier.

### Efficient tumor suppression in vivo by HK2 inhibitor–assisted chemotherapy

**Fig. 5.**
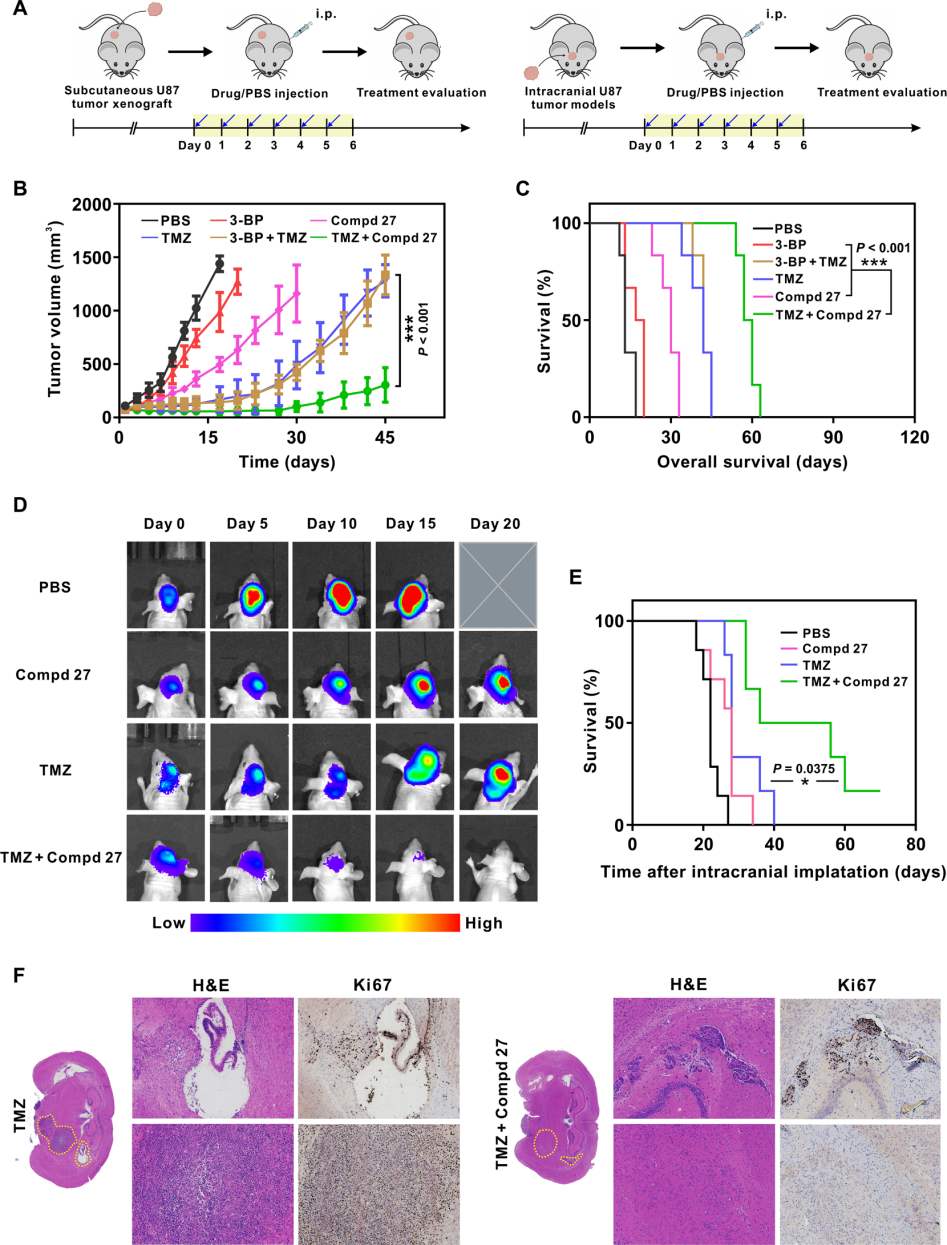
U87 glioma suppression by Compd 27 in both a subcutaneous tumor mouse model and an orthotopic mouse brain tumor model. (**A**) The schematic drawing of the in vivo experiments in these two mouse models. (**B**) Subcutaneous U87MG tumor growth curves after various treatments as specified. Tumor volume: means ± SD (*n* = 6). (**C**) Survival rates of mice bearing subcutaneous U87MG tumors after various treatments (*n* = 6). (**D**) Bioluminescent images of tumor progression in orthotopic U87-luciferase mice after various treatments (*n* = 7). Note: The mouse treated by PBS was dead before day 20. (**E**) Survival of mice bearing orthotopic U87MG tumors after various treatments (*n* = 7). (**F**) Representative H&E staining and Ki67 immunohistochemical staining of the intracranial tumors treated with the TMZ or TMZ + Compd 27. Statistical significance was calculated using paired two-tailed Student’s *t* test. *P < 0.05; ***P < 0.001.

Two types of U87 xenografts were used to evaluate the performance of the Compd 27 in tumor suppression, including a subcutaneous tumor mouse model and an orthotopic mouse brain tumor model. After the U87 xenografts were successfully harvested and randomized into different groups, the drugs were intraperitoneally administrated successively in the first 5 days, according to a standard dosing schedule (Fig. 5A). In the experiments of using subcutaneous U87 xenografts (Fig. 5B), there was a trend of increasing efficiencies in tumor volume suppression (TMZ > Compd 27 > 3-BP > PBS) as single-drug therapies. The active metabolite, 5-(3-methyltriazen-1-yl) imidazole-4-carboxamide, derived from TMZ can quickly induce alkylation of guanines in nucleic acids to disrupt the tumor cell proliferation. As the inhibitors of HK2, Compd 27 and 3-BP can reduce the energy supply of tumor cells. Their single-drug therapeutic effects for tumor ablation were milder than TMZ. We performed experiments to test the cytotoxicity of Compd 27 on four normal cell lines, namely, HET-1A, HUVEC, 3T3, and 293 T cells. The results suggested that Compd 27 had a much lower toxicity on the normal cells than 3-BP (fig. S27). We monitored the body weights/survival rates of the mice after intraperitoneal injection with Compd 27 or 3-BP, which confirmed lower toxicity in vivo for Compd 27 (fig. S28). The relatively higher toxicity of 3-BP was attributed to its ability of alkylating various pivotal proteins nonspecifically in the cells, such as cell death regulatory proteins and the proteins in the Akt/mammalian target of rapamycin and the mitogen-activated protein kinase pathways (*38*). However, combination of TMZ and Compd 27 can be used to treat subcutaneous U87 xenografts with significantly improved tumor suppression (*P* < 0.001; Fig. 5B), compared against the treatment of TMZ alone or the treatment of TMZ plus 3-BP. In Fig. 5C, the mice treated with the combination of TMZ plus Compd 27 presented the best the overall survival among all the different experimental groups. It was verified by the median survival time of TMZ plus Compd 27 (58 days, color-coded with the green), significantly longer (*P* < 0.0001) than the other treatments, including normal saline (15 days, black), 3-BP (17 days, red), Compd 27 (30 days, purple), TMZ (35 days, blue), and TMZ plus 3-BP (35 days, brown). These experimental results indicated a good druggability of Compd 27 as the HK2 inhibitor, in combination with chemotherapeutic agents such as TMZ for cancer treatment.

Bioluminescence images of orthotopic U87 xenografts were acquired to monitor the intracerebral tumor suppression by different treatments. After measurements of bioluminescent intensities of U87 gliomas, we identified that the single-drug therapies of either TMZ or Compd 27 alone delayed the growth of gliomas in comparison to PBS. However, they only allowed for a limited success in avoiding glioma-induced mouse deaths during the first 20 days under the treatments. After this period, the survival rates of orthotopic U87 xenografts quickly dropped because of malignant gliomas in either single-drug groups. In sharp contrast, the combination of TMZ plus Compd 27 can significantly suppress the bioluminescent U87 gliomas and enhance the survival rate (Fig. 5D and fig. S29). The median survival time of this group was promoted up to 55 days, nearly doubled than the single-drug groups (28 days). After the orthotopic U87 xenografts were sacrificed at 28 days after tumor implantation, the glioma lesions were processed with paraffin sectioning to obtain H&E and Ki67 IHC images. The brain sections of the mice treated with TMZ alone were featured with the spread glioma regions and complicated malignant metastasis. However, the combined treatment of proliferation of glioma cells was effectively suppressed by the combined therapy (Fig. 5F). The notably improved therapeutic effect was attributed to the blood-brain barrier penetration, highly efficient normalization of the reprogrammed metabolism of glioma cells by Compd 27, and its synergistic effect on suppressing tumor cell DNA repair with the chemotherapy drug TMZ.

## DISCUSSION

GLMSD meets the requirements of high-throughput, rapid screening for drug development. As a label-free approach, it bypasses the typical blind-zone limitation of the screening methods based on fluorescence or UV-Vis absorption. In our experiments, the conventional screening method based on UV-Vis absorption was not applicable to 10 compounds of the pool of 37 chemical candidates because of the blind-zone problem, while GLMSD only missed none. In addition, GLMSD enables a rapid screening of the reactions (e.g., 306 tests per hour). For comparison, the screening rate of the conventional LC-MS techniques is usually in the range of 30 tests per hour. The LC-MS facilities equipped with ultrafast gradient short columns can reach an upper limit of approximately 170 tests per hour (*20*, *39*, *40*), only 56% of the screening speed of GLMSD.

GLMSD enables surveys of pharmacokinetic landscape of candidate drug molecules with an ultrahigh cost-effectiveness. The current LC-MS techniques rely on a large number of sacrificed animals to collect the data of a single pharmacokinetic curve. The issue of individual mouse-to-mouse variation compromises the data quality by introducing additional variables to the drug pharmacokinetics. In contrast, GLMSD allows for “one-pharmacokinetic-profile out of one-mouse” by fully minimizing the sampling volume (i.e., ≤10 μl in a single drop of blood). In this work, we have demonstrated the robust acquisition of the pharmacokinetic data using a single mouse in the drug administration mode of either single dose or successive multiple doses. Without GLMSD, the mouse sacrifice would have to be increased by 11- to 20-fold to collect the same sets of pharmacokinetic data, which would be terrible against animal welfare according to the 3R principle (reduce, replace, or refine animal usage) (*41*). According to the statistic estimation (*42*), more than 100 million mice of various models every year are sacrificed for medical tests all over the world. If the numbers of animal sacrifice can be reduced to ^1^/_10th_ with the help of new technology such as GLMSD, then the culture and usage of animal models will be revolutionized. GLMSD also offers the opportunity to reveal the mouse-to-mouse variation such as the secondary-absorption details of the drug, thus assisting translation of the pharmacokinetic research to clinical tests. Direct visualizing the HK2 inhibitor (Compd 27) and the other metabolites in the brain tissue sections by GLMSD can offer great benefits in understanding the organ distribution, tissue penetration, and metabolic behaviors of the drug candidates.

State-of-the-art artificial intelligence (AI) has emerged, penetrating various stages of drug development, such as identification and validation of drug targets, design of new compounds, prediction of drug safety and pharmacokinetic behaviors, and integration and deep data mining of clinical information to improve the R&D efficiency (*43*, *44*). However, there is an urgent need in this field to acquire high-quality, big data of drug tests for the iterative enhancement of AI training (*45*). GLMSD offers a versatile platform to break through the inhibitory thresholds in drug development, benefiting not only pharmaceutical giants but also start-ups and research-based small laboratories for synergistic acquisition of the big pharmaceutical data.

The successful discovery of HK2 inhibitors by GLMSD provides a new solution targeting malignant tumor metabolisms. The altered glucose metabolism in cancer cells, switching from mitochondrial respiration to aerobic glycolysis, is fundamental to meeting their requirements in energy and biomolecular synthesis (*1*, *46*, *47*). The metabolic factors promote the malignant evolution of the tumor microenvironment, the epithelial-to-mesenchymal transition, and avoidance of immune surveillance (*1*, *48*, *49*). Different compounds have been tested against cancer vulnerabilities by focusing on inhibition of HK2 to disrupt the glycolytic pathway of cancer cells. For instance, 2-DG, a glucose analog, is being tested in several clinical trials (phases 2 to 3) to assist anticancer therapies (*12*). However, the anticancer efficacy of 2-DG is compromised because of its side effect of activating the alternative prosurvival pathways of cancer cells (*50*, *51*). 3-BP has been proposed to suppress tumor angiogenesis as an antimetabolite attributed to its structural similarity to acetate, pyruvate, and lactate and its inhibitory effect on HK2 (*52*). There are several critical biochemistry problems against the clinical translation of 3-BP, evidenced by occasional preliminary clinic tests (*13*, *53*). The limitations of 3-BP include its rapid inactivation/drug resistance in glutathione-rich tumors, the off-target risk of interacting with the other unknown proteins due to its oversimplified chemical structures, and difficulty in crossing the blood-brain barrier to treat gliomas (*52*). The maximum dosage tolerance of mice to the chemicals in this study suggested that the toxicity of Compd 27 to healthy mice was much lower than 3-BP. Therefore, Compd 27 may exhibit additional therapeutic benefits by minimizing the undesired side effects. As a brand-new inhibitor to HK2 with higher binding affinity, Compd 27 can rescue the reprogrammed metabolisms of cancer cells, reducing the metabolite flux of both glycolysis and TCA cycle for proapoptosis of tumors. Furthermore, it can synergistically assist the chemotherapy of TMZ against DNA repair of cancer cells by inhibiting the PPP (pentose phosphate pathway) metabolic pathways. This compound exhibits attractive ability of penetrating the blood-brain barrier and targeting malignant glioblastoma with low side effects on normal tissues, thus being able to bring great benefits in clinical research.

In summary, we have developed a state-of-the-art platform (GLMSD), which addresses the critical challenges of drug development, including high-throughput drug screening and high-quality pharmacokinetic data acquisition. GLMSD enables identification of five brand-new chemical scaffolds for HK2/substrate interactions. The best inhibitor (Compd 27) features high efficacy in HK2 inhibition of glioma cells, effective penetration of the blood-brain barrier, and low side effects on normal tissues. Our work has revealed the biomolecular pathways of regulating the reprogrammed metabolisms of glioblastoma by using Compd 27. Efficient glioblastoma treatment has been demonstrated in both subcutaneous and orthotopic brain tumor models by synergizing Compd 27 and TMZ. In the future, the emergence of high-throughput, low-cost techniques of RNA sequencing or gene expression quantification may assist the decision of customizing combinations of HK2-dependent/independent inhibitors and chemical therapeutics to treat patients with cancer (*54*). The platform of GLMSD allows for rapid acquisition of individual pharmacokinetic profiles during the period of chemotherapies, promising critical information for the physicians to make customized choices of the drug dosages (*55*). Therefore, next-generation targeted therapies for personalized medicine can be inspired by the glycolytic inhibitor discovery with our approach.

## MATERIALS AND METHODS

### Materials

All chemicals were purchased from Sigma-Aldrich, Adamas Bate, or Beijing Chemical Reagent and used as received without further purification. Deionized water (18 megohms) from Milli-Q was used in all experiments. All the inhibitors used in the high-throughput screening experiment were purchased from Specs database, and the Hexokinase Assay Kit was purchased from Solarbio Co.

### Computer screening

The crystal structure of human HK2 with a 2-amido-6-benzenesulfonamide glucosamine compound [Protein Data Bank (PDB) entry, 5HFU] was obtained from RCSB (Research Collaboratory for Structural Bioinformatics) PDB (*56*) and used in HTVS campaign. Preparation of the protein structure was carried out using the module of Protein Preparation wizard in Schrödinger 9.0 (*57*). All crystal water molecules were deleted form the system, and hydrogen atoms and partial charges were added to the proteins. A restrained partial minimization was completed with the root mean square deviation value set to 0.3 Å.

A screening library of about 240,000 compounds was obtained from the Specs chemical library and prepared with the LigPrep module in Schrödinger. Protonated states were generated at pH = 7.0 ± 2.0. Default settings were used for the other parameters. All docking simulations were carried out using the Glide module in Schrödinger. The Protein grid box for docking was generated and centered on the inhibitor in the binding pockets. The scaling factors for van der Waals radii were set to 1.0, and the maximum partial atomic charge was set to 0.25. Last, all the compounds from the Specs library were docked into the binding pocket of HK2 with different precision modes, including SP and XP. The Epik state penalties were added to docking scores.

In the first step, all the compounds were docked into the structure of HK2, and the binding affinities were scored and ranked by Glide SP mode. Then, the top-ranked 50,000 molecules were saved and used in Glide XP docking. ACD/ADME package (*58*) was subsequently applied to predict the ADMET properties of the selected compounds, and the 1000 top-ranked compounds from Glide XP docking were then filtered to remove those failed to accommodate the following rules: (i) log*P*/log*D* (pH = 7.0) < 5.5; (ii) violation of Lipinski’s rules of five <2 (*59*); (iii) violation of Opera’s rules of drug-likeness <3 (*60*); and (iv) functional groups without toxic, reactive, or otherwise undesirable moieties defined by the REOS (Rapid Elimination of Swill) rules (*61*). The rest of the molecules were then clustered on the basis of the Tanimoto distance calculated from the FCFP_4 (Functional Connectivity Fingerprints 4) fingerprints using the Find Diverse Molecule module in Discovery Studio 2.5 (*62*). Forty hit compounds with the lowest docking scores were lastly selected and purchased from Specs.

A second-round virtual screening was performed to optimize the chemical scaffold of Compd 27 after identification of the best inhibitor candidate from the first-round HTVS and the preliminary cell experiments. We tested a larger database (ChemDiv) commercially available containing more than 10^6^ chemical compounds and calculated compound similarity on the basis of the Tanimoto distance predicted from the MACCS (Molecular ACCess System) structural keys fingerprints. An additional panel of six compounds with similarity (>80%) to Compd 27 were selected from the database of ChemDiv for subsequent systematic investigation on their binding potency to HK2 (indicated by the docking score) and other drug-like properties including *F*(20%), LogS, PPB, *T*_1/2_, H-HT, DELI, and Ames. Prediction of the ADMET properties was performed by ADMETlab 2.0 (*63*). After careful balance of the multiple parameters including the binding potency and drug-like properties, Compd 27 was still outstanding in the second-round screening. Therefore, Compd 27 was selected as the premier HK2 inhibitor candidate for the subsequent metabolomic studies and in vivo investigations.

### In vitro HK2 assays and inhibitor screening

The HK2 activity assay was performed in a reaction mixture (50 μl) composed of 10 mM glucose, 1.2 mM ATP, and 2.5 μl HK2 (0.1 mg/ml) in 25 mM tris-HCl, and 5 mM MgCl_2_ (pH 7.5). The reaction mixture was added with different concentrations of the inhibitor for incubation at 37°C for 60 min. The reaction was stopped by adding trifluoroacetic acid to a final concentration of 2% (v/v). Afterward, the reaction solution was diluted and mixed with 0.5 mM C^13^-glucose and dropped to the MTP 384 steel plate (Bruker Daltonics) precoated with GDs matrix. A high-resolution MALDI–time-of-flight (TOF) MS instrument (UltrafleXtreme, Bruker Daltonics) with Compass 1.3 control and processing software was used for MS data acquisition. The samples were tested in the automatic random sampling mode (AutoXecute, Bruker Daltonics, 4 to 6 s per spot). The detection was performed using an Nd:YAG laser (wavelength, 355 nm; laser pulse duration, 3 ns; laser power of 45%) with reflection in the positive-ion model. The spectral data were analyzed by FlexAnalysis software (v. 3.3, Build 80).

As for the control method using the traditional assay based on UV absorbance, the HK activity was examined by a commercial Hexokinase Assay Kit (Solarbio). The inhibitor was premixed with the samples and immediately added into the test buffer for incubation at 37°C for 10 min. The UV absorbance measurements at 340-nm wavelength were performed on all of the samples for screening.

### Reverse transcription quantitative PCR

RNA samples were extracted from U87, U251, and LN229 cells using the TRIzol reagent (Invitrogen, Thermo Fisher Scientific). The complementary DNA samples were prepared by HiScript III All-in-one RT SuperMix Perfect for qPCR (50°C, 15 min for incubation) (Vazyme). Quantitative PCR was performed for HK2 and β-actin genes using the thermocycler (CFX Connect, Bio-Rad). The primers were synthesized by GENEWIZ, including the oligo sequences as follows: HK2 (human) forward primer 5′-ACCCAGCTGTTTGACCAC-3′ and reverse primer 5′-CGAGAAGGTAAAACCCAG-3′; β-actin (human) forward primer 5′-AGCCTCGCCTTTGCG-3′ and reverse primer 5′-CTGGTGCCTGGGGCG-3′. The fluorescence during PCR was monitored with the Taq Pro Universal SYBR qPCR Master mix (Vazyme). The gene expression levels were normalized by the house-keeping gene β-actin.

### Pharmacokinetic profiling of TMZ or Compd 27 by GLMSD

In the single-dose mode, TMZ (40 mg/kg) or compound 27 (40 mg/kg) was administrated through intraperitoneal injection for each mouse (C57BL/6J). Blood samples were collected through tail vein in 1% heparin sodium–coated tubes at the following time points: pre-dose, including 0.25, 0.5, 1, 2, 3, 4, 8, 12, and 24 hours after drug administration (the number of biological repeats, mice *n* = 8). In the mode of continuous drug administration for 5 days (TMZ or compound 27, 40 mg/kg), blood samples were collected at 0, 1, 3, 6, and 8 hours on day 1 or at 1, 3, 6, and 8 hours on days 2 to 5 after each administration (the number of biological repeats, mice *n* = 8 for TMZ; mice *n* = 6 for Compd 27).

The plasma (10 μl) was separated and centrifuged at 2100*g* for 15 min. All samples were kept at −80°C before analysis. To keep the chemical stability of TMZ, we placed the plasma samples in an Eppendorf tube containing 8.5% 1 μl of phosphate acid and adjusted the pH value to 4. The solution containing the internal standard molecules (80 μl, 400 ng/ml methanol) and 80 μl 10 mM (pH 3.5) ammonium acetate buffer were added to the mouse plasma (40 μl), which were precipitated with 80 μl of methanol and 80 μl of 100 mM zinc sulfate. After mixing with TMZ-D_3_ (4 μg/ml; v/v, 1/1; *m*/*z*: 197.17) for 1 min and centrifugation at 10,000*g* for 15 min, the sample (1 μl) was dropped onto the steel plate for the analysis of MS.

The sample treatment to test Compd 27 in blood was similar to the description as above to test TMZ, except for the stabilizing step with phosphate acid. An analog (*m*/*z* 432.41) of Compd 27 was used as the internal standard for the mass spectral measurement.

Detection of TMZ or Compd 27 in the final serum samples was performed by the GLMSD platform according to a protocol described previously in the literature (*26*). Briefly, the GD solution (1 μl, 1 mg/ml) was dropped onto the steel plate of MALDI and dried at room temperature. The sample solution (1 μl) containing the analytes was deposited on the GD surface and dried at room temperature before MALDI MS tests. In addition, the standard sample pretreatment procedure for LC-MS (*34*) was applied to pretreat the blood samples or the homogenized organ tissues, including liver, spleen, kidney, and brain, after the mice (C57BL/6J) were administrated with TMZ as above (the number of biological repeats, mice *n* = 8).

### GLMSD platform for MSI

The dissected organs from the sacrificed mice were frozen by slow immersion in liquid nitrogen for 10 min and stored in a refrigerator at −20°C. The frozen tissues were sectioned at −20°C into 10-μm-thick slices in a Leica CM-1950 cryostat (Leica Biosystems). The tissue slices for MALDI MS imaging or IHC staining were prepared from the mouse brain. GDs (1 mg/ml) was dropped onto indium tin oxide–coated glass slices and dried at room temperature to form one layer of uniform thin film. The tissue sections were thaw-mounted on the surface of GD-4 and dried under vacuum at a pressure of 10 mbar for 30 min. They were imaged by a QuanIMAGE MALDI-TOF mass spectrometer (Intelligene Biosystems Co. Ltd) with an Nd:YAG laser (349 nm) at the spatial resolution of 10 μm per pixel in the positive-ion mode. QuanViewer (v1.0, Intelligene Biosystems Co. Ltd) software was used for MSI and tissue image reconstruction. The adjacent brain slices were also stained using a standard IHC procedure, followed by optical scanning for a reference.

### Targeted metabolomic analysis of U87 cells or subcutaneous U87MG tumors

This procedure was referred to the sample pretreatment protocol for LC-MS assays in the literature (*64*). Cells (1 × 10^7^) were harvested and mixed with ammonium bicarbonate (8.5 g/liter, pH 7.4), followed by centrifugation at 1000*g* for 1 min. The cell samples were resuspended with 100 μl of Milli-Q water after homogenization (MP Fastprep-24), followed by mixing with 1 ml of cold methanol:acetonitrile (1:1, v/v). The samples were stored in ice and sonicated for 60 min and then stored at −20°C for 1 hour to precipitate proteins. After centrifugation at 14000*g* at 4°C for 20 min, the supernatants were freeze-dried and stored at −80°C before the subsequent analysis.

Before ultra-performance liquid chromatography (UPLC)–MS/MS analysis, the sample was resuspended in 100 μl of acetonitrile:water (1:1, v/v) and then centrifuged for 10 min at 14000*g* and 4°C. The supernatant (50 μl) was collected and diluted with 100 μl of acetonitrile:water (1:1, v/v) before MS analysis.

Six-week-old nude mice were injected subcutaneously with U87 cells (4 × 10^6^) in 100 μl of PBS. When the subcutaneous tumors reached 100 mm^3^, the nude mice were administrated with intraperitoneal injection of Compd 27 (50 mg/kg) or PBS for 5 days continuously. The tumors were removed after intraperitoneal injection 3 hours at the fifth day and treated with liquid nitrogen for 15 min and then transferred to −80°C for preservation.

After 100 mg of each sample was grounded, 200 μl of water (4°C) and 800 μl of methanol:acetonitrile (1:1, v/v, 4°C) were sequentially added into the homogenized samples for mixing under ultrasonication in ice bath for 60 min. The samples were stored at −20°C before tests. The samples were centrifuged at 16000*g*, 4°C for 30 min to collect the supernatants. After spiking with equal volume of internal standard (l-Glutamate-D5) and followed by vacuum drying, each mixture was dissolved with 80 μl of acetonitrile aqueous solution (1:1, v/v). The samples were centrifuged at 16000*g*, 4°C for 15 min to prepare the supernatants.

The metabolomic data were acquired an Agilent 1290 Infinity LC system (Agilent Technologies) coupled with a 5500 QTRAP mass spectrometer (AB Sciex). By using the autosampler, 4 μl of sample was loaded at 4°C. The chromatographic separation was achieved on an ACQUITY UPLC BEH column (1.7 μm, 2.1 by 150 mm; Waters Technology Co. Ltd.) at a flow rate of 300 μl/min. For the mobile phase, solvent A was 15 mM ammonium acetate aqueous solution, and solvent B was acetonitrile. Chromatographic conditions of gradient elution went from 90% B down to 40% B in 18 min, followed by a sharp increase from 40% B up to 90% B in 0.1 min, and then a stable period at the volume ratio of 90% B for 4.9 min. The total time of the procedure was 23 min for each sample. Negative ionization and multiple reaction monitoring modes were used, of which the conditions were set as follows: source temperature, 450°C; nebulizer gas, 45; auxiliary gas, 45; curtain gas, 30; and ion spray voltage floating, −4500 V.

The metabolomics data were processed by MultiQuant software (AB Sciex). Peak detection and alignment of all samples were performed with reference to its corresponding standard substances (Sigma-Aldrich). The data matrix was uploaded to MetaboAnalyst 3.0 (*65*) for principal components analysis and hierarchical cluster analysis.

### Orthotopic U87-luciferase MG human glioblastoma tumor model

All animal care and experimental procedures were conducted according to the Guidelines for Animal Experiments of the School of Basic Medical Sciences of Soochow University. Female nude mice (6 weeks old) under specific pathogen–free conditions were provided by Laboratory Animal Center of Soochow University. After the step of anesthesia (intraperitoneal injection of chloral hydrate 5%, v/v), an incision 5 mm long was made along the mouse head midline. A hole (1 mm in diameter) was drilled into the skull at the right frontal lobe, 2.0 mm posterior, 2.0 mm lateral, and 3.0 mm ventral coordinates relative to bregma using a high-speed drill (Dremel Inc., USA). A syringe (25 μl, Hamilton) equipped with a 26-gauge needle was used to inject 10 μl of PBS containing 2 × 10^5^ U87-luciferase glioma cells into the junction between the cortex and striatum at a depth of 2.5 to 3.0 mm from the outer border of the cranium over a 5-min period. After injection, the needle was kept in place for 5 min before slowly being extracted to prevent a vacuum and cell buildup into the needle track. The mice after saturation of the wound were kept in a temperature-controlled environment with a 12-hour dark/12-hour light cycle and received a standard diet food and water ad libitum.

Bioluminescence imaging (small animal optical imaging system, Maestro EX, Photometrics, USA) was performed on the mice to validate U87 tumor grafts in the mouse brain. Mice carrying intracranial tumors were randomized to four groups for the following treatments: 0.9% normal saline, Compd 27 (50 mg/kg per day), TMZ (40 mg/kg per day), and Compd 27 (50 mg/kg per day) + TMZ (40 mg/kg per day) for 5 days in a row by intraperitoneal injection.

### Subcutaneous xenografts and drug treatment

U87 cells (4 × 10^6^) were suspended in 100 μl of PBS and injected subcutaneously on the back of each female nude mouse. Mice bearing 100-mm^3^ subcutaneous tumors were randomized to six groups to receive daily treatment as follows: (i) 0.9% normal saline; (ii) TMZ (40 mg/kg); (iii) Compd 27 (50 mg/kg); (iv) 3-BP (8 mg/kg); (v) TMZ + 3-BP; and (vi) TMZ + Compd 27, for 5 days continuously. The tumor diameters were measured with a caliper. The tumor volumes were calculated using the formula: 0.5 × length × width^2^. The mice were sacrificed when the tumors in the normal saline control reached the maximal size allowed by the Institutional Animal Care and Use Committee.

## Notes

View/request a protocol for this paper from *Bio-protocol*.
