# Supplementary material for: High-throughput glycolytic inhibitor discovery targeting glioblastoma by graphite dots–assisted LDI mass spectrometry
Source: Sci Adv. 2022 Feb 16;8(7):eabl4923. doi: 10.1126/sciadv.abl4923 (PMC10921956; doi:10.1126/sciadv.abl4923)
Supplement: Supplementary file 1 — Figs. S1 to S29 Tables S1 and S2 [file sciadv.abl4923_sm.pdf]

Science Advances
AAAS

Supplementary Materials for

High-throughput glycolytic inhibitor discovery targeting glioblastoma by graphite dots–assisted LDI mass spectrometry

Rui Shi, Peichen Pan, Rui Lv, Chongqing Ma, Enhui Wu, Ruochen Guo, Zhihao Zhao, Hexing Song, Joe Zhou, Yang Liu, Guoqiang Xu, Tingjun Hou*, Zhenhui Kang*, Jian Liu*

*Corresponding author. Email: jliu@suda.edu.cn (J.L.); zhkang@suda.edu.cn (Z.K.); tingjunhou@zju.edu.cn (T.H.)

Published 16 February 2022, *Sci. Adv.* **8**, eabl4923 (2022)
DOI: 10.1126/sciadv.abl4923

The PDF file includes:

Figs. S1 to S29
Tables S1 and S2

Other Supplementary Material for this manuscript includes the following:

Data files S1 and S2

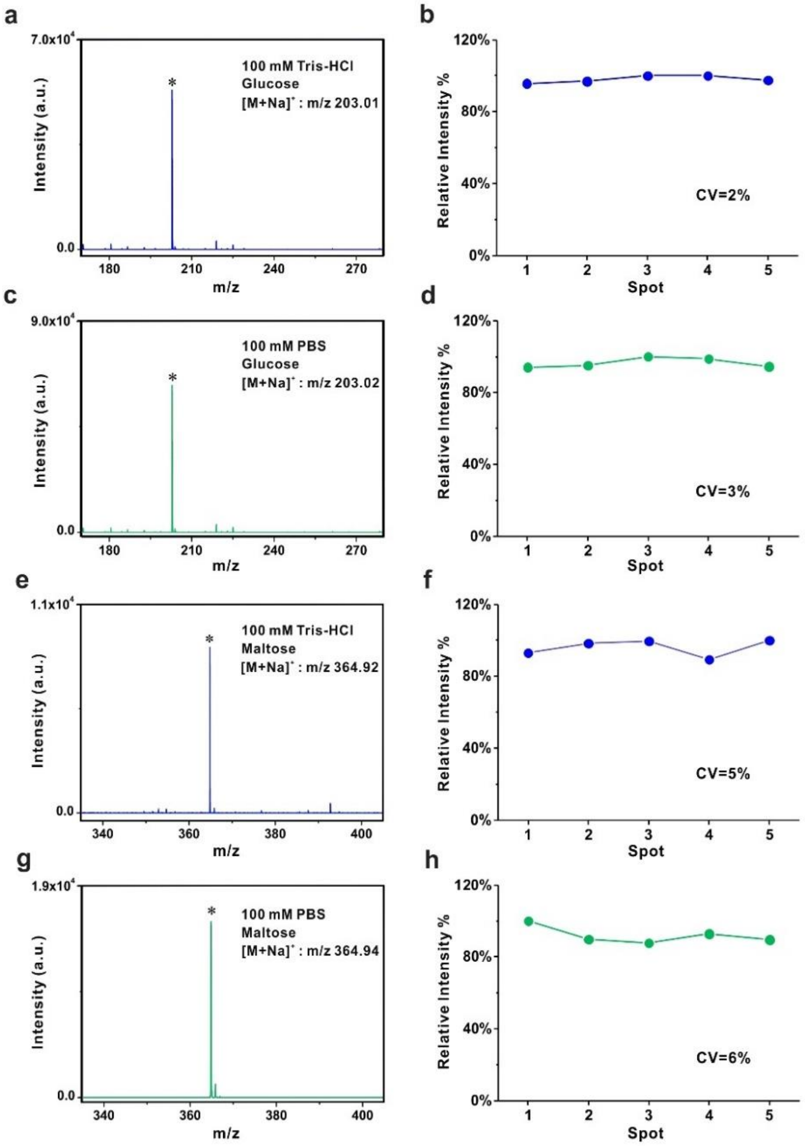

Fig. S1. Highly reproducible MS signal intensity by GLMSD. (a) The mass spectrum of glucose dissolved in 100 mM Tris-HCl buffer on positive-ion mode. Peak at m/z 203.01 [glucose + Na]$^+$. (b) Relative intensity at five different positions in a single spot of glucose/GDs. The coefficient of variation (CV) values was only 2% in the position-to-position tests. (c) The mass spectrum of glucose dissolved in 100 mM PBS on positive-ion mode. Peak at m/z 203.02 [glucose + Na]$^+$. (d) Relative intensity at five different positions in a single spot of glucose/GDs. The coefficient of variation (CV) values was only 3% in the position-to-position tests. (e) The mass spectrum of glucose dissolved in 100 mM Tris-HCl buffer on positive-ion mode. Peak at m/z 364.92 [maltose + Na]$^+$. (f) Relative intensity at five different positions in a single spot of maltose/GDs. The coefficient of variation (CV) values was only 2% in the position-to-position tests. (g) The mass spectrum of maltose dissolved in 100 mM PBS on positive-ion mode. Peak at m/z 364.94 [maltose + Na]$^+$. (h) Relative intensity at five different positions in a single spot of maltose/GDs. The coefficient of variation (CV) values was only 3% in the position-to-position tests.

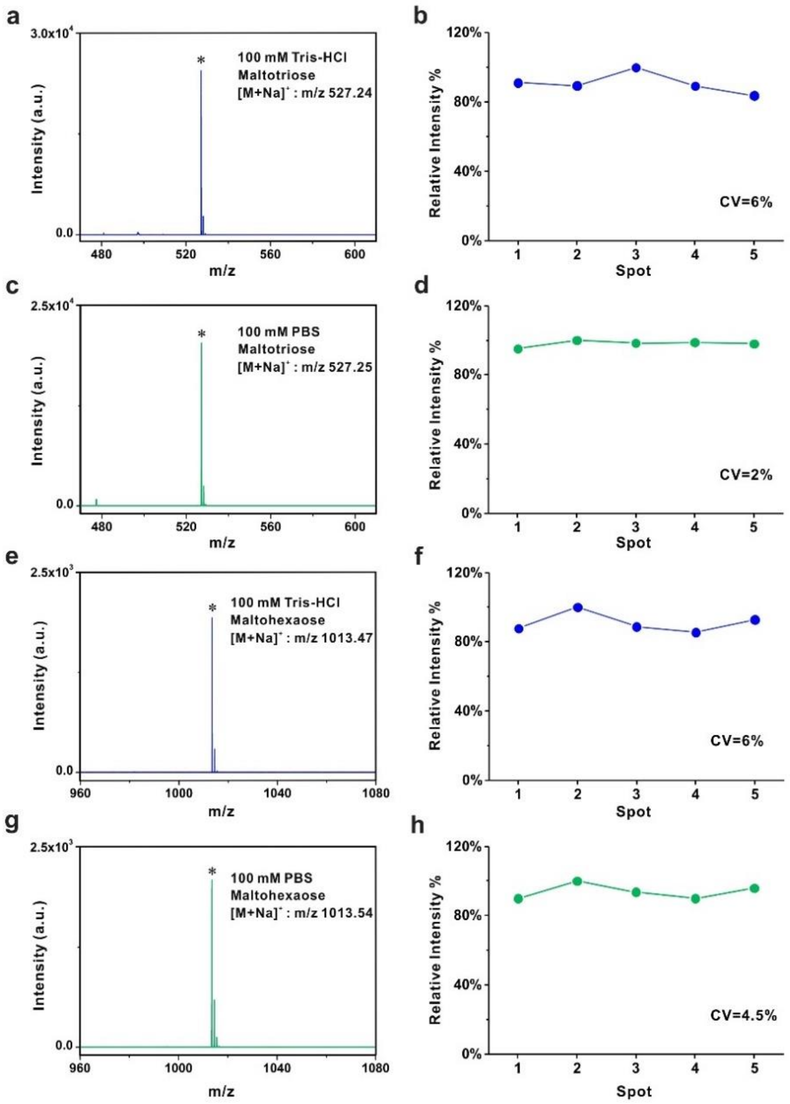

Fig. S2. Highly reproducible MS signal intensity by GLMSD. (a) The mass spectrum of maltotriose dissolved in 100 mM Tris-HCl buffer on positive-ion mode. Peak at m/z 527.24 [maltotriose + Na]$^+$. (b) Relative intensity at five different positions in a single spot of maltotriose/GDs. The coefficient of variation (CV) values was 6% in the position-to-position tests. (c) The mass spectrum of maltotriose dissolved in 100 mM PBS on positive-ion mode. Peak at m/z 527.25 [maltotriose + Na]$^+$. (d) Relative intensity at five different positions in a single spot of maltotriose/GDs. The coefficient of variation (CV) values was only 2% in the position-to-position tests. (e) The mass spectrum of glucose dissolved in 100 mM Tris-HCl buffer on positive-ion mode. Peak at m/z 1013.47 [maltohexaose + Na]$^+$. (f) Relative intensity at five different positions in a single spot of maltohexaose/GDs. The coefficient of variation (CV) values was 6% in the position-to-position tests. (g) The mass spectrum of maltohexaose dissolved in 100 mM PBS on positive-ion mode. Peak at m/z 1013.54 [maltohexaose + Na]$^+$. (h) Relative intensity at five different positions in a single spot of maltohexaose/GDs. The coefficient of variation (CV) values was 4.5% in the position-to-position tests.

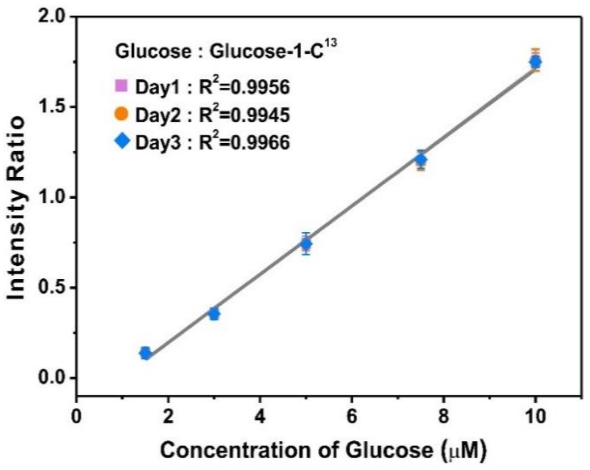

Fig. S3. Three calibration curves for quantitative analysis of glucose. D-glucose-1-^{13}C was used as the internal standard. Data are representative of three independent experiments. (n=3)

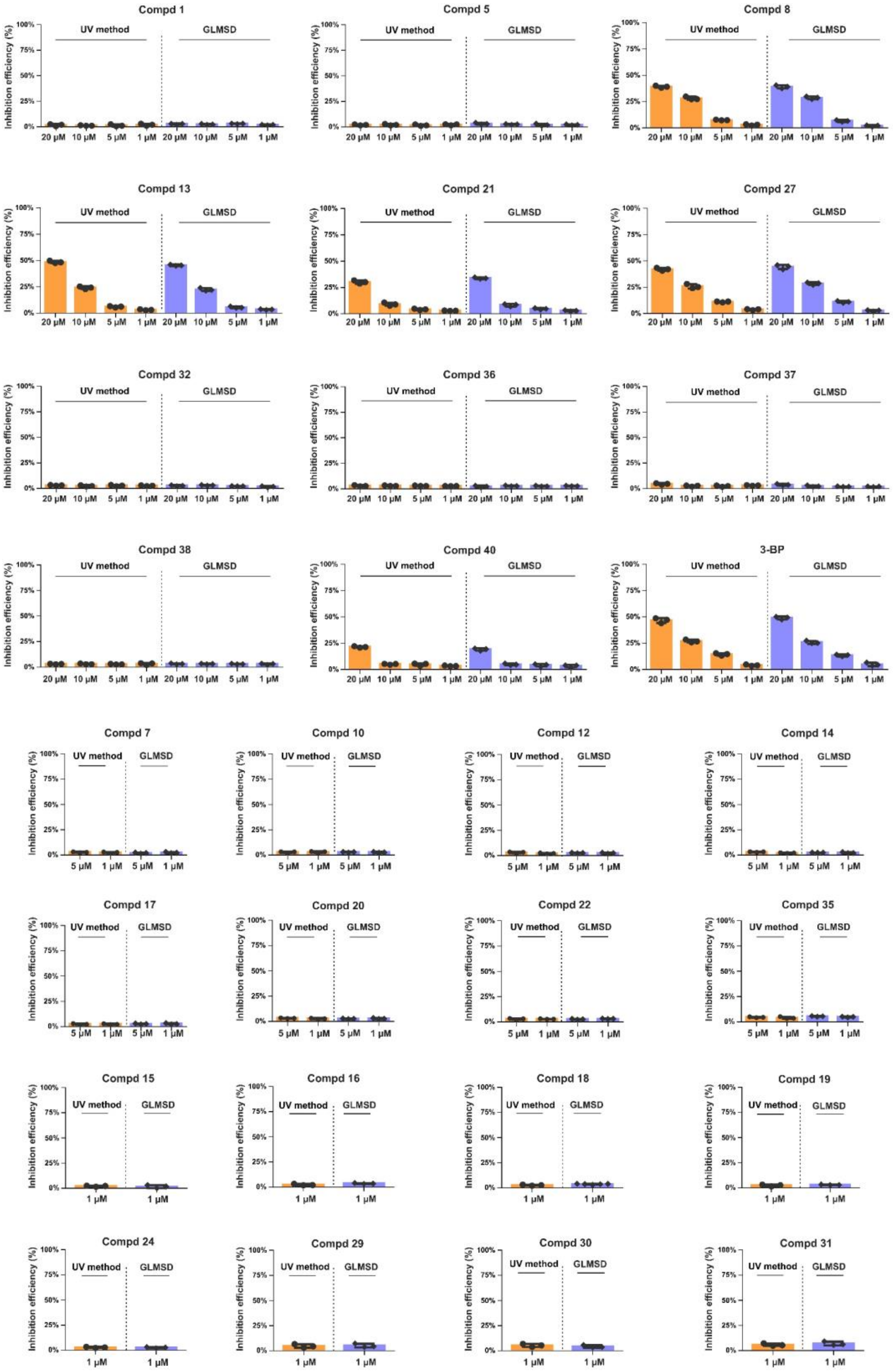

Fig. S4. Inhibition profiles of 28 inhibitor candidates that can be discovered by both UV-absorption method and GLMSD method. Data are representative of three independent experiments. (n=3)

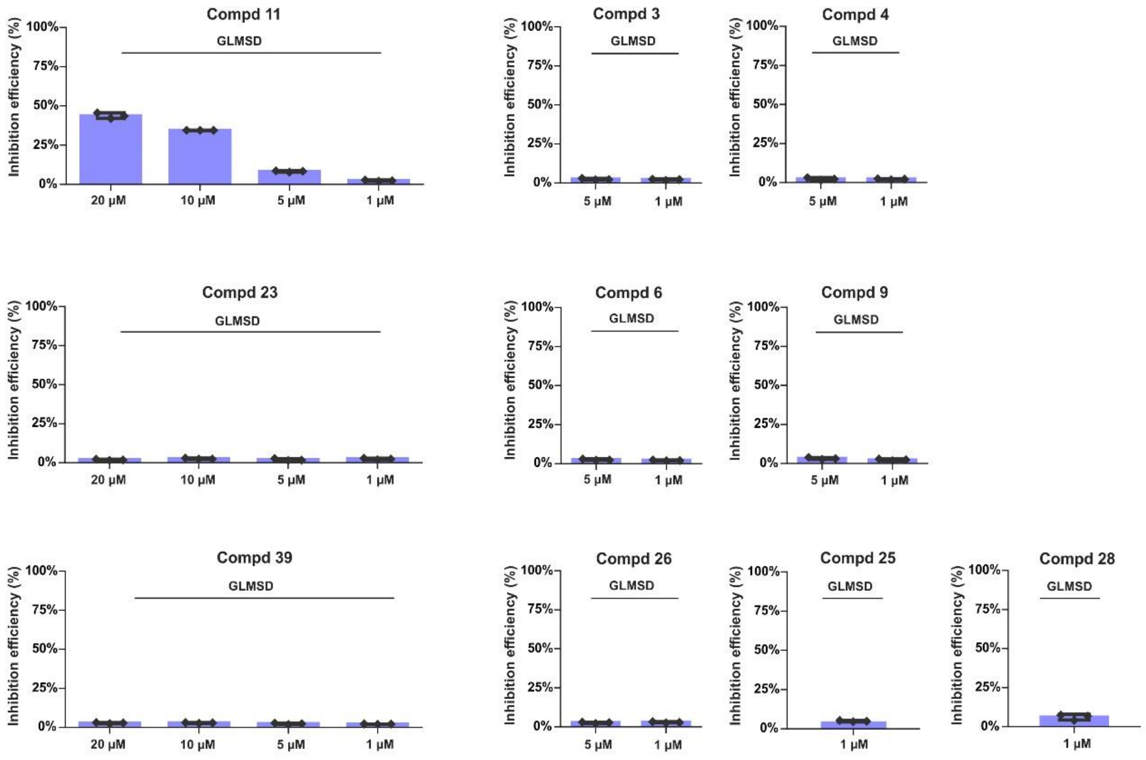

Fig. S5. Inhibition profiles of 10 inhibitor candidates that can only be discovered by GLMSD method. Data are representative of three independent experiments. (n=3)

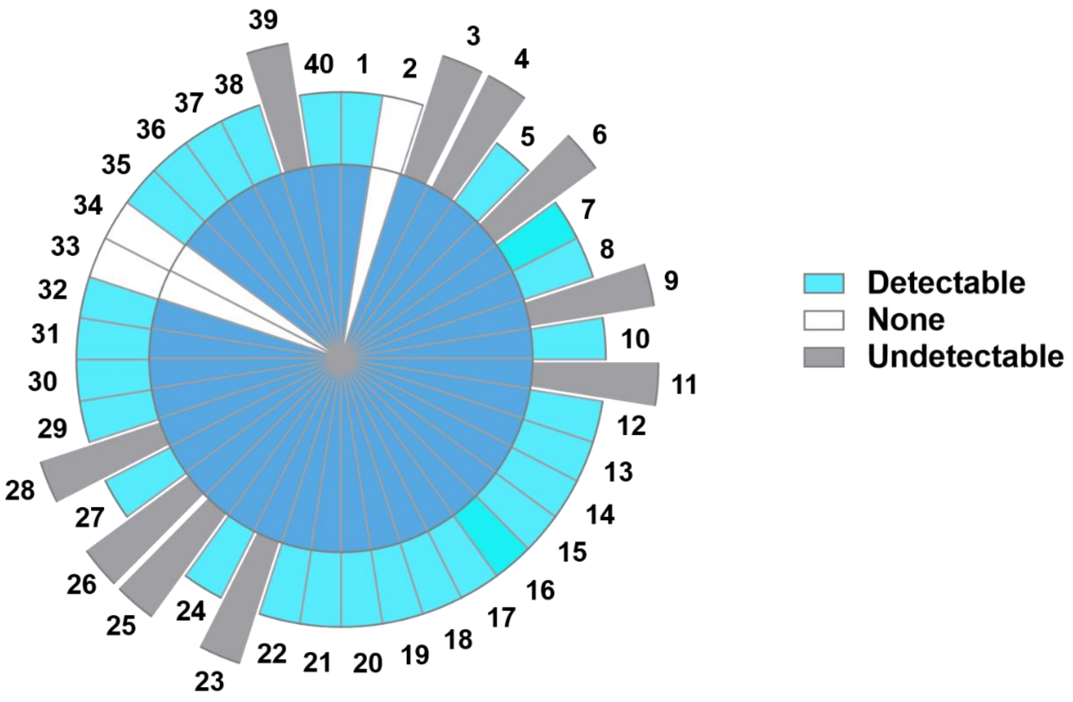

Fig. S6. Performance comparison in the library screening between GLMSD and the standard method based on UV-Vis absorption changes. The outer pie chart: UV method; and the inner pie chart: GLMSD. Cyan or Blue: successful hits; White: compds unavailable commercially; Grey: missing hits due to the "blind-zone" issue of the UV-Vis absorption method.

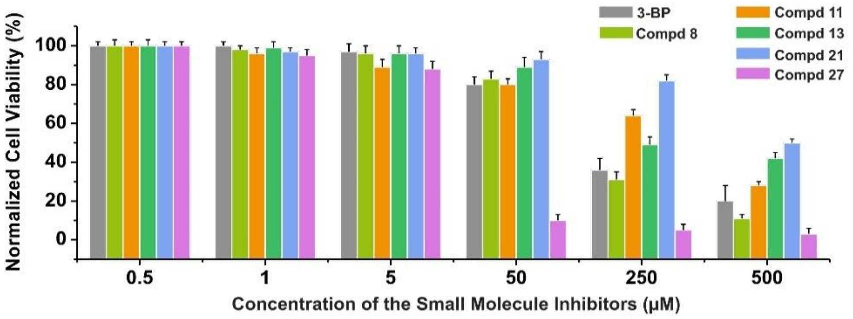

Fig. S7. Antiproliferation activity of 3-BP, Compd 8, Compd 11, Compd 13, Compd 21 and Compd 27 against brain glioma cell line U87. Data are representative of six independent experiments. (n=6)

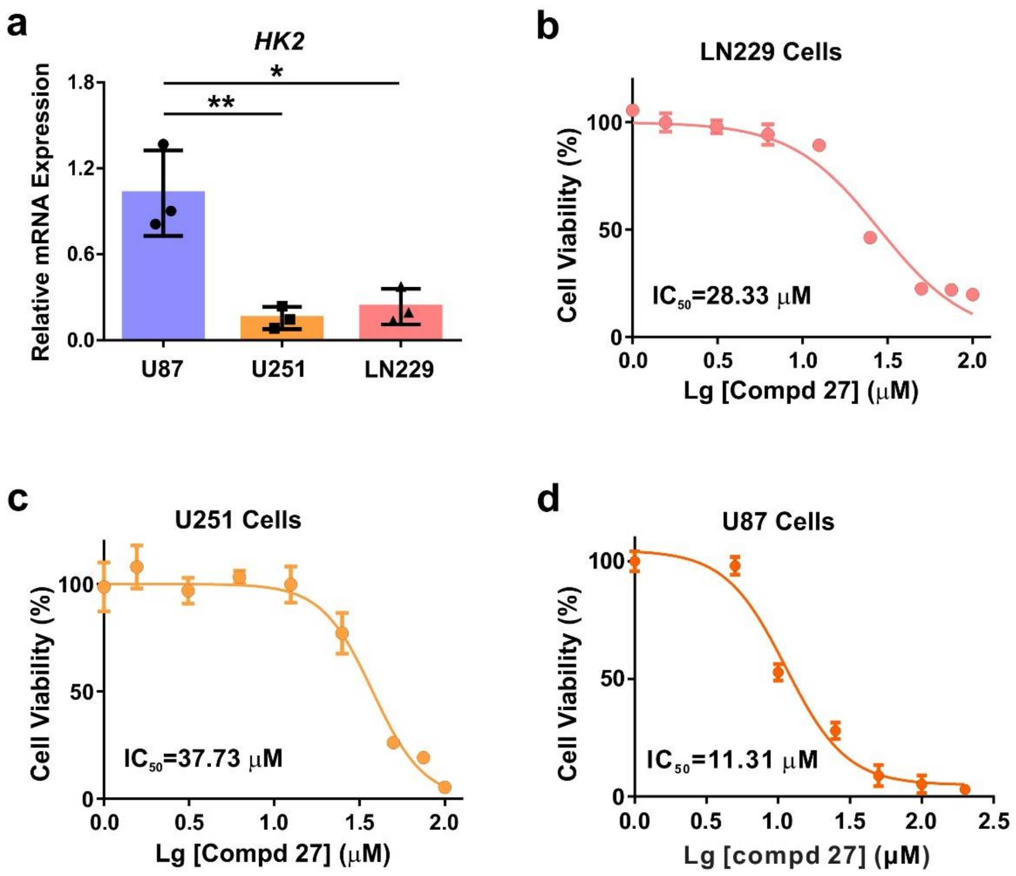

Fig. S8. Suppression of glioma cells by Compd 27 in a HK2-dependent manner. (a) quantitative RT-PCR analysis of HK2 in LN229 cells, U251 cells and U87 cells. Statistical significance was calculated using the unpaired two-tailed Student's t-test. *: $P < 0.05$; **: $P < 0.01$, (n=3). (b, c, d) IC_{50} values of Compd 27 on LN229, U251, and U87 cells. Repeated tests (n = 6). Cell treatment by Compd 27 for 24 h. Note: (d) was identical to Fig.2A, and presented here again for comparison.

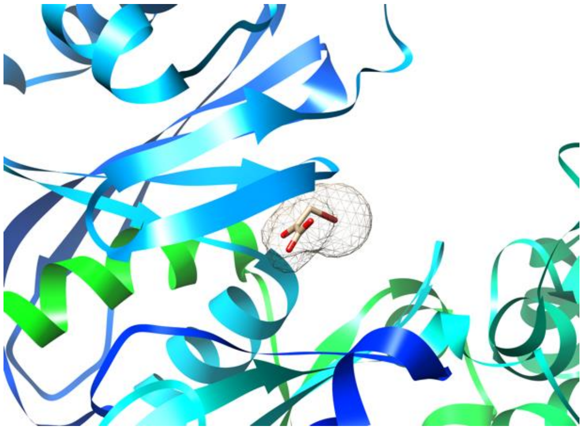

Fig. S9. Schematic representation of the binding structures of 3-BP-HK2 complex predicted by *Glide* XP docking simulations.

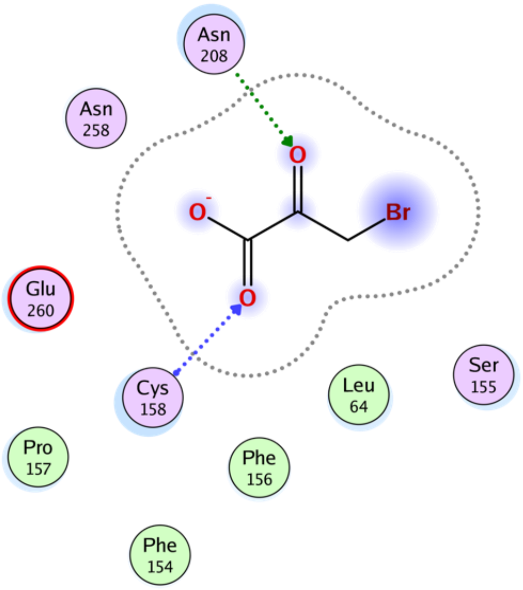

Fig. S10. 2-D schematic diagram of the binding patterns of 3-BP-HK2 complex highlighting the hydrogen bonds interacting with residues Asn208 and Cys158. Color codes: the pink (polar amino acids); the green (greasy residues).

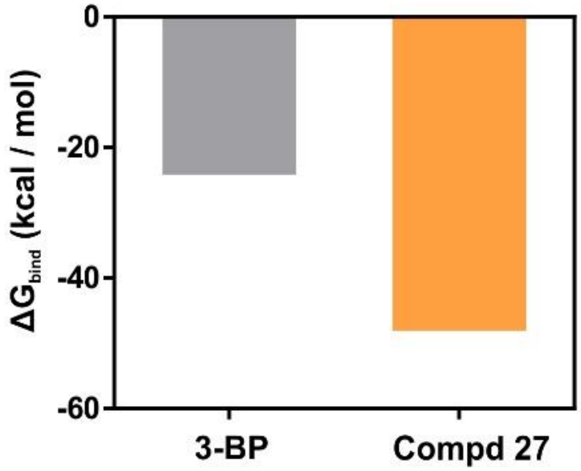

Fig. S11. The binding free energy (ΔG$_{bind}$) of Compd 27 or 3-BP for hexokinase-2, as a summary of the calculations of total interaction energy, including van der Waals and electrostatic contributions, as well as polar and non-polar desolvation contributions to ligand-receptor interactions calculated by *Prime MM-GBSA* module in Schrödinger.

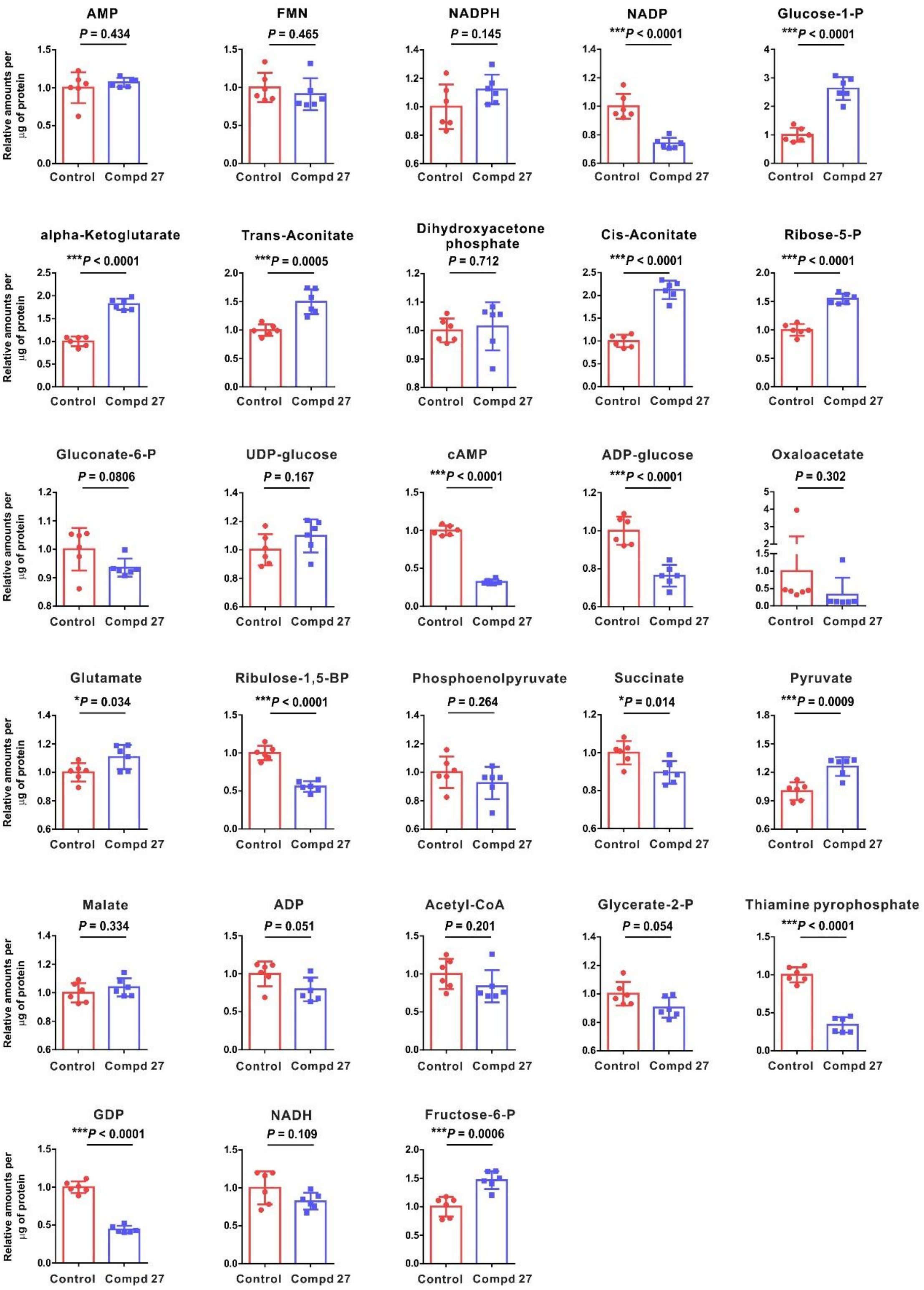

Fig. S12. Quantification of the changes in other intracellular metabolites of the energy metabolism pathways in U87 cells following treated with/without Compd 27. The number of biological repeats (n=6). Statistical significance was calculated using the unpaired two-tailed Student's *t*-test. *: $P < 0.05$; **: $P < 0.01$; ***: $P < 0.001$.

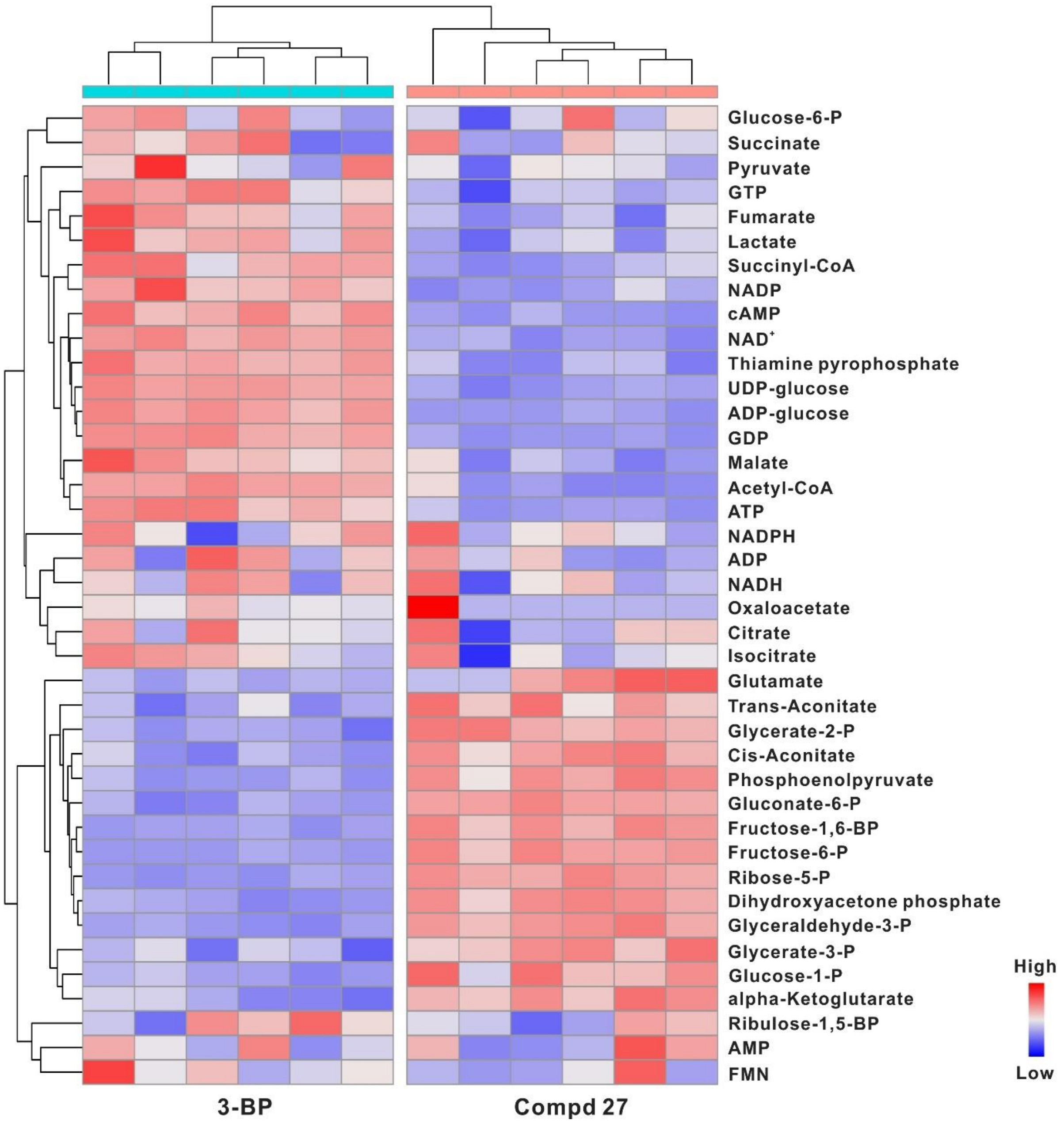

Fig. S13. Relative difference heatmap of the clustered metabolites in U87 cells treated with 3-BP or Compd 27. The number of biological repeats (n=6).

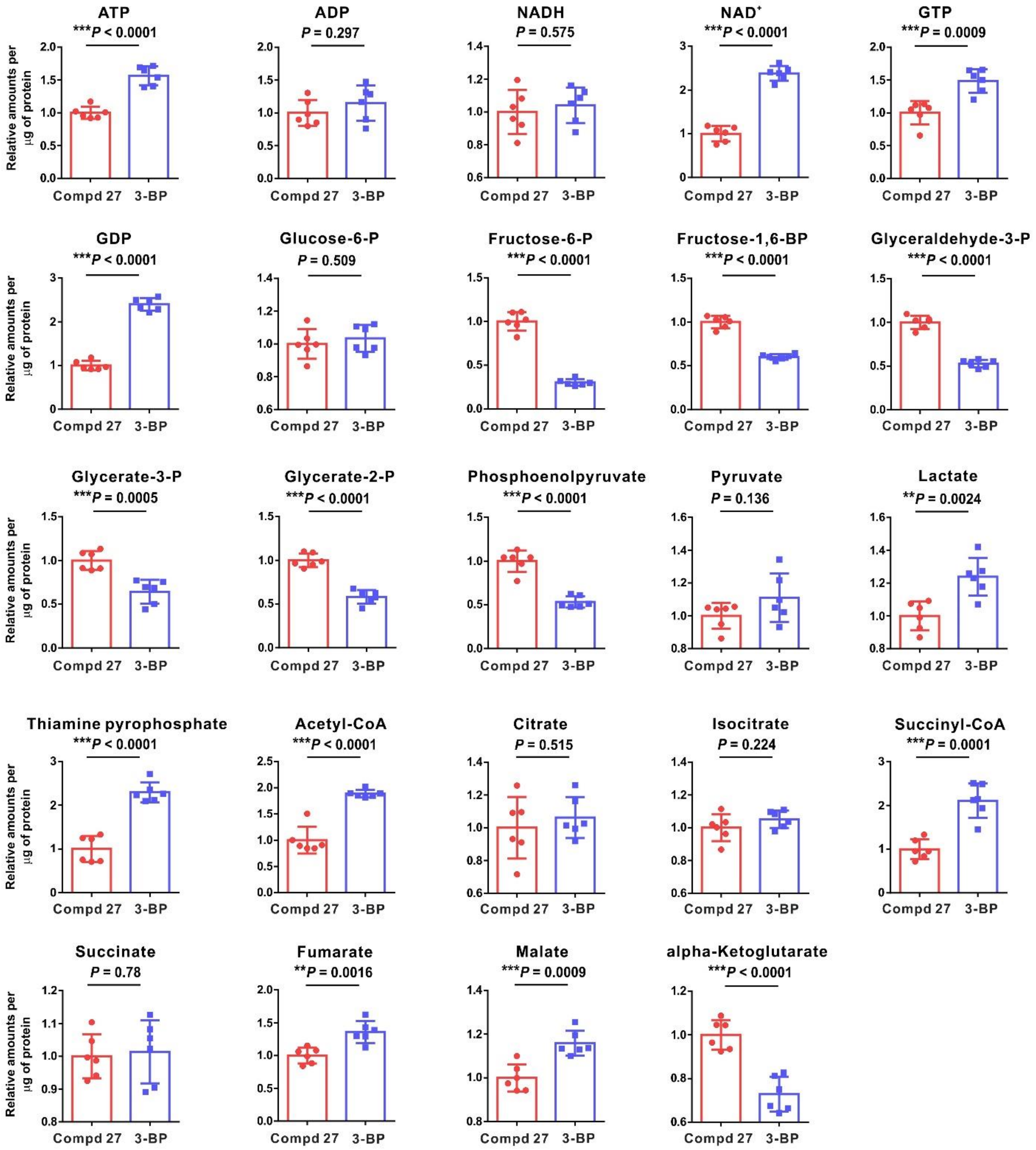

Fig. S14. Quantification of the changes in intracellular metabolites of the Glycolysis and TCA cycle pathways in U87 cells treated with 3-BP or Compd 27. The number of biological repeats (n=6). Statistical significance was calculated using the unpaired two-tailed Student's t-test. *: $P < 0.05$; **: $P < 0.01$; ***: $P < 0.001$.

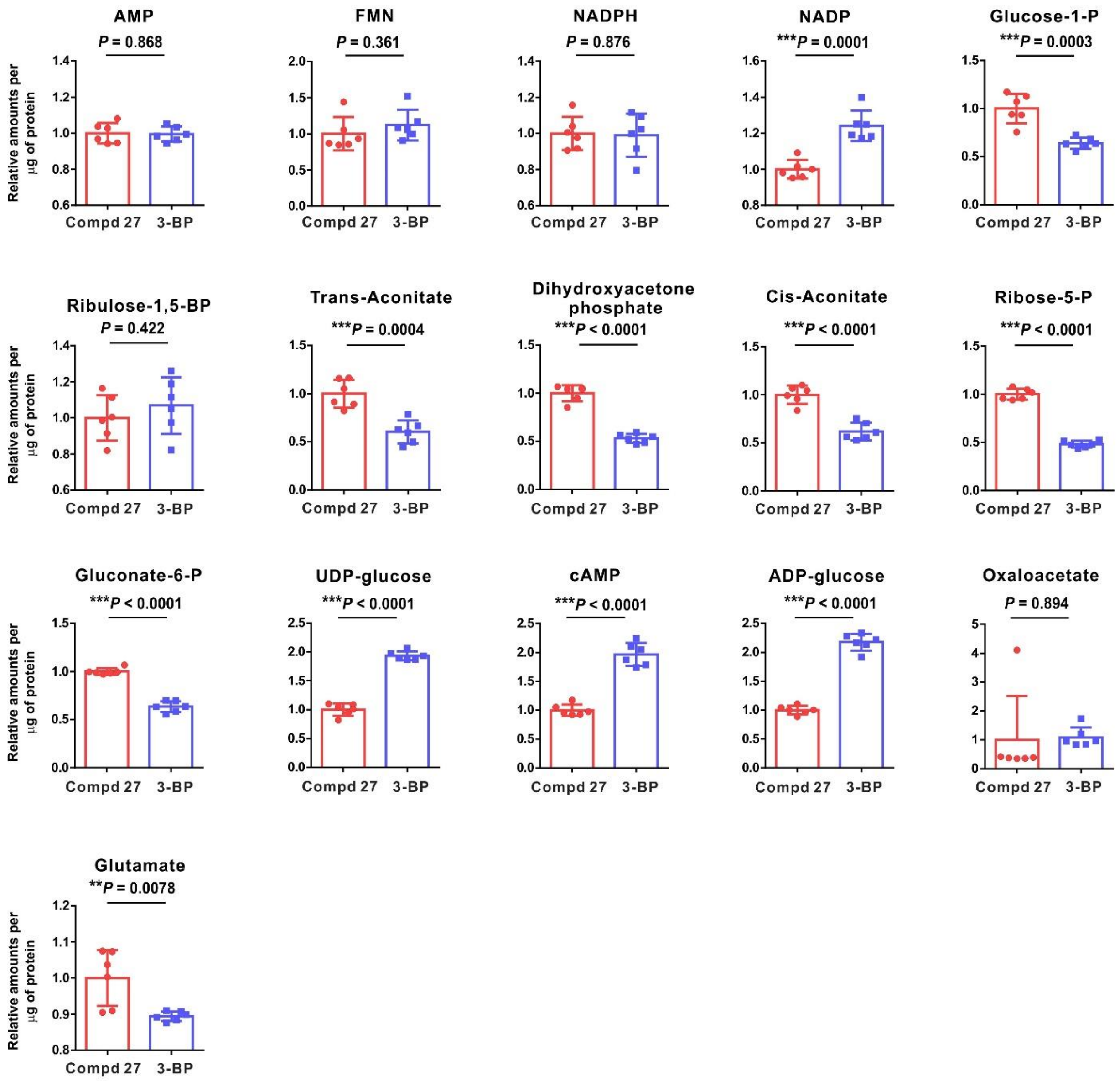

Fig. S15. Quantification of the changes in intracellular metabolites of the Gluconeogenesis and Pentose phosphate pathways in U87 cells treated with 3-BP or Compd 27. The number of biological repeats (n=6). Statistical significance was calculated using the unpaired two-tailed Student's *t*-test. *: $P < 0.05$; **: $P < 0.01$; ***: $P < 0.001$.

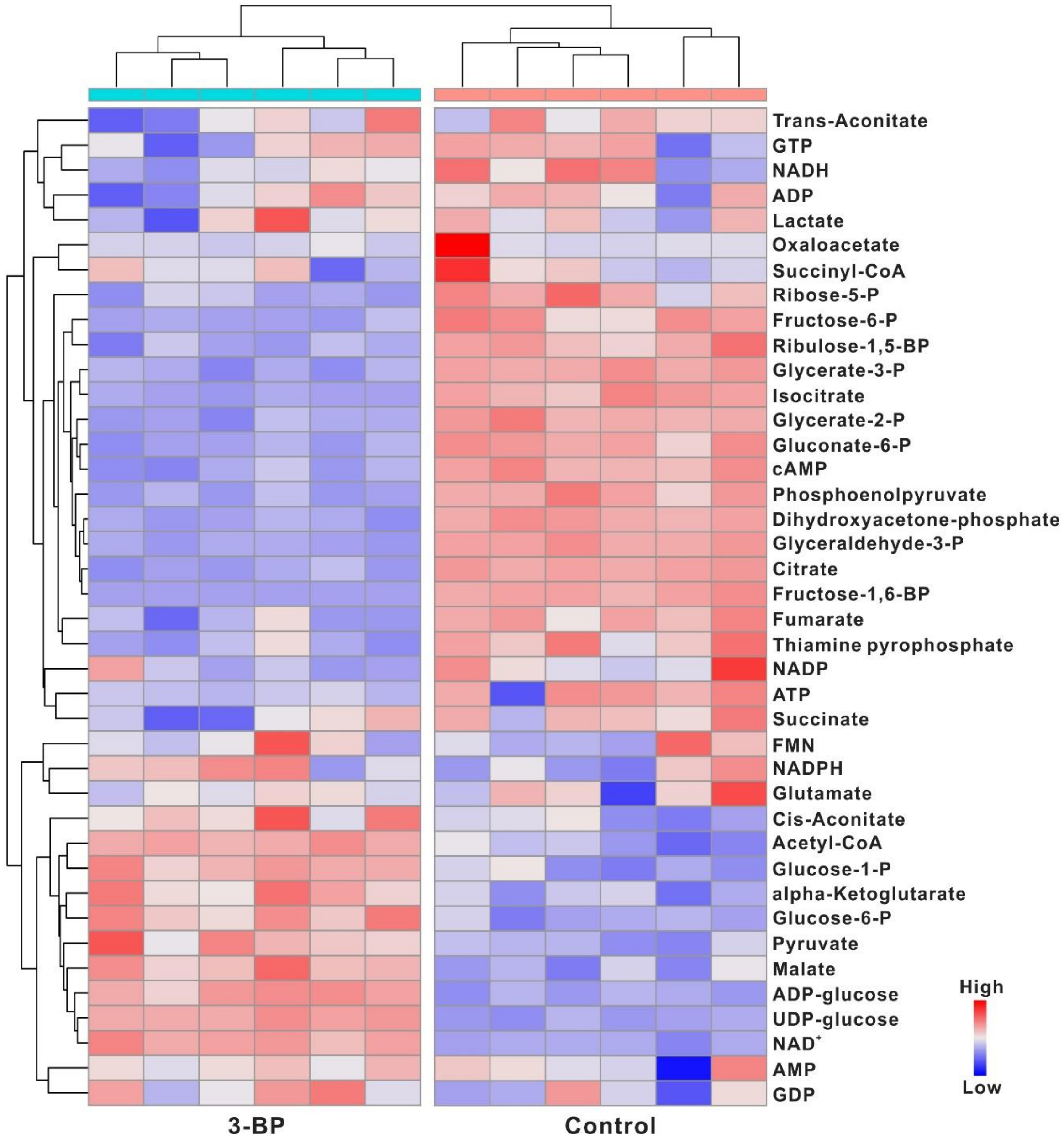

Fig. S16. Relative difference heatmap of the clustered metabolites in U87 cells treated with/without 3-BP. The number of biological repeats (n=6).

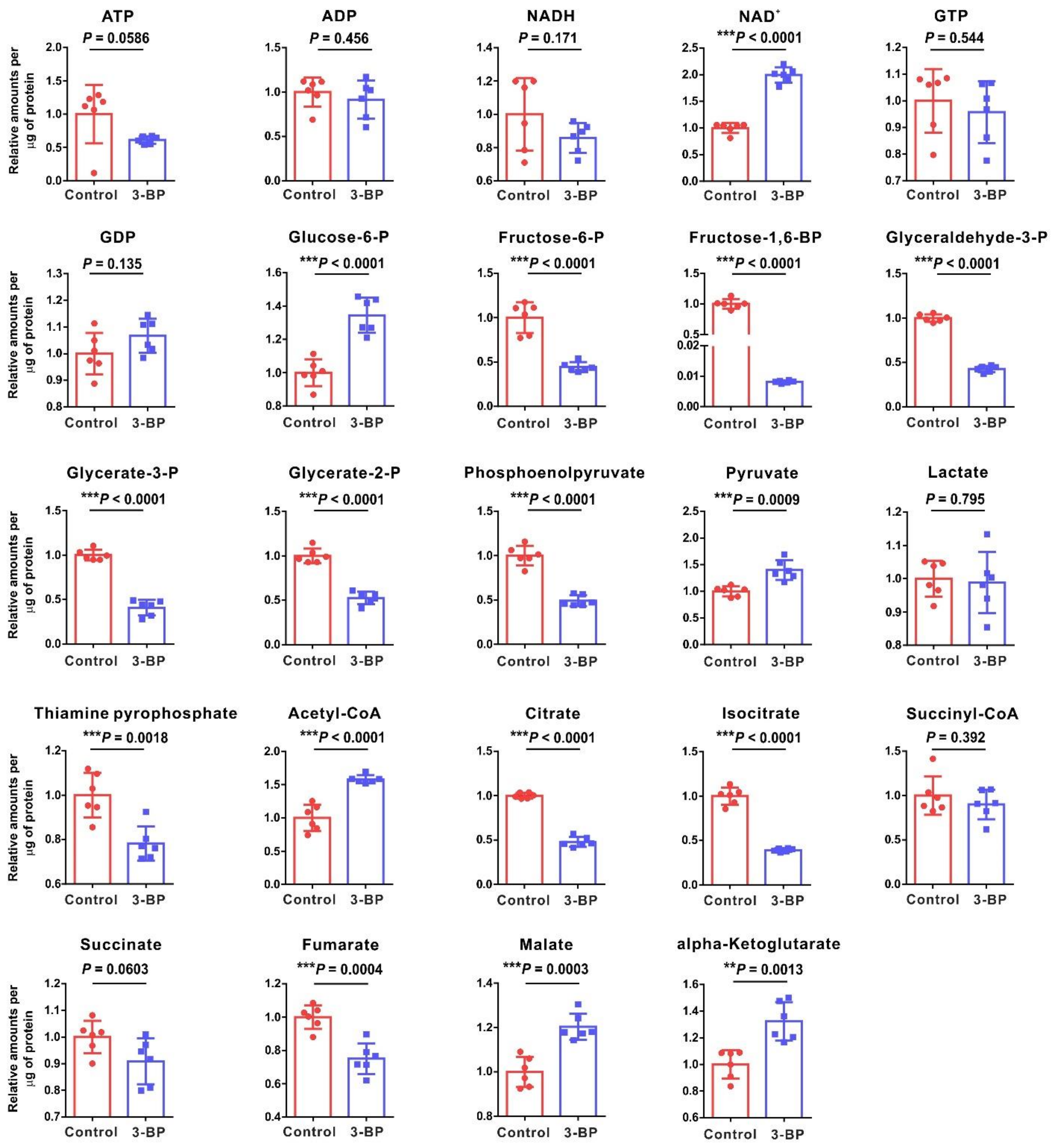

Fig. S17. Quantification of the changes in intracellular metabolites of the Glycolysis and TCA cycle pathways in U87 cells treated with/without 3-BP. The number of biological repeats (n=6). Statistical significance was calculated using the unpaired two-tailed Student's t-test. *: P < 0.05; **: P < 0.01; ***: P < 0.001.

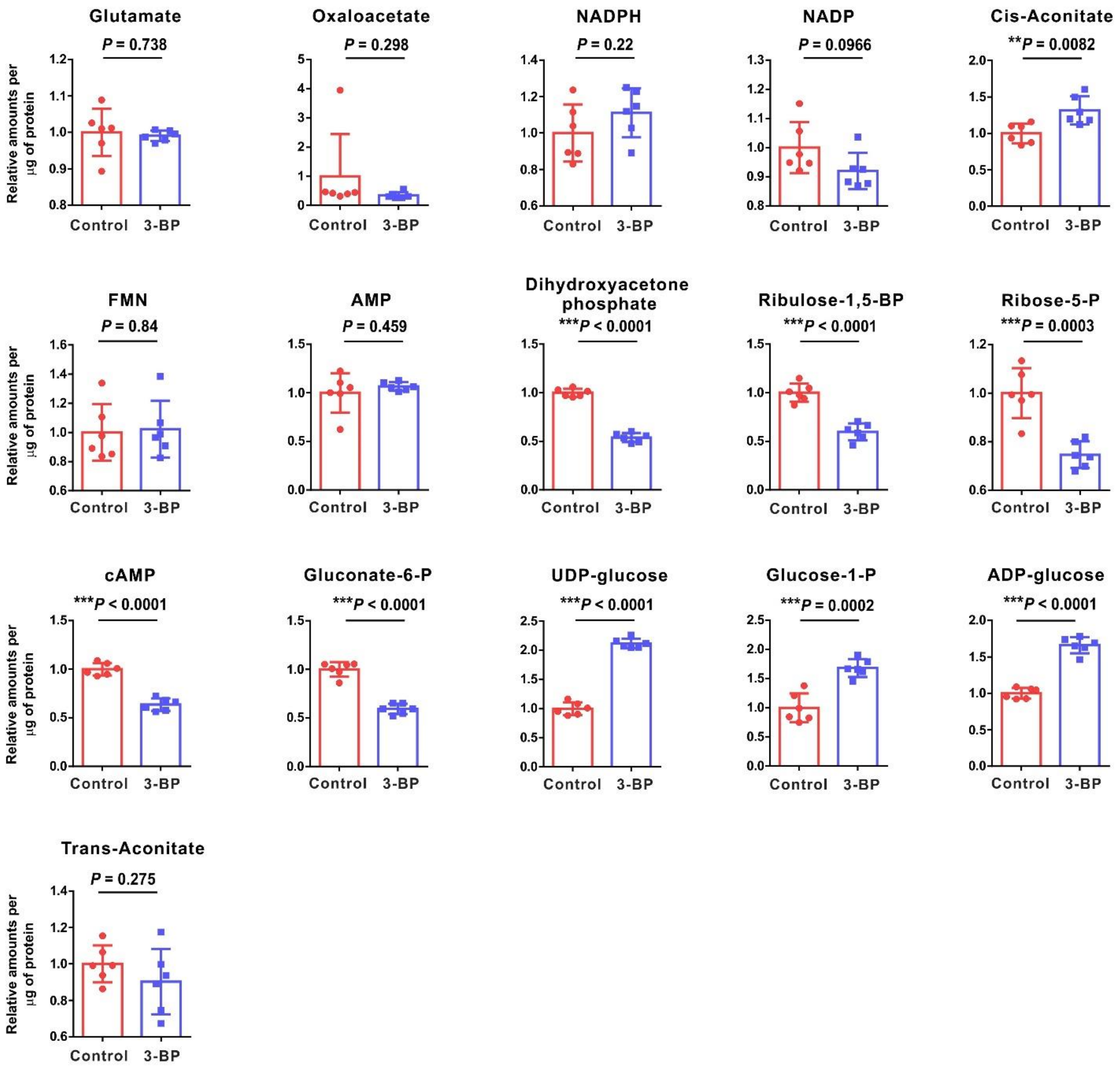

Fig. S18. **Quantification of the changes in intracellular metabolites of the Gluconeogenesis and Pentose phosphate pathways in U87 cells treated with/without 3-BP.** The number of biological repeats (n=6). Statistical significance was calculated using the unpaired two-tailed Student's t-test. *: $P < 0.05$; **: $P < 0.01$; ***: $P < 0.001$.

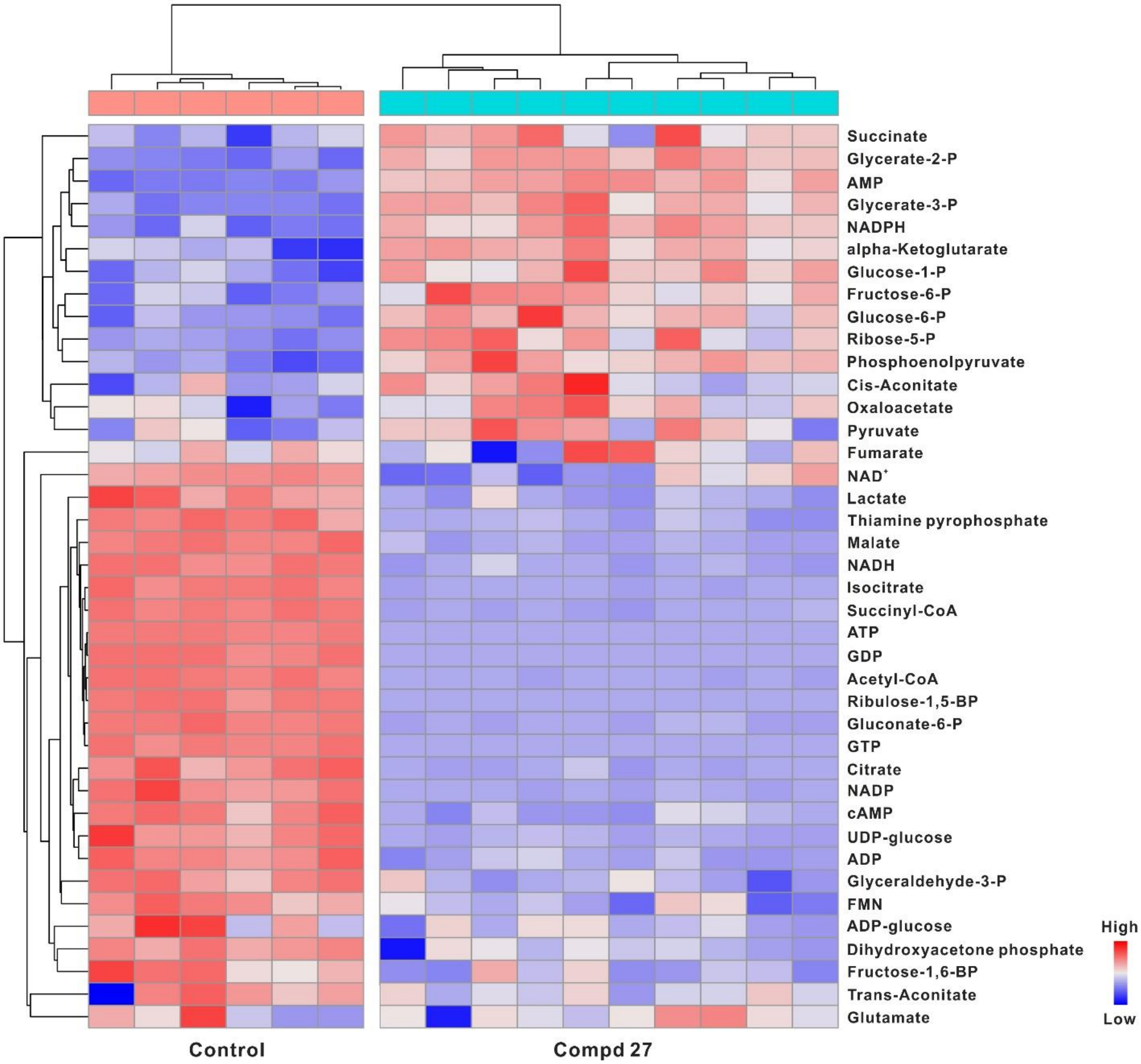

Fig. S19. Relative difference heatmap of the clustered metabolites in subcutaneous U87MG tumors treated with Compd 27 or PBS. The number of biological repeats (n=6) for the PBS control and (n= 10) for Compd 27.

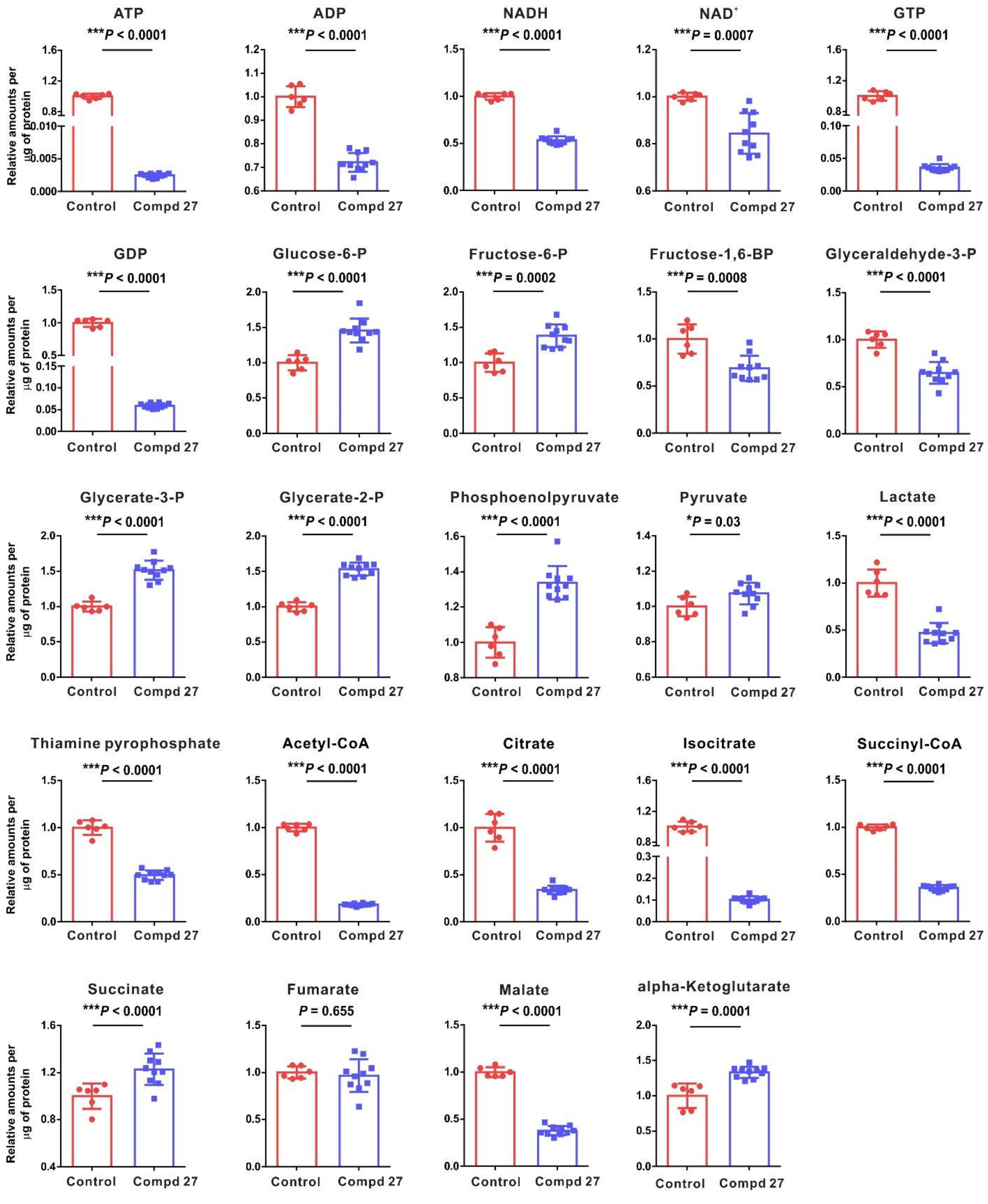

Fig. S20. Quantification of the changes of the metabolites in the Glycolysis and TCA cycle pathways. Subcutaneous U87MG tumors treated with Compd 27 or PBS. The number of biological repeats (n=6) for the PBS control and (n= 10) for Compd 27. Statistical significance was calculated using the unpaired two-tailed Student’s t-test. *: P < 0.05; **: P < 0.01; ***: P < 0.001.

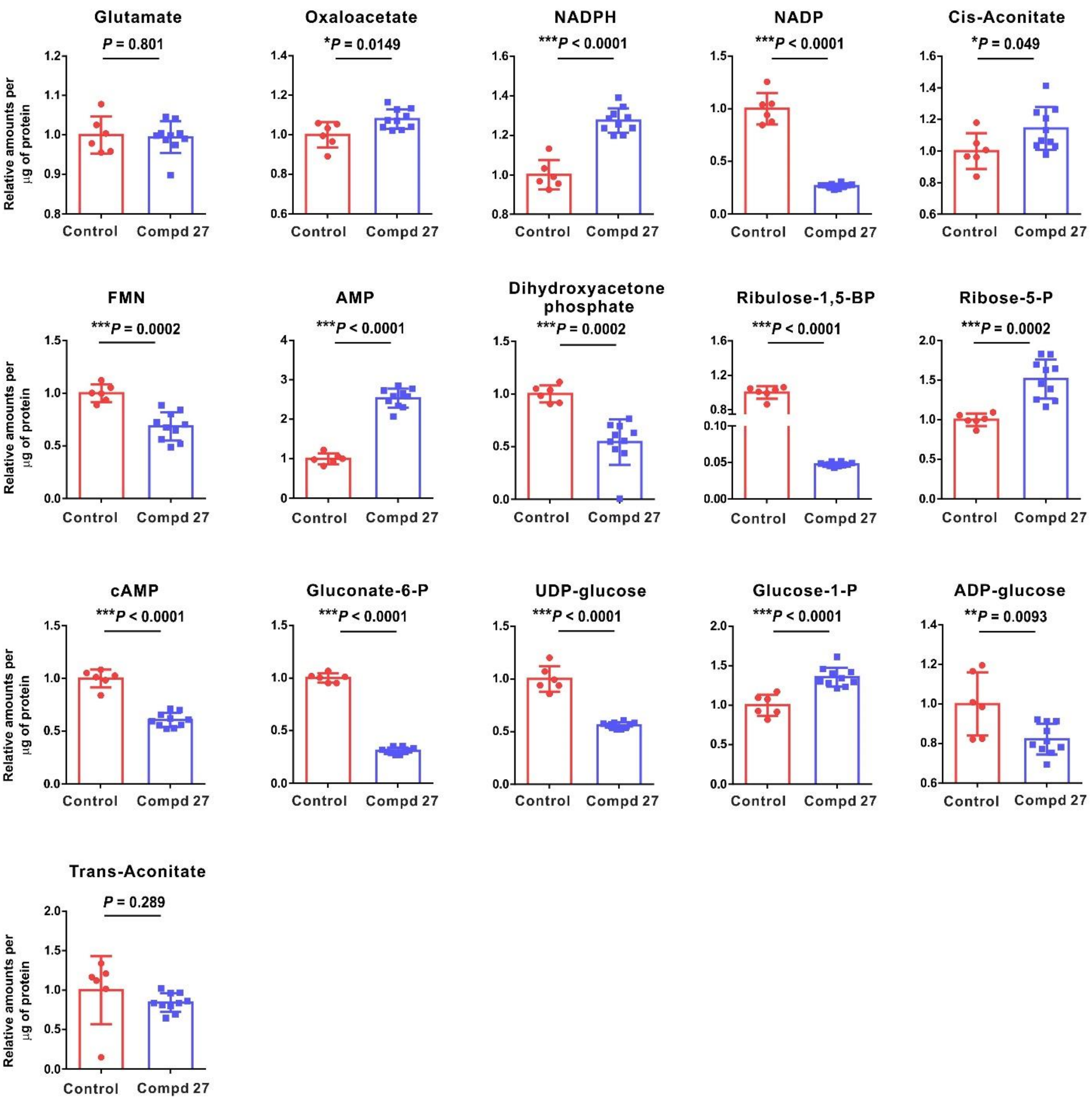

Fig. S21. Quantification of the changes of the metabolites in the Gluconeogenesis and Pentose phosphate pathways. Subcutaneous U87MG tumors treated with Compd 27 or PBS. The number of biological repeats (n=6) for the PBS control and (n= 10) for Compd 27. Statistical significance was calculated using the unpaired two-tailed Student's *t*-test. *: $P < 0.05$; **: $P < 0.01$; ***: $P < 0.001$.

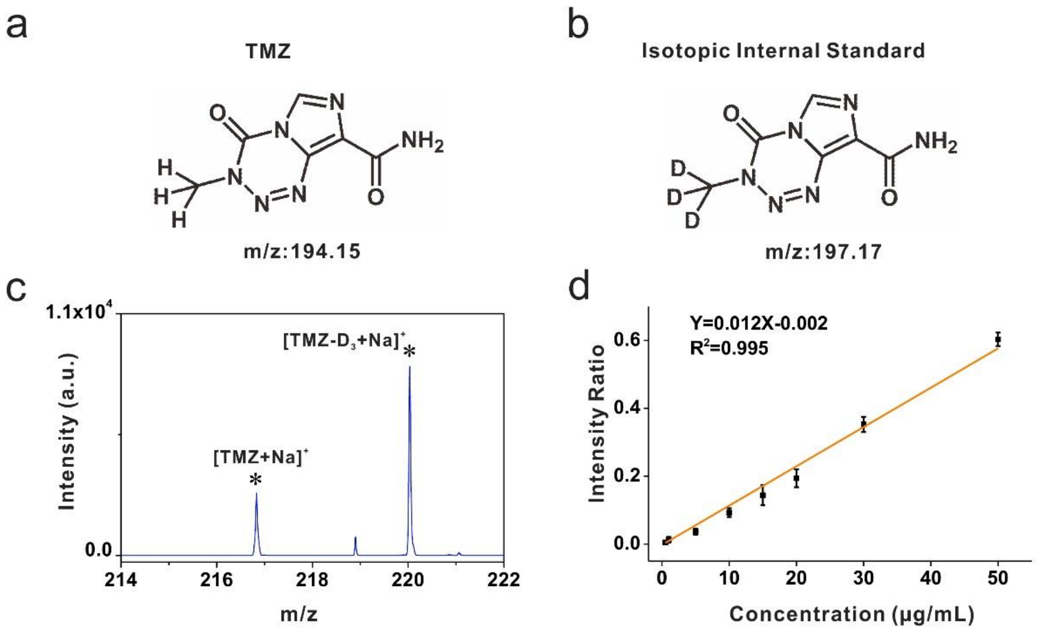

Fig. S22. Characteristic of quantitative analysis for TMZ. (a) Chemical structure of TMZ. (b) Chemical structure of isotopic internal standard. (c) Mass spectrum of the TMZ ($[M+Na]^+$, m/z : 216.15) and isotopic internal standard ($[M+Na]^+$, m/z : 219.17) in positive-ion mode. (d) Calibration curves for quantitative analysis of TMZ. TMZ-D_3 was used as the internal standard: 10 μg/mL.

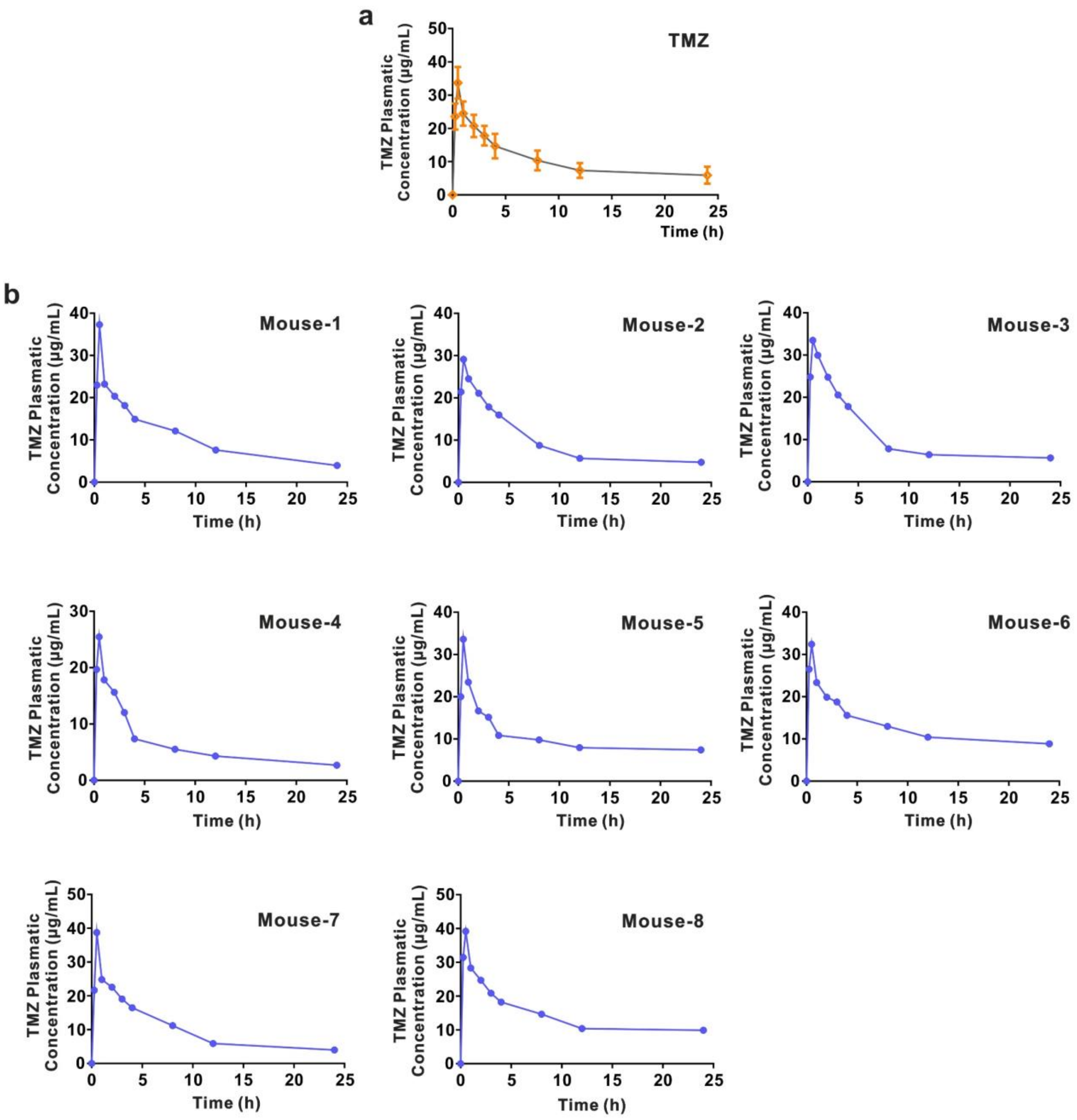

Fig. S23. Single dose pharmacokinetic profiles of TMZ. (a) The mean concentration–time profiles of TMZ in plasma following intraperitoneal administration in mice (TMZ: 40 mg/kg, n=8). (b) The individual TMZ concentration–time profiles of each mouse. The number of spectral repeats (sample spots, n=3).

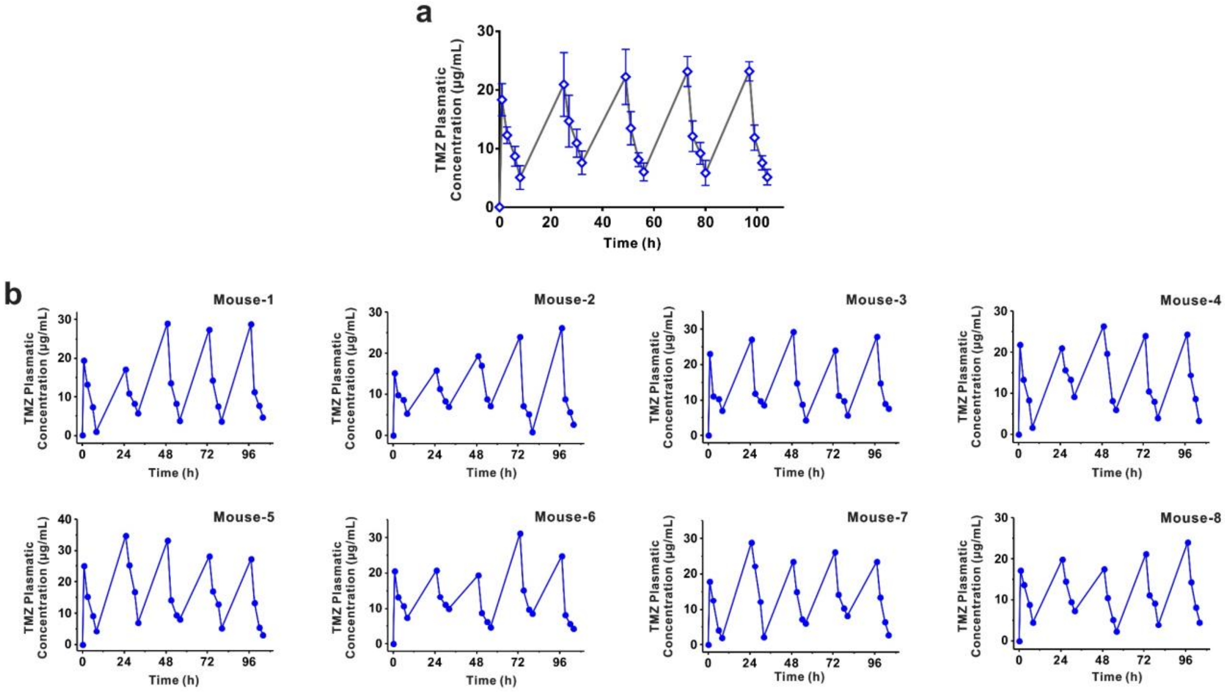

Fig. S24. Daily dose pharmacokinetic profiles of TMZ. (a) Daily dose pharmacokinetic curve detection using GLMSD. The mice were administered with TMZ (dosage: 40 mg/kg) daily for 5 days. The samples were taken at the indicated time (1, 3, 6, and 8 h) after the last dose. The number of biological repeats (mice, n = 8). (**b**) The pharmacokinetic curves of each mouse. The number of spectral repeats (sample spots, n=3)

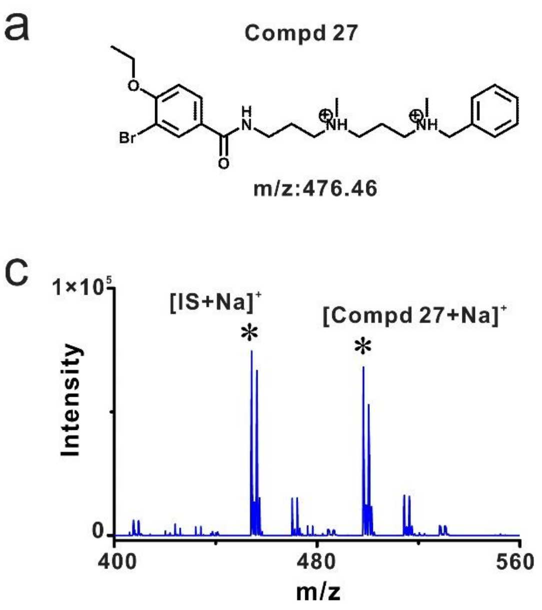

Fig. S25. Characteristic of quantitative analysis for Compd 27. (a) Chemical structure of Compd 27. (b) Chemical structure of internal standard. (c) Mass spectrum of the Compd 27 ($[M+Na]^+$, m/z: 499.46) and internal standard ($[M+Na]^+$, m/z: 455.41) in positive-ion mode. (d) Calibration curves for quantitative analysis of Compd 27. Internal standard as the internal standard: 10 μg/mL.

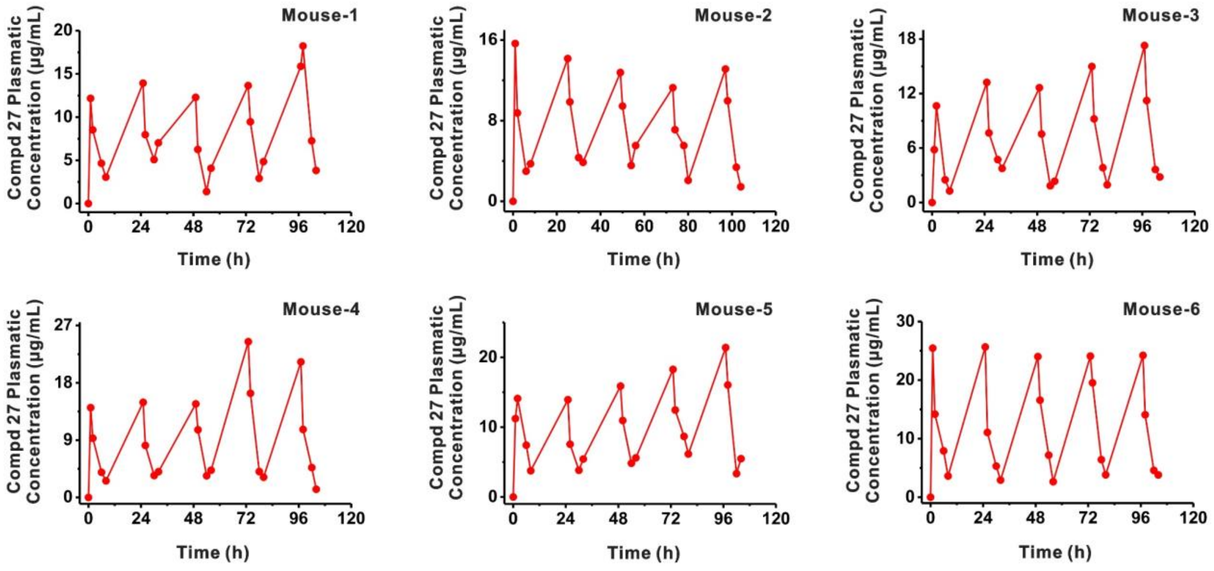

Fig. S26. Daily dose pharmacokinetic curve for each mouse. The mice were administered Compd 27 daily for 5 days and the dose was 40 mg/kg. The samples were taken at the indicated time (1, 3, 6, and 8 h) after the last dose. The number of spectral repeats (sample spots, n=3)

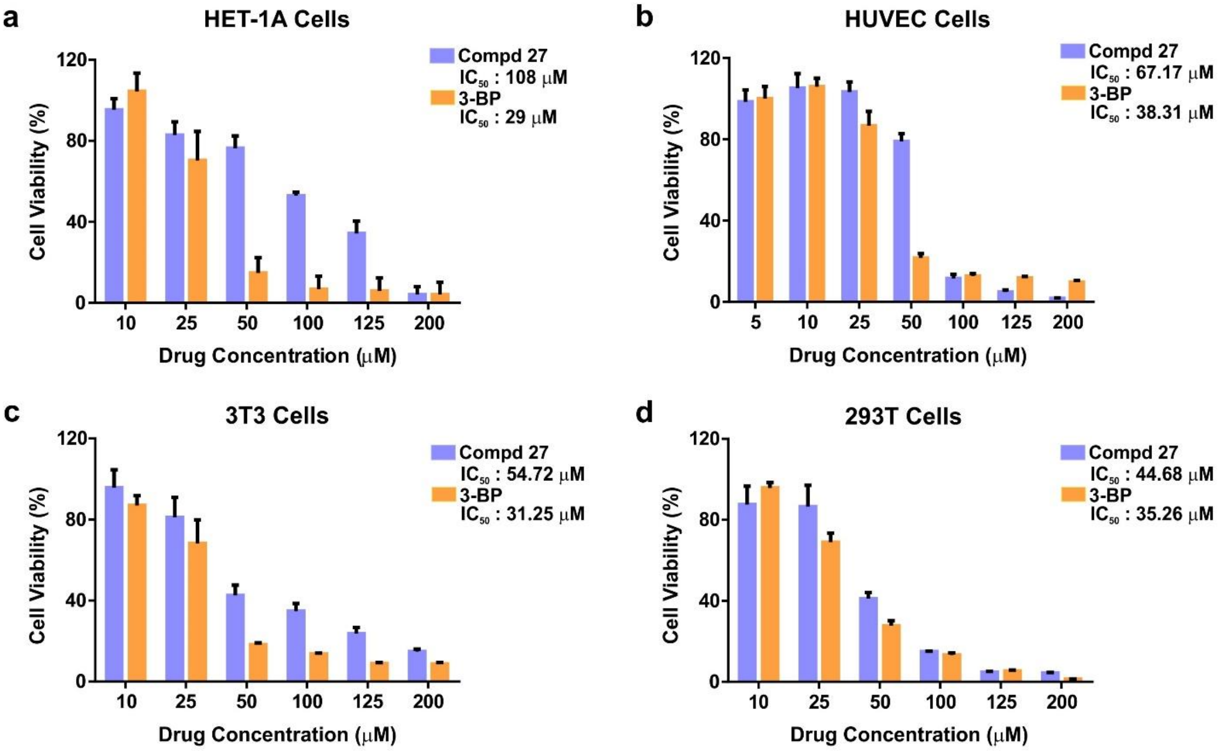

Fig. S27. Relative cell viabilities of HET-1A, HUVEC cells, 3T3 cells, and 293T cells after treatment with Compd 27 or 3-BP for 24 h. Error bar: standard deviation (n = 6).

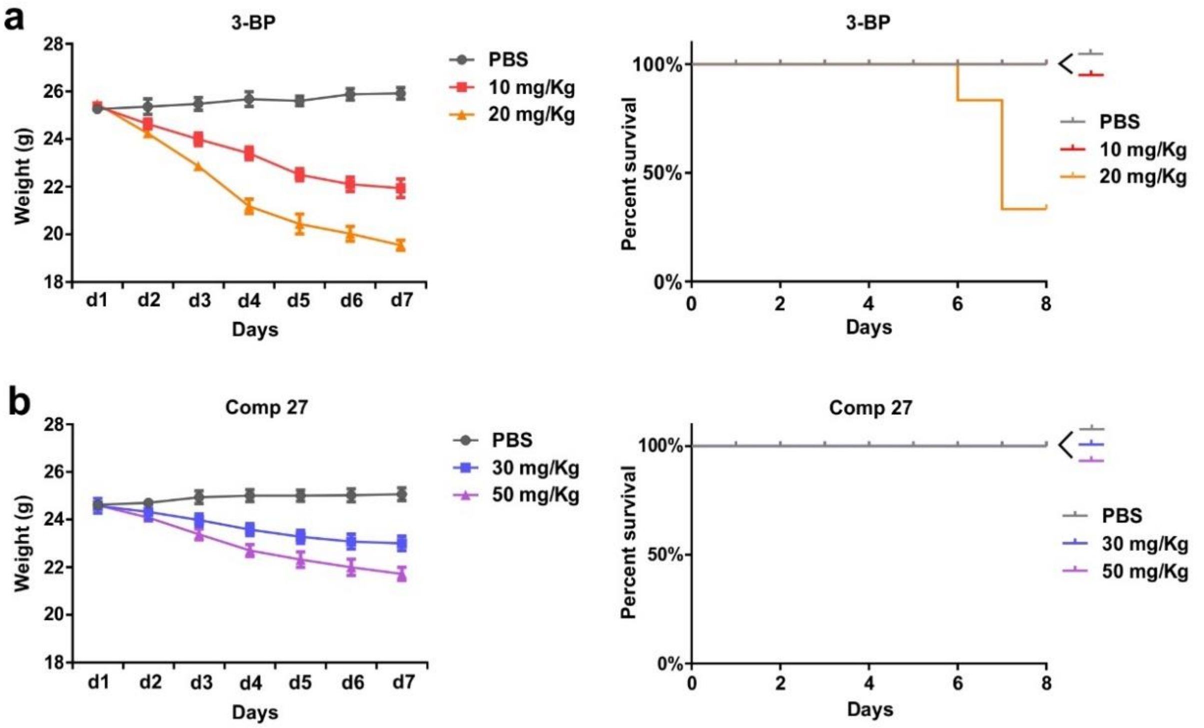

Fig. S28. Body weights and survival rates of mice intraperitoneally injected with Compd 27 (n = 5) or 3-BP (n = 5).

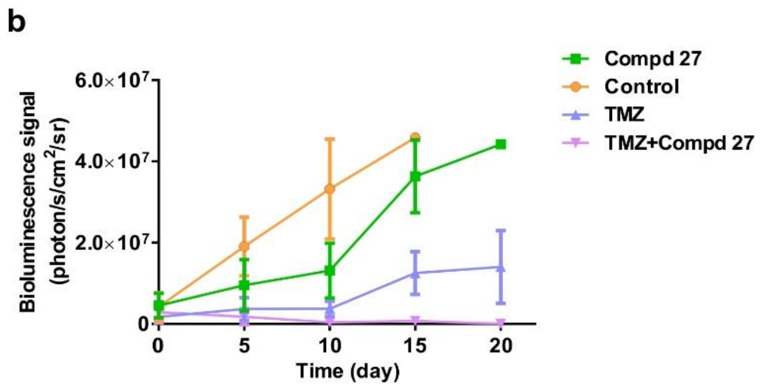

Fig. S29. Average quantitative bioluminescence signals of tumors in control and treated groups. Data are representative of seven independent experiments. (n=7)

Table S1. The signal-to-noise ratio of three carbohydrates in in four high-salt buffers by using four matrices.

	NaCl (500 mM)	KCl (250 mM)	Tris-HCl (250 mM)	PBS (500 mM)	
GDs	142	9	210	242	Glucose
CHCA	0	0	0	0	
DHB	0	0	0	0	
SA	13	0	0	17	
GDs	2051	163	760	5407	Maltose
CHCA	0	15	0	6	
DHB	22	16	0	356	
SA	6	0	0	48	
GDs	158	151	2005	1406	Maltohexaose
CHCA	41	0	0	65	
DHB	17	11	182	274	
SA	21	0	23	126	

Table S2. Compound information for all the synthesized inhibitors.

Index	Molecular Weight	Structure	docking score	H_Acceptors	H_Donors	Log D	Log P	Lipinski rule of five _ violation	Opera rules_ violation
Compd 1	336.34	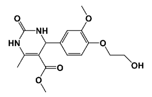	-7.193	6	4	1.560	1.560	0	0
Compd 2	543.71	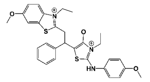	-8.063	3	2	2.890	3.640	0	0
Compd 3	340.41	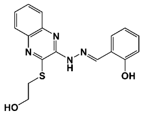	-7.435	6	5	1.040	1.040	0	0
Compd 4	403.44	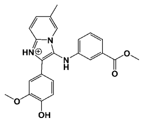	-7.479	3	1	2.660	5.130	0	0

Compd 5	348.36	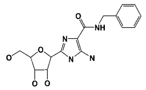	-7.457	7	3	1.650	1.660	0	0
Compd 6	305.34	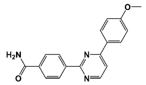	-7.155	4	1	2.840	2.840	0	0
Compd 7	398.27	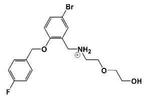	-7.494	4	0	5.455	5.421	2	3
Compd 8	369.44	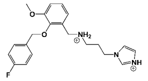	-7.241	5	2	2.210	2.210	0	1
Compd 9	383.49	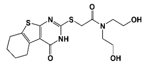	-7.971	9	6	1.400	1.400	1	2

Compd 10	546.58	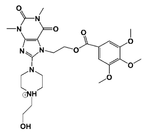	-7.238	7	2	1.070	3.470	0	0
Compd 11	381.4	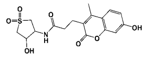	-7.870	6	1	5.351	5.346	2	3
Compd 12	422.43	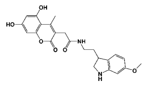	-7.329	6	3	0.540	0.540	0	0
Compd 13	244.29	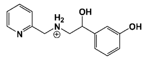	-7.329	8	4	0.600	0.600	1	3
Compd 14	364.45	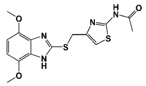	-7.162	4	1	4.590	4.590	1	2

Compd 15	366.27	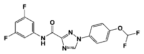	-7.272	3	1	2.900	2.900	0	0
Compd 16	328.48	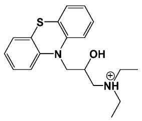	-7.955	3	2	2.520	3.560	0	0
Compd 17	493.55	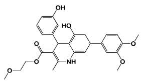	-7.885	3	1	1.210	3.060	0	0
Compd 18	524.43	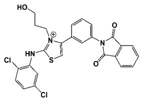	-7.472	3	3	1.060	3.180	0	0
Compd 19	326.39	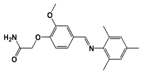	-7.505	3	3	0.400	0.600	0	0

Compd 20	269.3	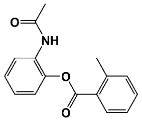	-7.138	3	1	3.020	3.020	0	0
Compd 21	488.49	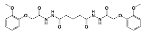	-7.182	5	3	4.390	4.430	0	0
Compd 22	358.46	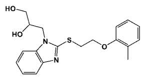	-8.060	4	4	3.360	3.630	0	0
Compd 23	367.28	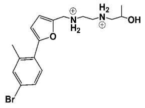	-7.741	6	3	-0.040	-0.010	0	0
Compd 24	426.51	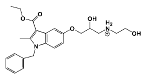	-7.272	5	2	2.670	2.680	0	0

Compd 25	316.4	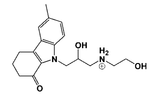	-7.328	11	1	0.980	1.310	2	3
Compd 26	353.44	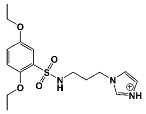	-7.472	4	1	2.350	4.690	0	2
Compd 27	476.46	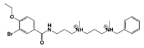	-7.361	7	2	3.060	3.060	0	1
Compd 28	295.76	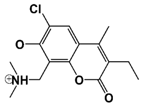	-8.147	6	3	1.850	3.360	0	1
Compd 29	399.4	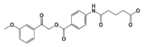	-7.162	5	3	0.360	2.080	0	0

Compd 30	461.57	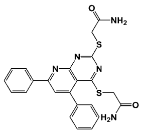	-7.386	5	1	2.140	2.450	0	0
Compd 31	481.47	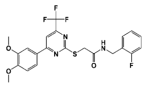	-7.992	5	1	3.850	3.870	0	0
Compd 32	291.35	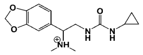	-7.145	2	1	2.000	4.050	0	0
Compd 33	414.41	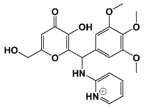	-7.536	6	3	1.930	2.080	0	1
Compd 34	387.46	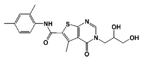	-7.479	4	2	3.860	3.860	0	0

Compd 35	300.31	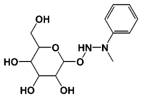	-7.436	7	3	0.830	0.830	0	0
Compd 36	251.35	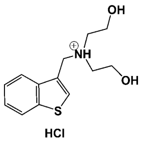	-7.611	4	2	0.790	1.370	0	0
Compd 37	226.18	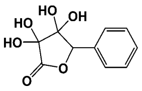	-7.377	5	2	4.100	4.250	0	0
Compd 38	411.52	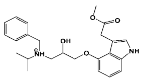	-8.662	5	2	3.160	4.059	0	0
Compd 39	368.51	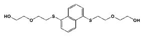	-8.507	4	2	2.390	2.390	0	1

Compd 40	353.84	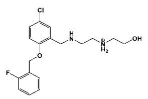	-8.203	3	3	0.639	2.829	0	0
