# Supplementary material for: High-throughput glycolytic inhibitor discovery targeting glioblastoma by graphite dots–assisted LDI mass spectrometry
Source: Sci Adv. 2022 Feb 16;8(7):eabl4923. doi: 10.1126/sciadv.abl4923 (PMC10921956; doi:10.1126/sciadv.abl4923)
Supplement: Supplementary file 2 — Data files S1 and S2 [file sciadv.abl4923_data_files_s1_and_s2.zip › sciadv.abl4923_data_file_s1.pdf]

Supplementary Data S1

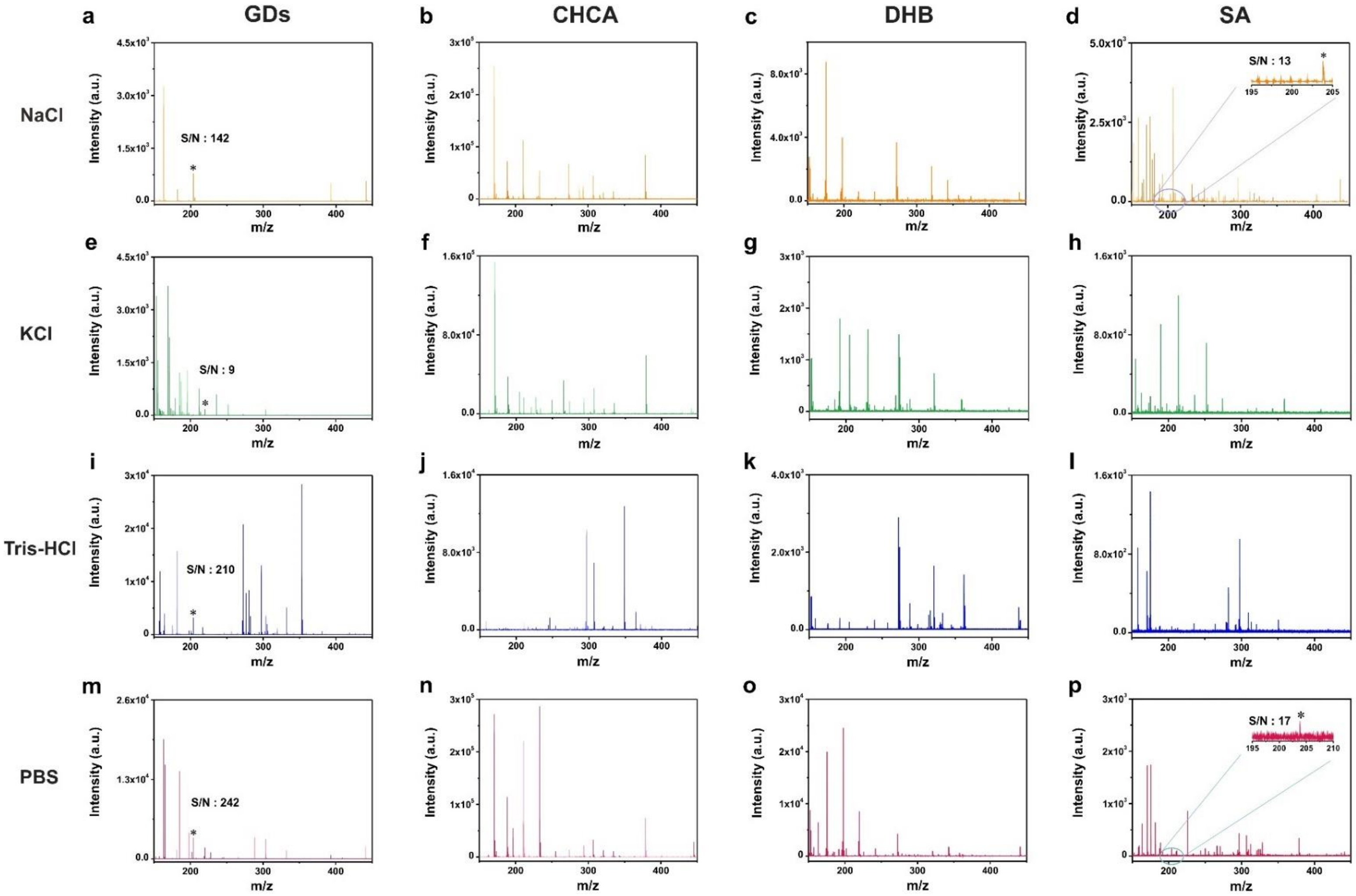

The mass spectra of **glucose** dissolved in four high-salt buffers (NaCl, KCl, Tris-HCl and PBS). (a, e, i, m) GDs as the matrix to perform the MS analysis in four high-salt buffers. (b, f, j, n) CHCA as the matrix to perform the MS analysis in four high-salt buffers. (c, g, k, o) DHB as the matrix to perform the MS analysis in four high-salt buffers. (d, h, i, p) SA as the matrix to perform the MS analysis in four high-salt buffers. The reflector positive ion mode was employed and the $[M+Na]^+$ was defined as the target mass spectrum peak. (NaCl: 500 mM, KCl: 250 mM, Tris-HCl: 250 mM, PBS: 500 mM, glucose: 100 pmol).

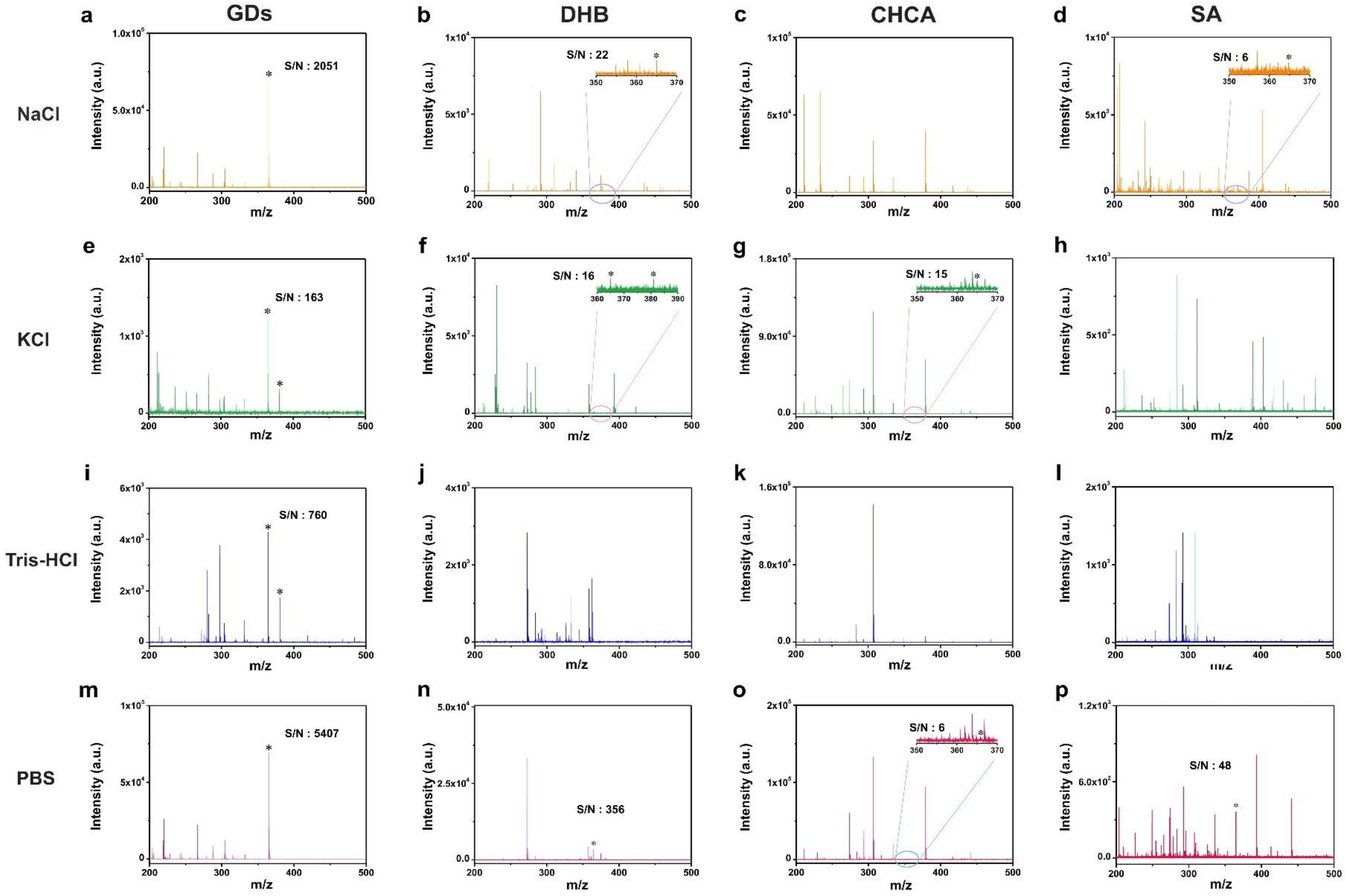
GDs
DHB
CHCA
SA
NaCl
KCl
Tris-HCl
PBS
S/N : 2051
S/N : 22
S/N : 6
S/N : 163
S/N : 16
S/N : 15
S/N : 760
S/N : 5407
S/N : 356
S/N : 6
S/N : 48
Intensity (a.u.)
m/z

The mass spectrum of **maltose** dissolved in four high-salt buffers (NaCl, KCl, Tris-HCl and PBS). (a, e, i, m) GDs as the matrix to perform the MS analysis in four high-salt buffers. (b, f, j, n) CHCA as the matrix to perform the MS analysis in four high-salt buffers. (c, g, k, o) DHB as the matrix to perform the MS analysis in four high-salt buffers. (d, h, i, p) SA as the matrix to perform the MS analysis in four high-salt buffers. The reflector positive ion mode was employed and the $[M+Na]^+$ was defined as the target mass spectrum peak. (NaCl: 500 mM, KCl: 250 mM, Tris-HCl: 250 mM, PBS: 500 mM, maltose: 100 pmol).

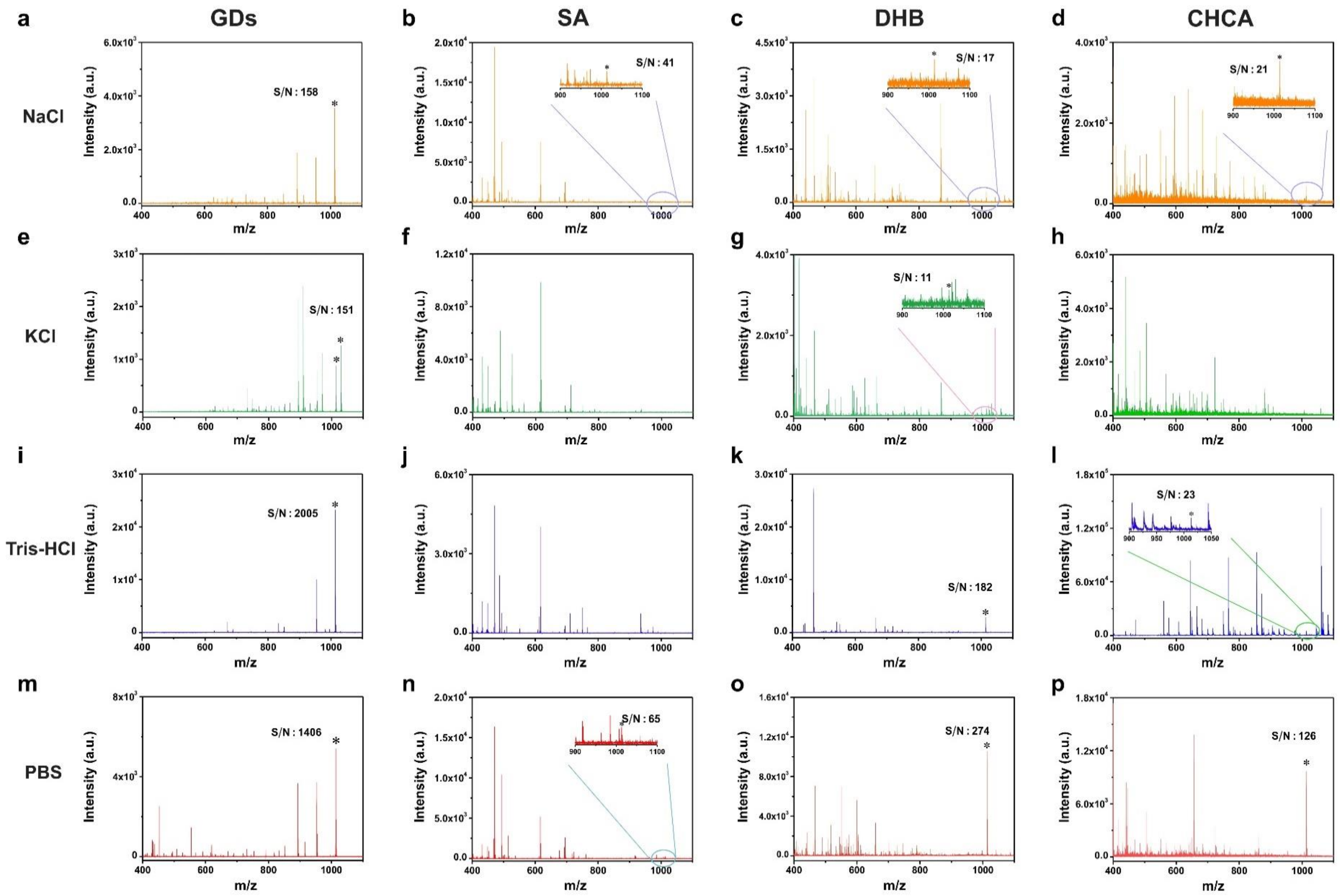

a
GDs
b
SA
c
DHB
d
CHCA
NaCl
KCl
Tris-HCl
PBS
S/N : 158
S/N : 41
S/N : 17
S/N : 21
e
f
g
h
S/N : 151
S/N : 11
i
j
k
l
S/N : 2005
S/N : 182
S/N : 23
m
n
o
p
S/N : 1406
S/N : 65
S/N : 274
S/N : 126
Intensity (a.u.)
m/z

The mass spectrum of **maltohexaose** dissolved in four high-salt buffers (NaCl, KCl, Tris-HCl and PBS). (a, e, i, m) GDs as the matrix to perform the MS analysis in four high-salt buffers. (b, f, j, n) CHCA as the matrix to perform the MS analysis in four high-salt buffers. (c, g, k, o) DHB as the matrix to perform the MS analysis in four high-salt buffers. (d, h, i, p) SA as the matrix to perform the MS analysis in four high-salt buffers. The reflector positive ion mode was employed and the $[M+Na]^+$ was defined as the target mass spectrum peak. (NaCl: 500 mM, KCl: 250 mM, Tris-HCl: 250 mM, PBS: 500 mM, maltohexaose: 100 pmol).
