# Supplementary material for: High-throughput glycolytic inhibitor discovery targeting glioblastoma by graphite dots–assisted LDI mass spectrometry
Source: Sci Adv. 2022 Feb 16;8(7):eabl4923. doi: 10.1126/sciadv.abl4923 (PMC10921956; doi:10.1126/sciadv.abl4923)
Supplement: Supplementary file 2 — Data files S1 and S2 [file sciadv.abl4923_data_files_s1_and_s2.zip › sciadv.abl4923_data_file_s2.pdf]

Supplementary Data S2

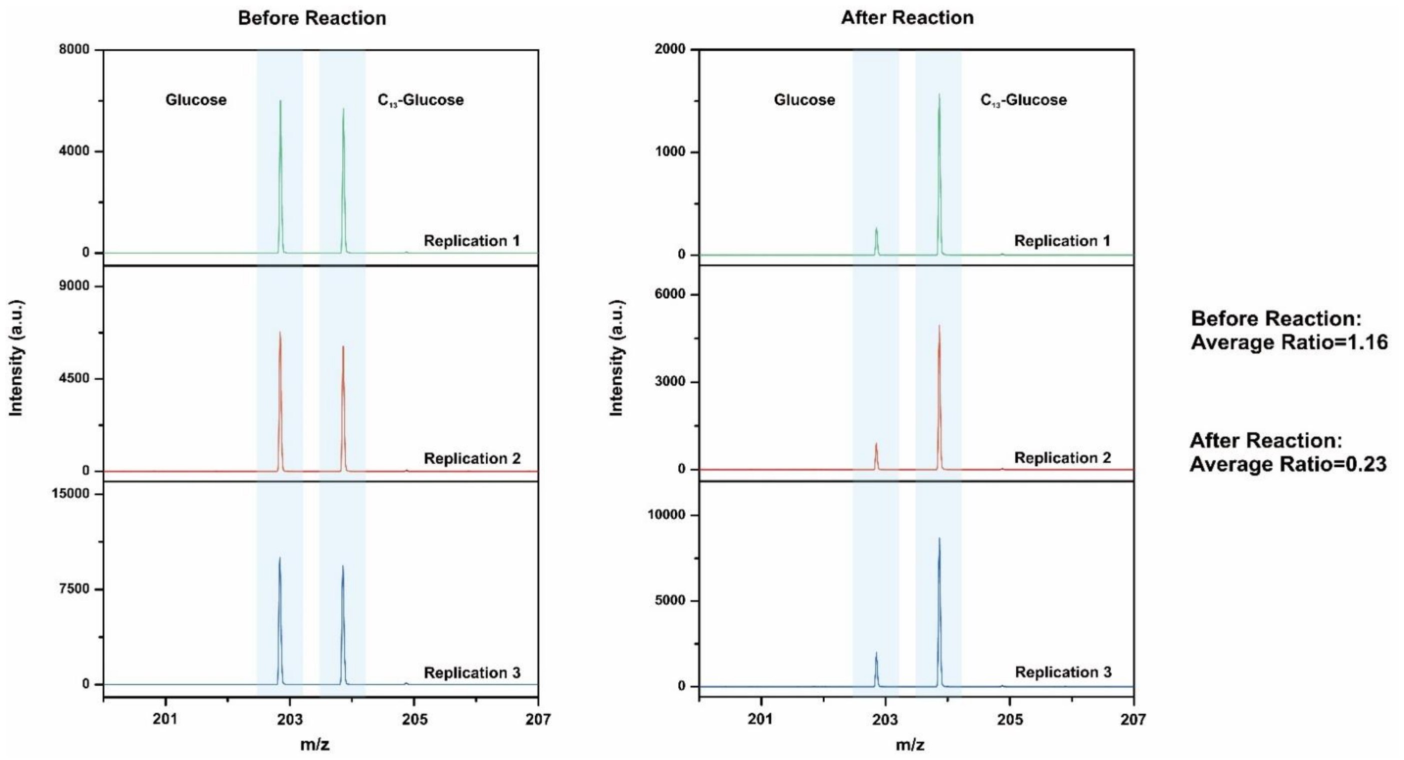

Raw data of HK2 activity using GLMSD platform. We obtained the reaction rate by comparing the glucose/^{13}C-glucose ratio before and after the reaction. Representative spectra (m/z 199-207) show high reproducibility for the replicates.

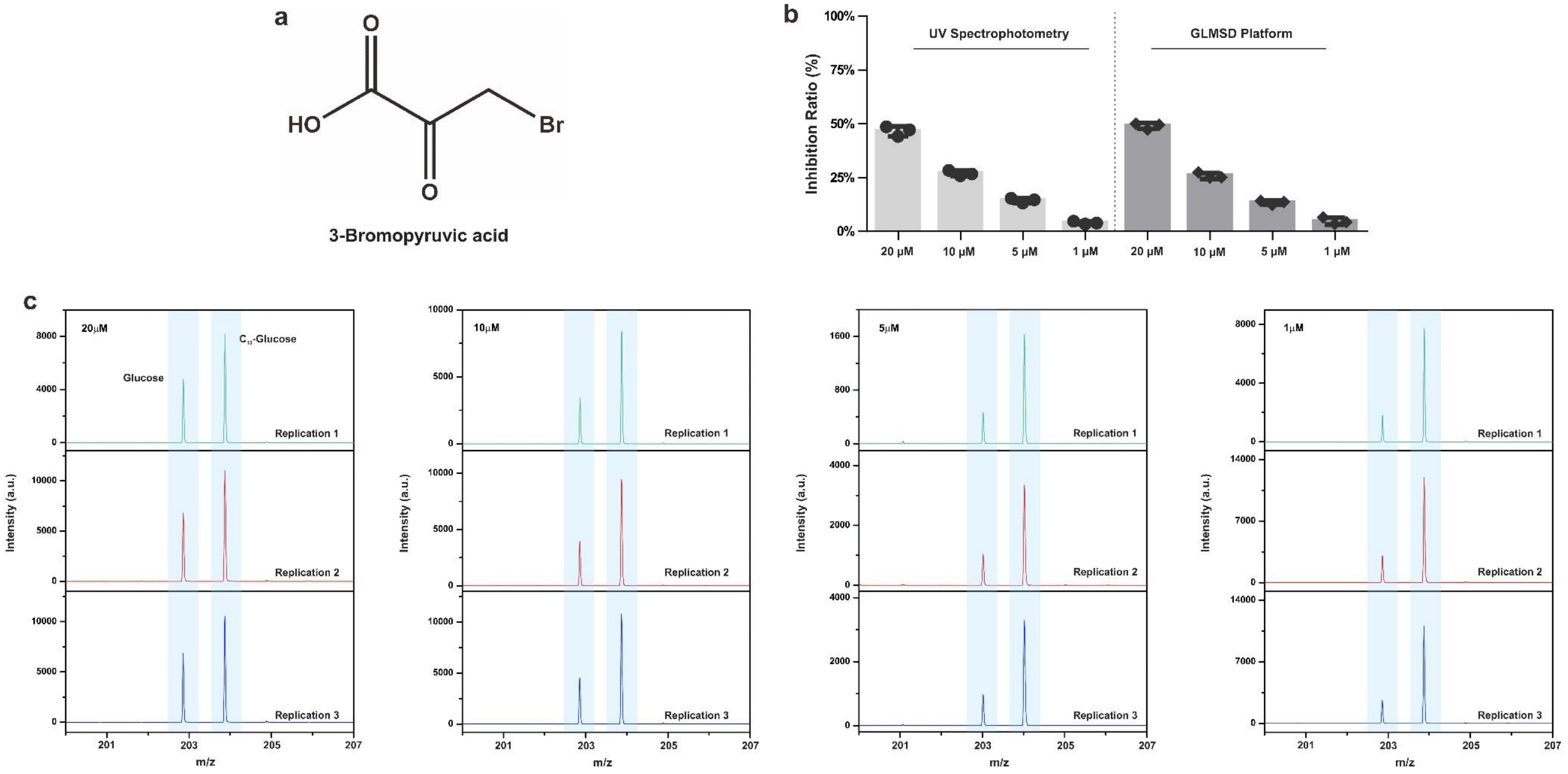

(a) Chemical structures of 3-BP (b) Inhibition profiles of 3-BP against the HK2 enzyme obtained from colorimetric kit and GLMSD. (c) Raw data of HK2 activity in four concentrations of 3-BP detected by GLMSD platform.

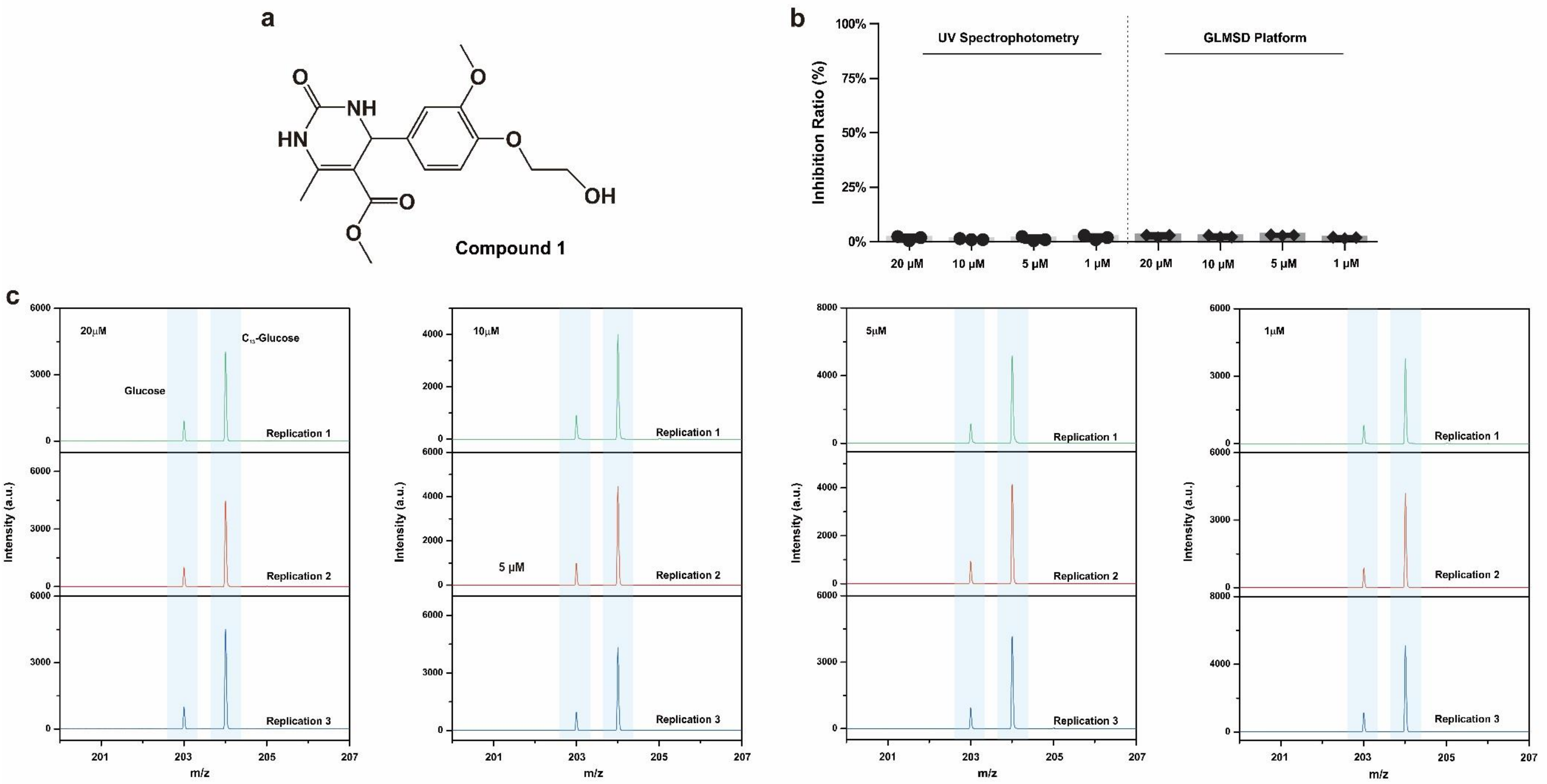

(a) Chemical structure of compound 1. (b) Inhibition profiles of compound 1 against the HK2 enzyme obtained from colorimetric kit and GLMSD. (c) Raw data of HK2 activity in four concentrations of compound 1 detected by GLMSD platform.

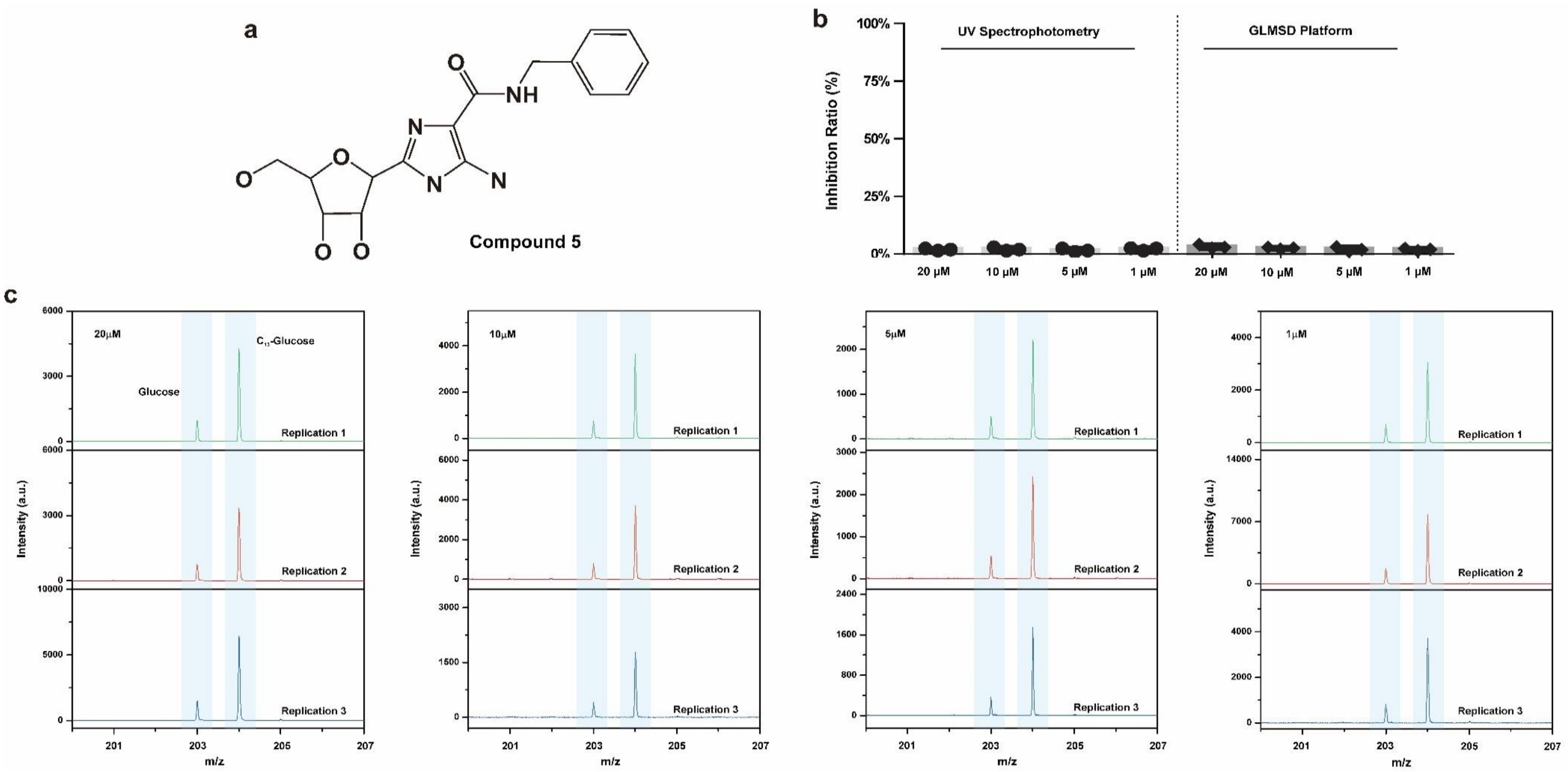

(a) Chemical structures of compound 5. (b) Inhibition profiles of compound 5 against the HK2 enzyme obtained from colorimetric kit and GLMSD. (c) Raw data of HK2 activity in four concentrations of compound 5 detected by GLMSD platform.

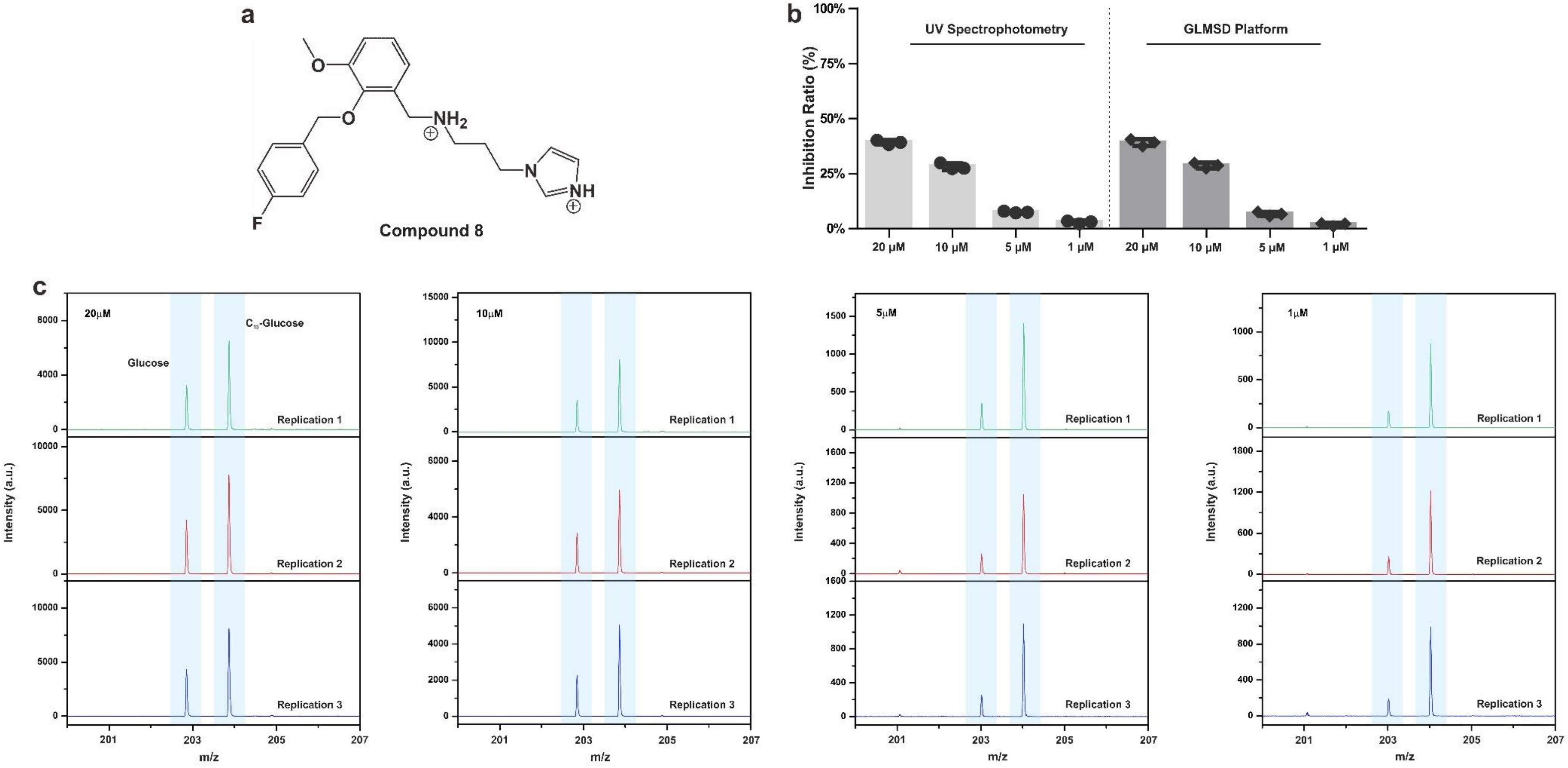

(a) Chemical structure of compound 8. (b) Inhibition profiles of compound 8 against the HK2 enzyme obtained from colorimetric kit and GLMSD. (c) Raw data of HK2 activity in four concentrations of compound 8 detected by GLMSD platform.

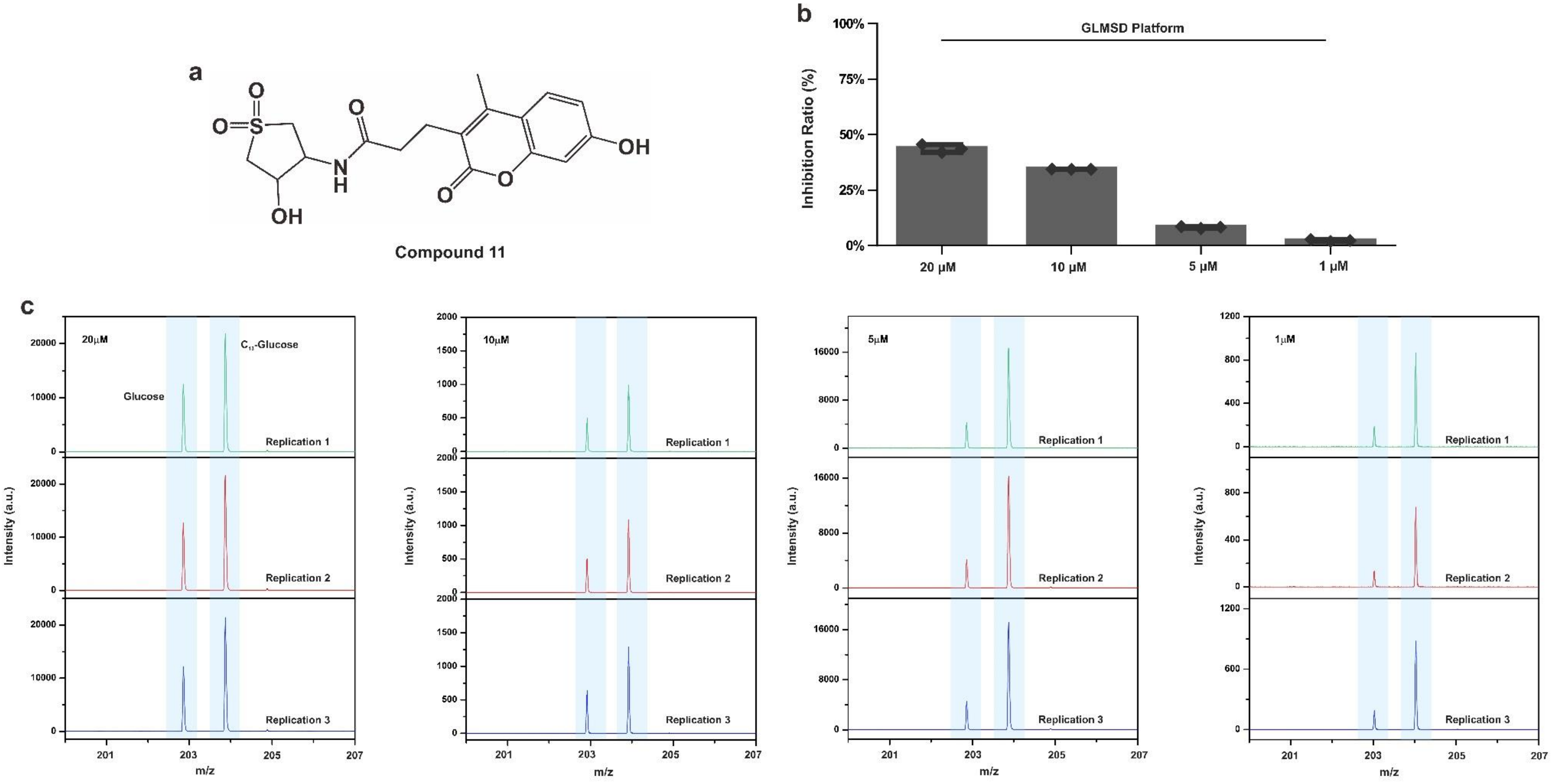

(a) Chemical structure of compound 11. (b) Inhibition profiles of compound 11 against the HK2 enzyme obtained from colorimetric kit and GLMSD. (c) Raw data of HK2 activity in four concentrations of compound 11 detected by GLMSD platform.

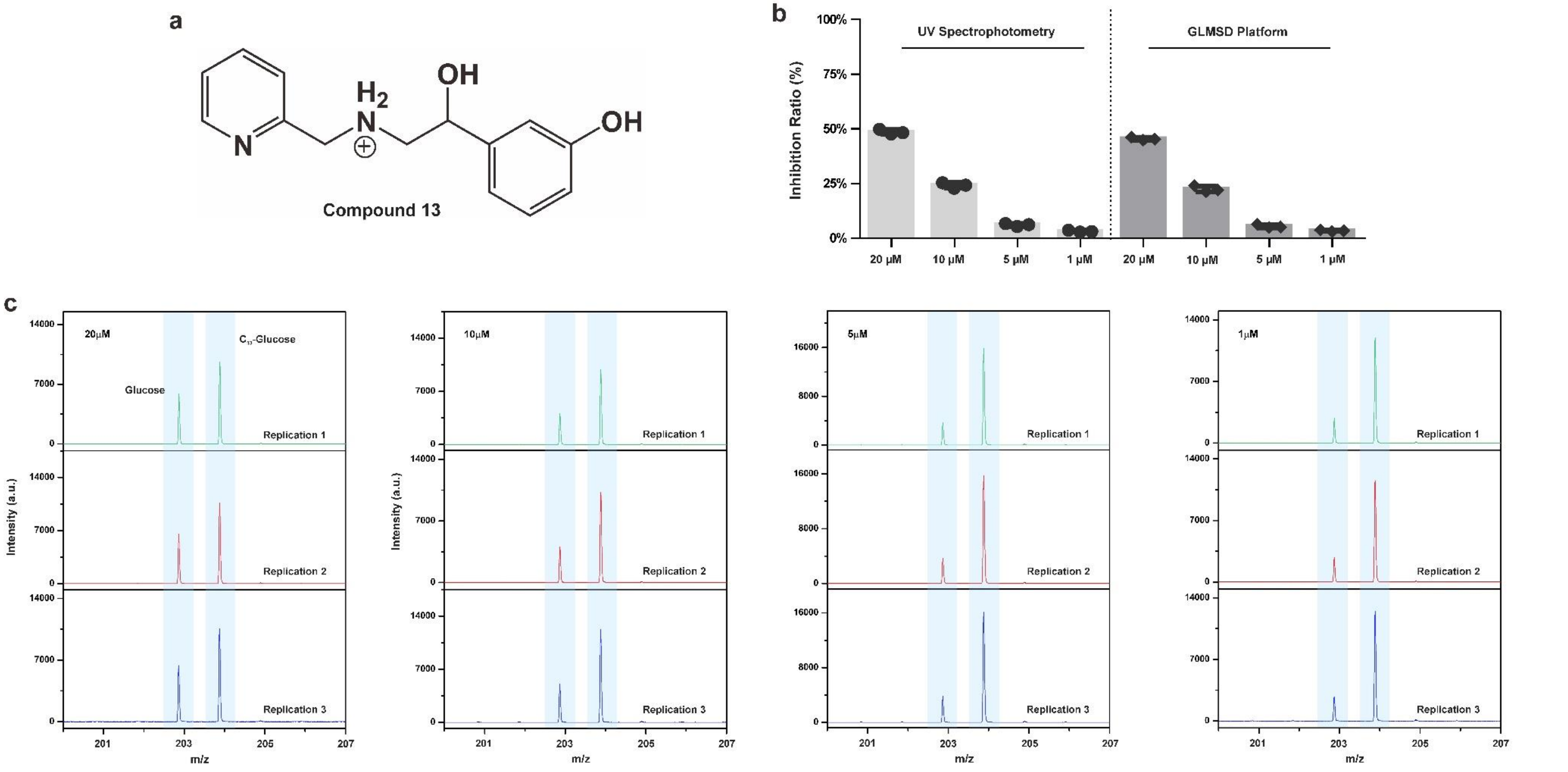

(a) Chemical structures of compound 13. (b) Inhibition profiles of compound 13 against the HK2 enzyme obtained from colorimetric kit and GLMSD (c) Raw data of HK2 activity in four concentrations of compound 13 detected by GLMSD platform.

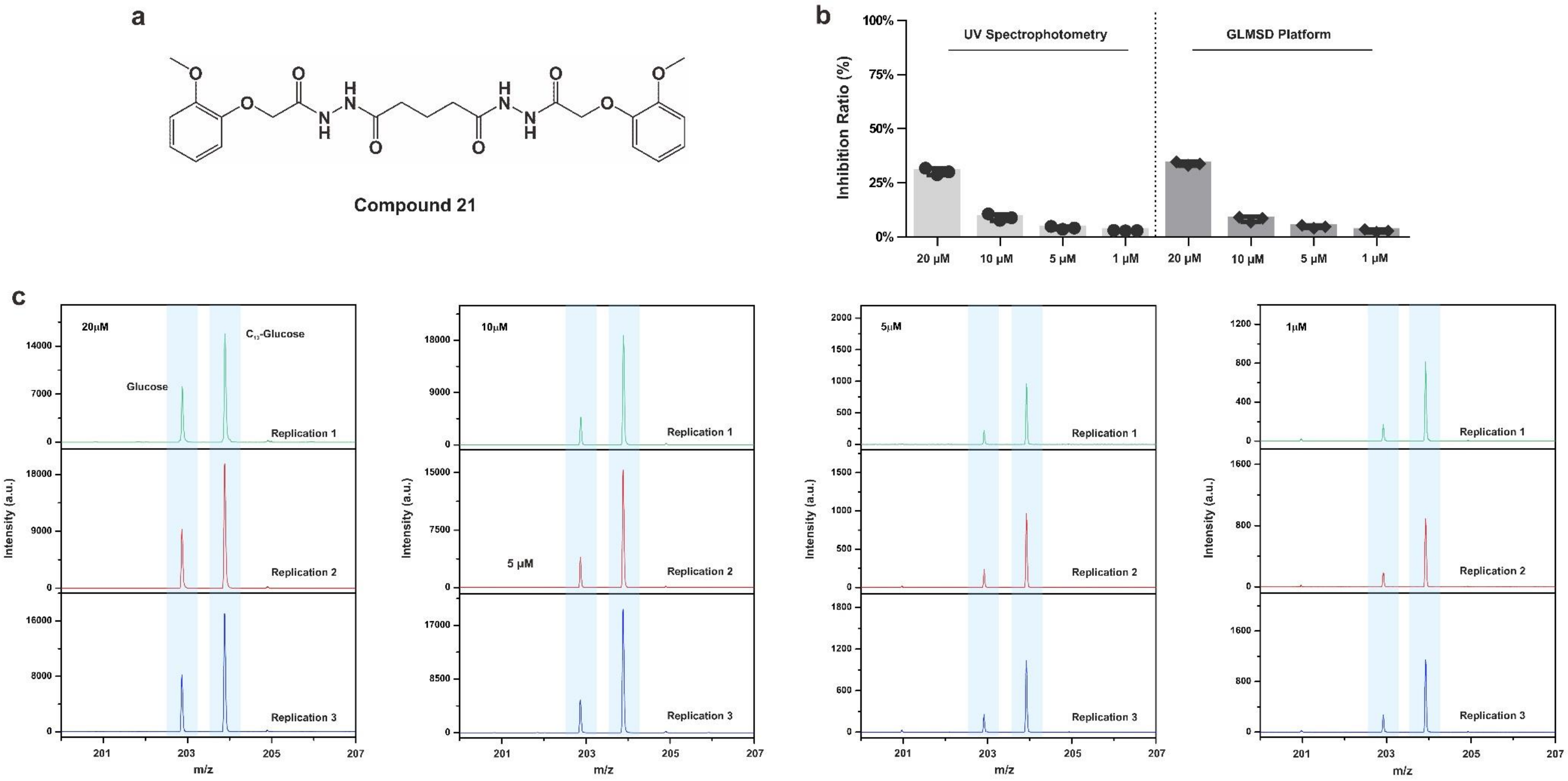

(a) Chemical structures of compound 21. (b) Inhibition profiles of compound 21 against the HK2 enzyme obtained from colorimetric kit and GLMSD (c) Raw data of HK2 activity in four concentrations of compound 21 detected by GLMSD platform.

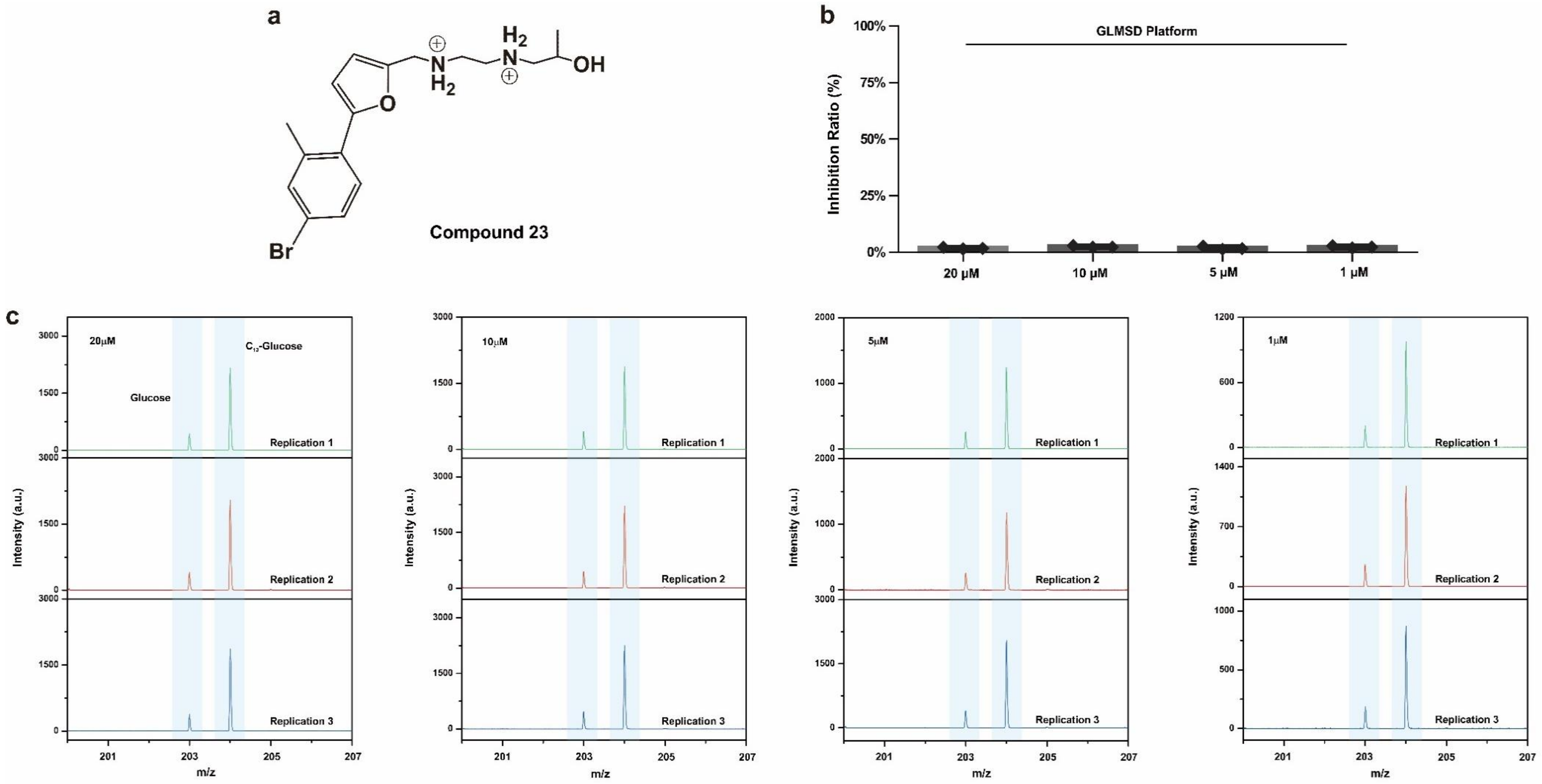

(a) Chemical structures of compound 23. (b) Inhibition profiles of compound 23 against the HK2 enzyme obtained from GLMSD (c) Raw data of HK2 activity in four concentrations of compound 23 detected by GLMSD platform.

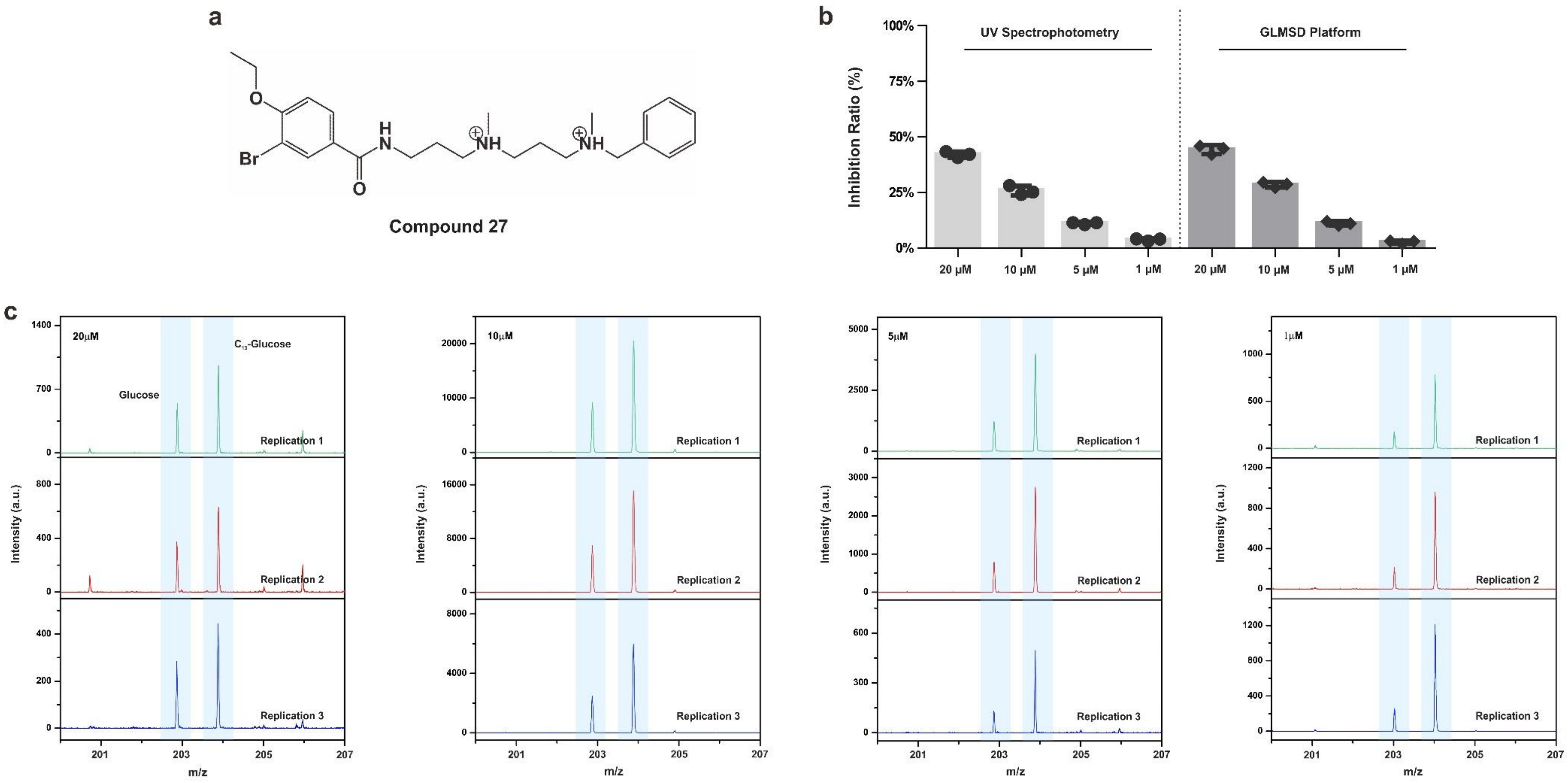

(a) Chemical structures of compound 27. (b) Inhibition profiles of compound 27 against the HK2 enzyme obtained from colorimetric kit and GLMSD (c) Raw data of HK2 activity in four concentrations of compound 27 detected by GLMSD platform.

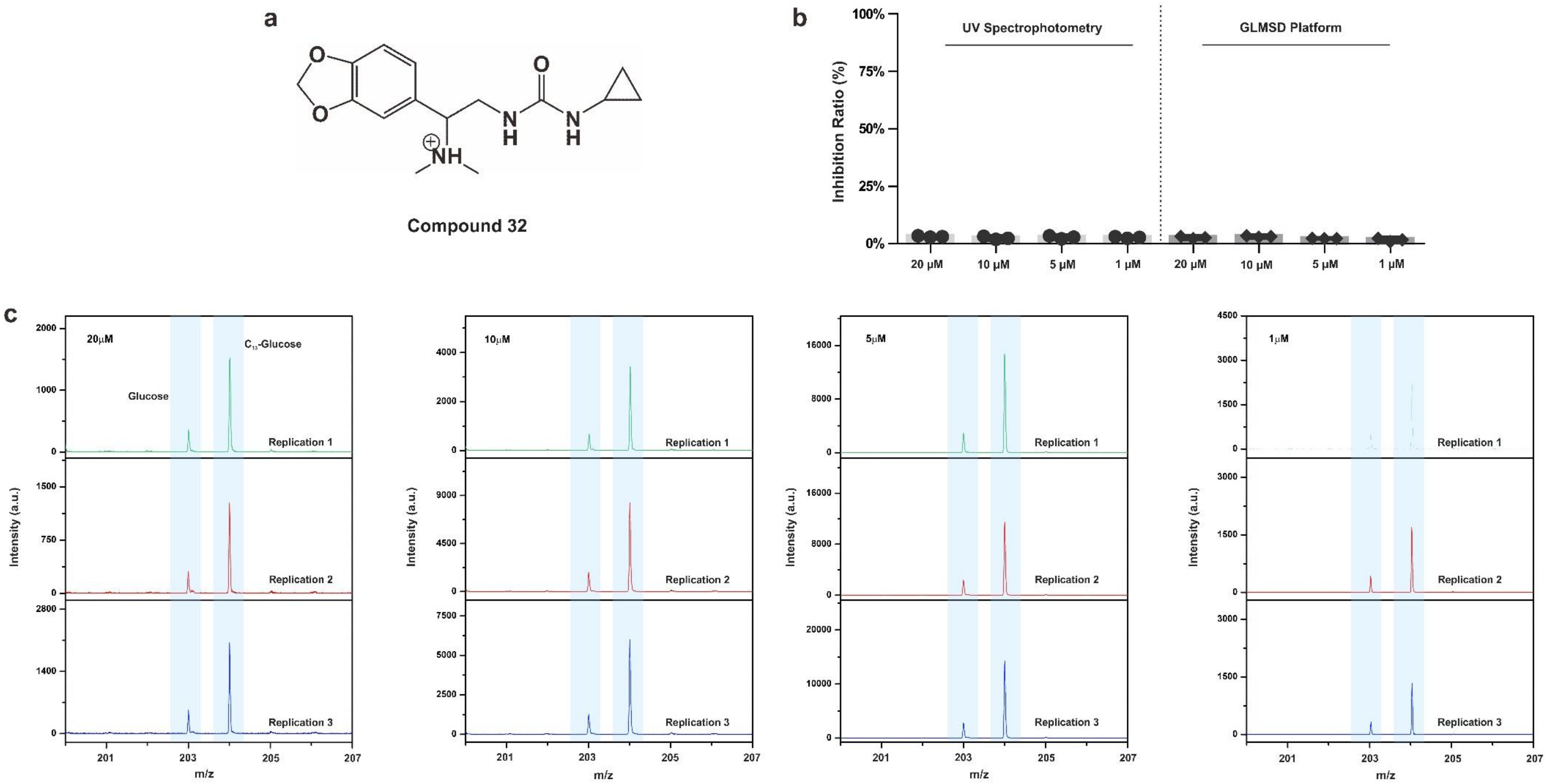

(a) Chemical structures of compound 32. (b) Inhibition profiles of compound 32 against the HK2 enzyme obtained from colorimetric kit and GLMSD (c) Raw data of HK2 activity in four concentrations of compound 32 detected by GLMSD platform.

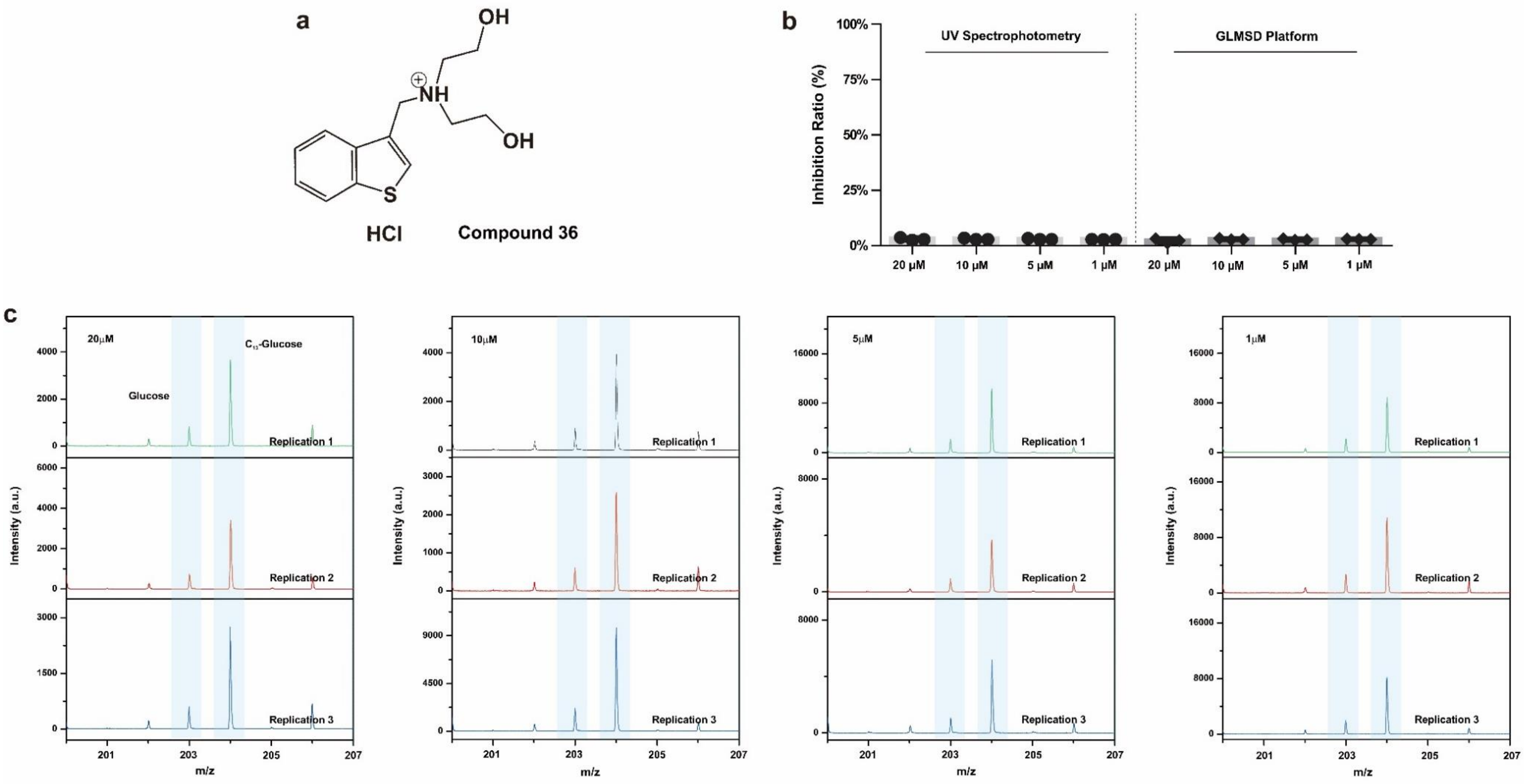

(a) Chemical structures of compound 36. (b) Inhibition profiles of compound 36 against the HK2 enzyme obtained from colorimetric kit and GLMSD (c) Raw data of HK2 activity in four concentrations of compound 36 detected by GLMSD platform.

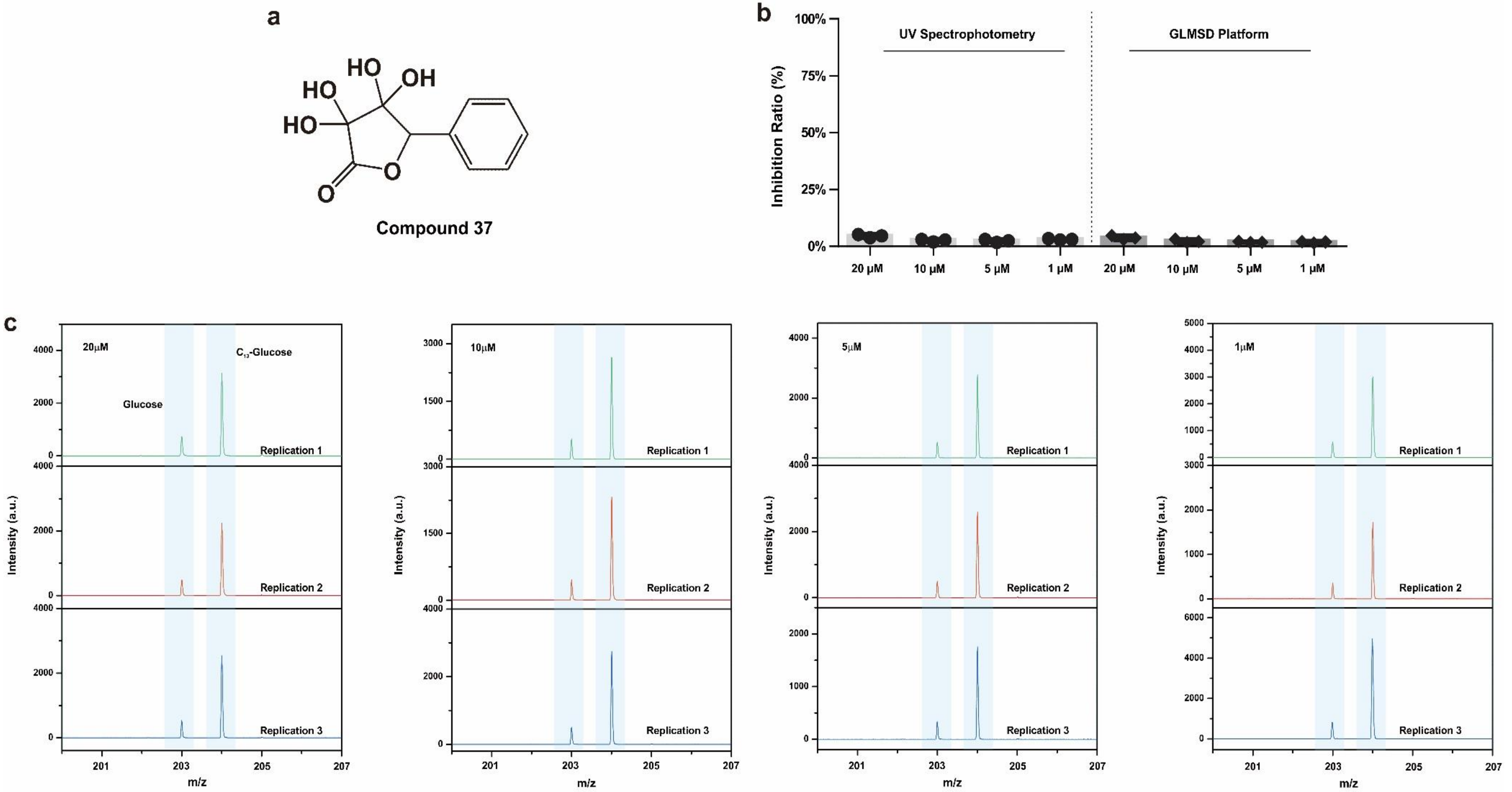

(a) Chemical structures of compound 37. (b) Inhibition profiles of compound 37 against the HK2 enzyme obtained from colorimetric kit and GLMSD (c) Raw data of HK2 activity in four concentrations of compound 37 detected by GLMSD platform.

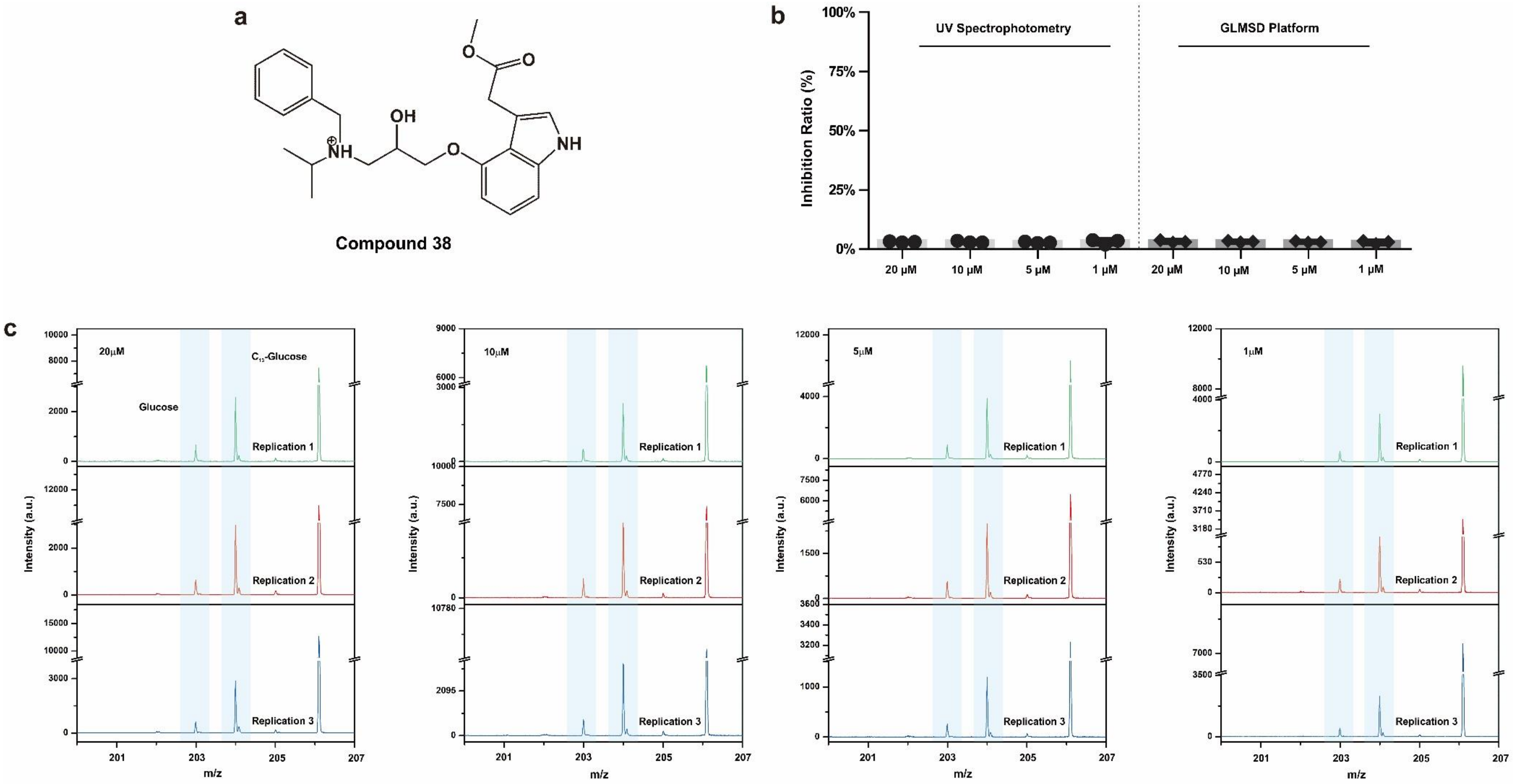

(a) Chemical structures of compound 38. (b) Inhibition profiles of compound 38 against the HK2 enzyme obtained from colorimetric kit and GLMSD (c) Raw data of HK2 activity in four concentrations of compound 38 detected by GLMSD platform.

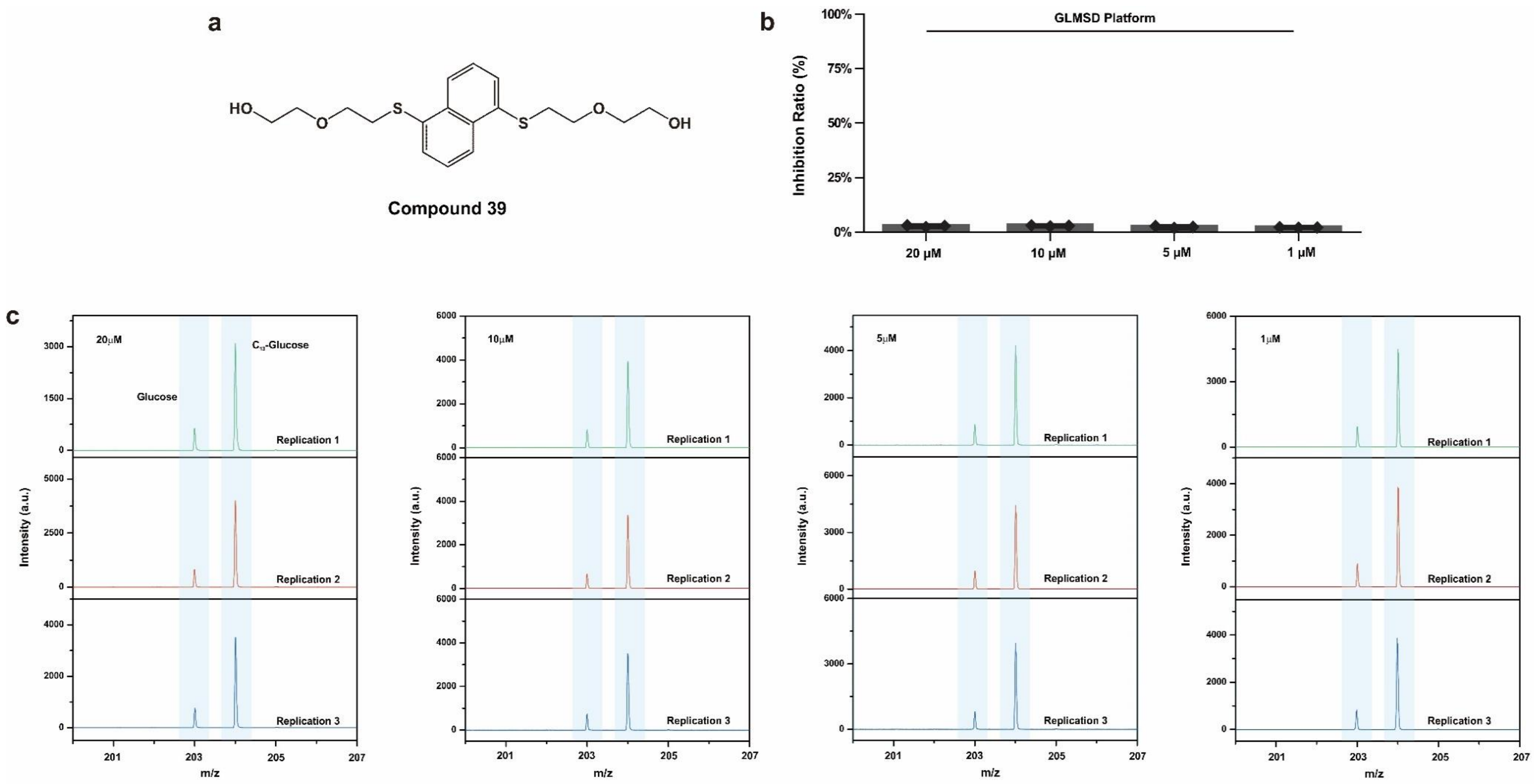

(a) Chemical structures of compound 39. (b) Inhibition profiles of compound 39 against the HK2 enzyme obtained from GLMSD (c) Raw data of HK2 activity in four concentrations of compound 38 detected by GLMSD platform

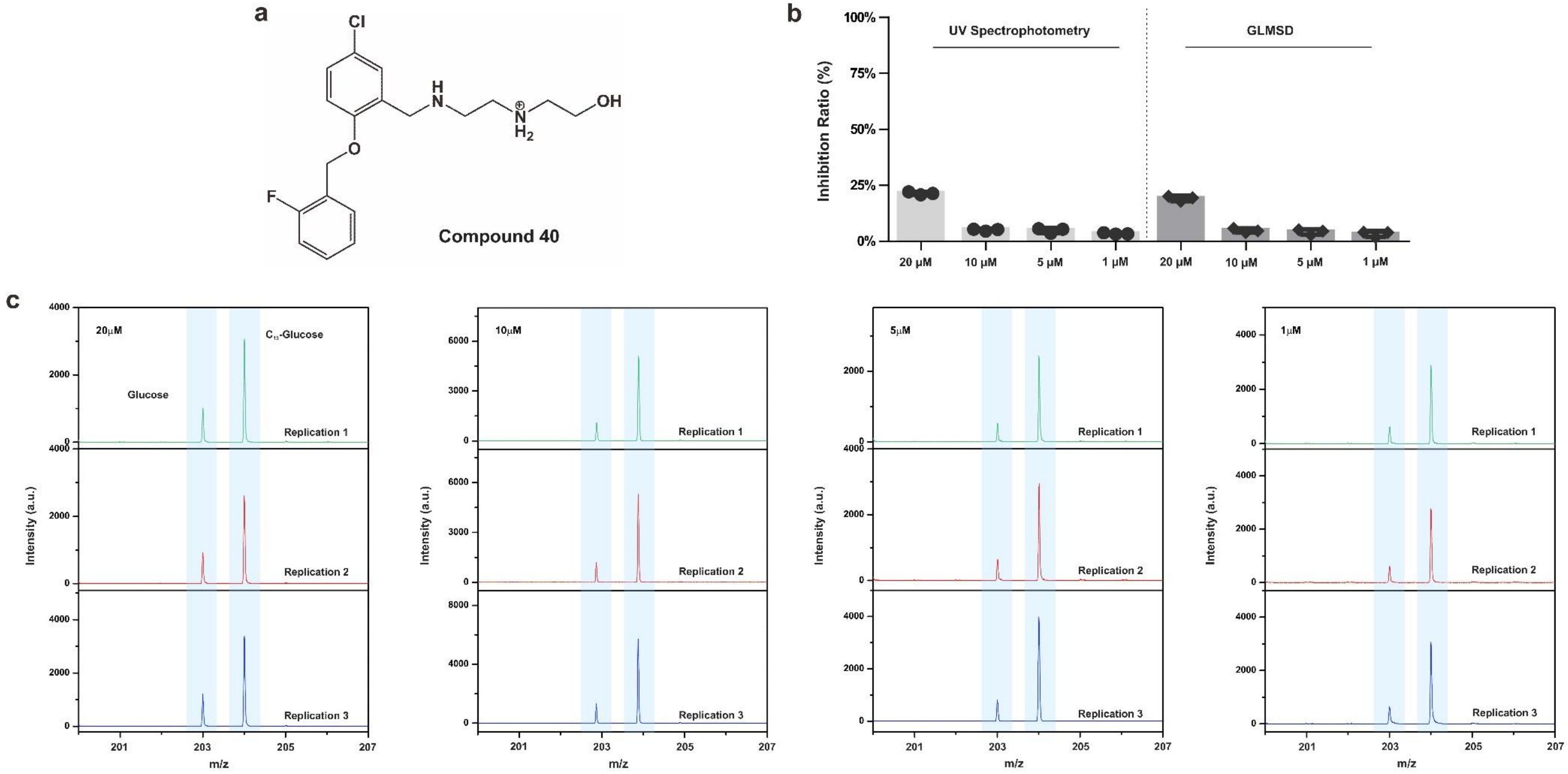

(a) Chemical structures of compound 40. (b) Inhibition profiles of compound 40 against the HK2 enzyme obtained from colorimetric kit and GLMSD platform. (c) Raw data of HK2 activity in four concentrations of compound 40 detected by GLMSD platform.

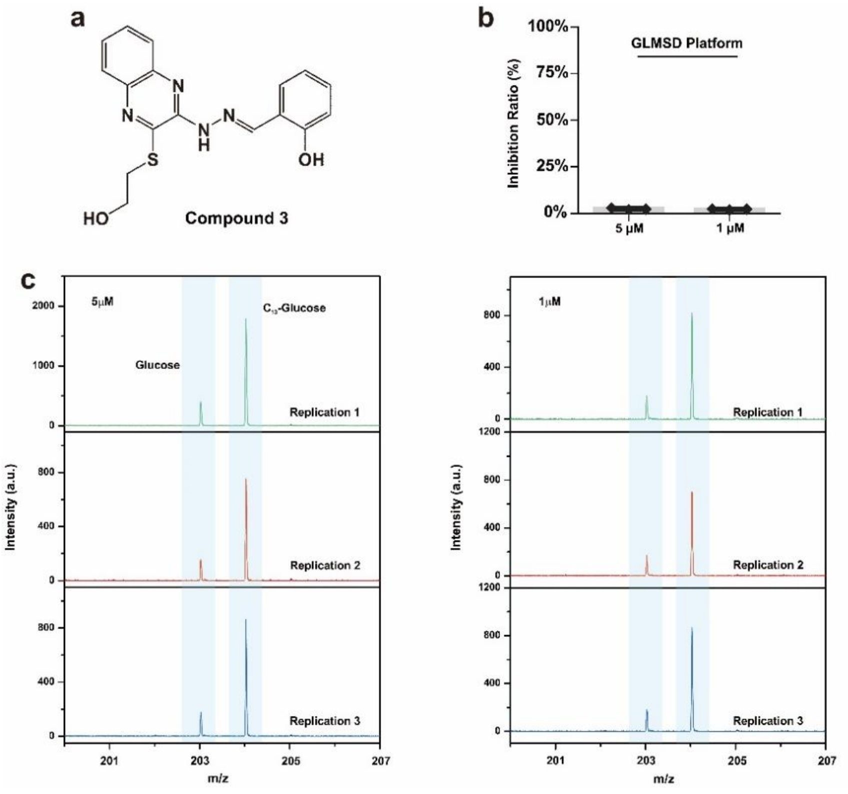

(a) Chemical structures of compound 3. (b) Inhibition profiles of compound 3 against the HK2 enzyme obtained from GLMSD platform. (c) Raw data of HK2 activity in four concentrations of compound 3 detected by GLMSD platform.

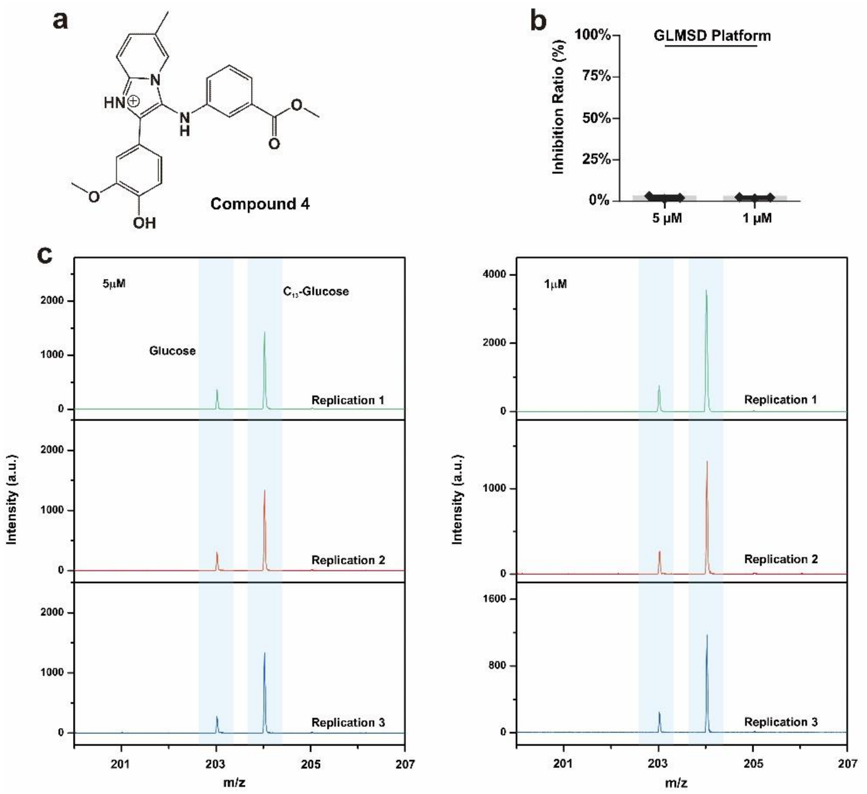

(a) Chemical structures of compound 4. (b) Inhibition profiles of compound 4 against the HK2 enzyme obtained from GLMSD platform. (c) Raw data of HK2 activity in four concentrations of compound 4 detected by GLMSD platform.

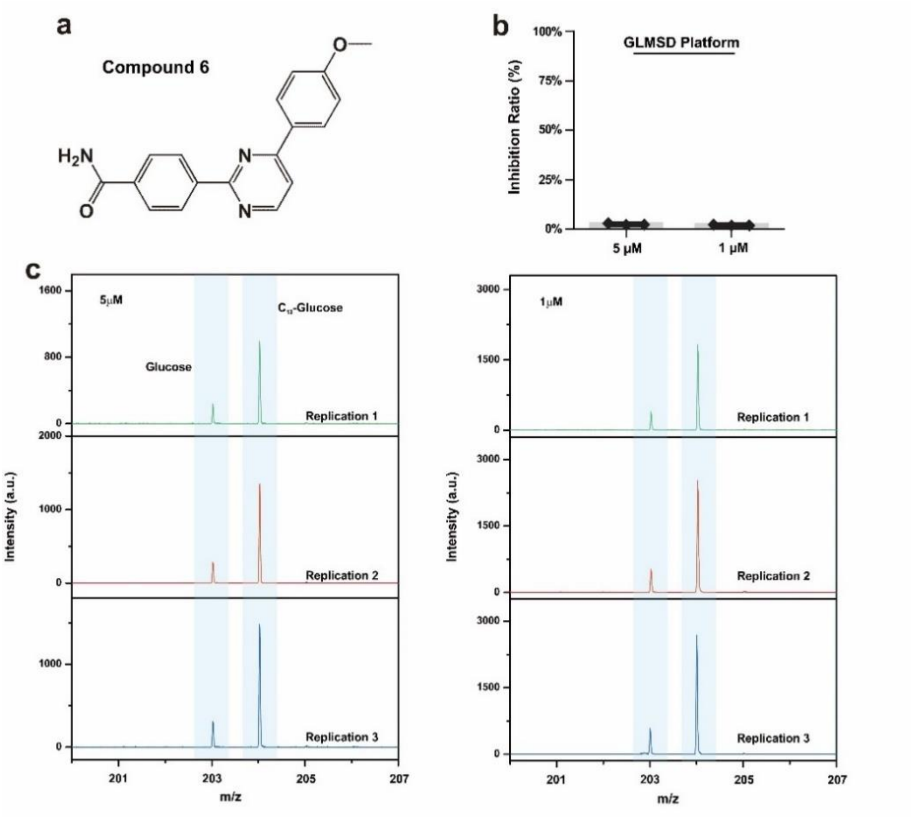

(a) Chemical structures of compound 6. (b) Inhibition profiles of compound 6 against the HK2 enzyme obtained from GLMSD platform. (c) Raw data of HK2 activity in four concentrations of compound 6 detected by GLMSD platform.

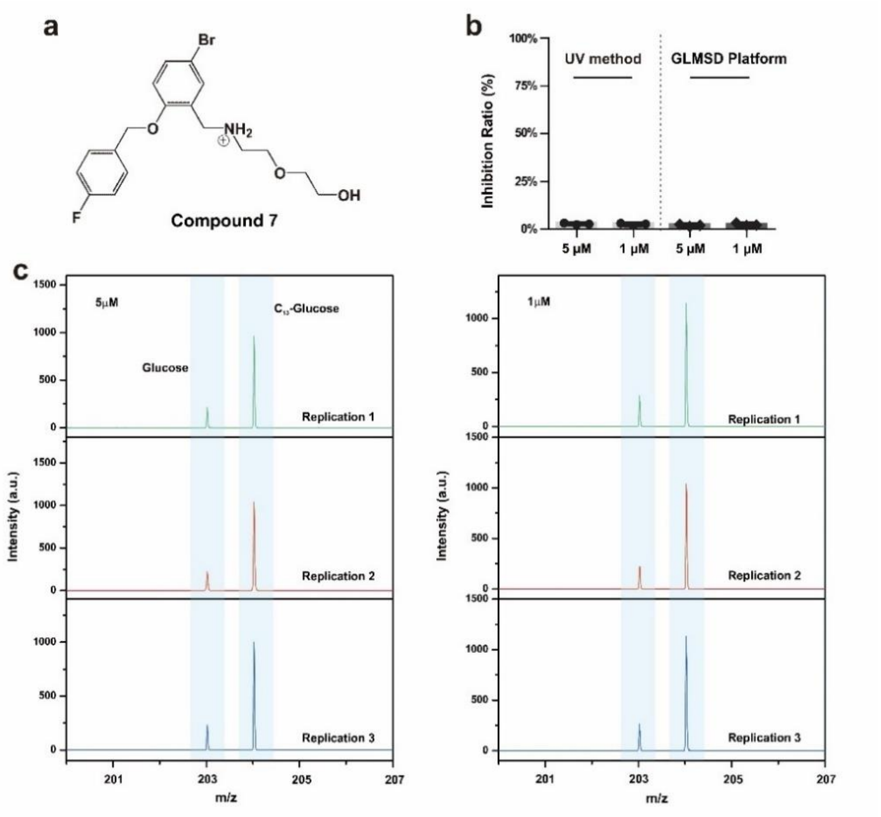

(a) Chemical structures of compound 7. (b) Inhibition profiles of compound 7 against the HK2 enzyme obtained from colorimetric kit and GLMSD platform. (c) Raw data of HK2 activity in four concentrations of compound 7 detected by GLMSD platform.

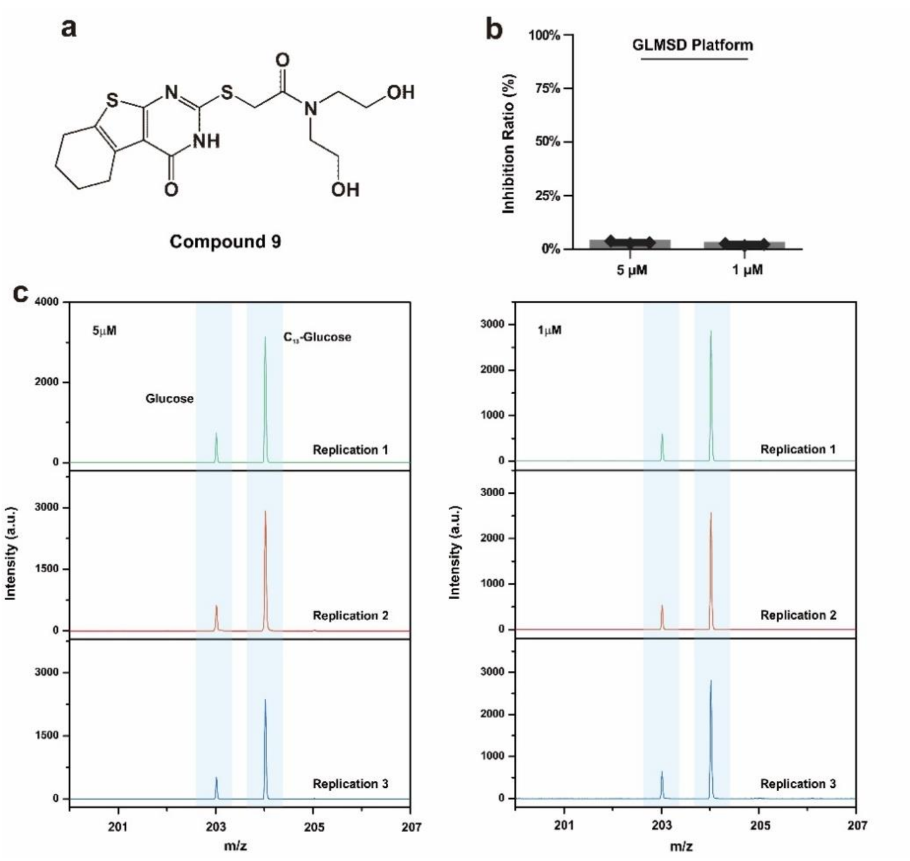

(a) Chemical structures of compound 9. (b) Inhibition profiles of compound 9 against the HK2 enzyme obtained from GLMSD platform. (c) Raw data of HK2 activity in four concentrations of compound 9 detected by GLMSD platform.

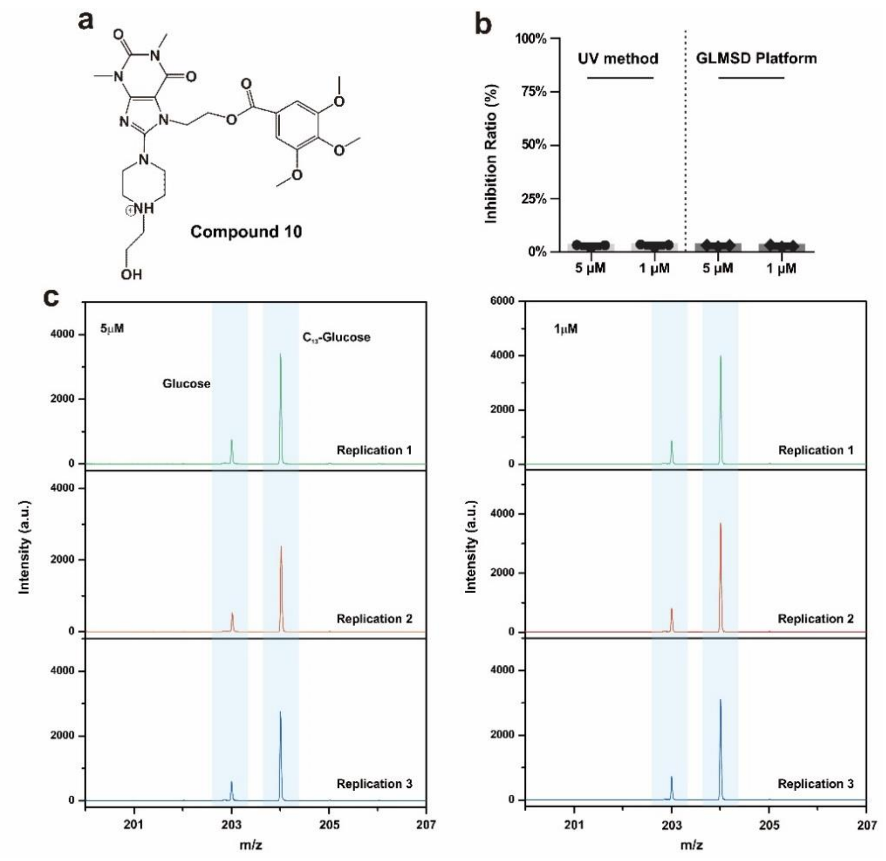

(a) Chemical structures of compound 10. (b) Inhibition profiles of compound 10 against the HK2 enzyme obtained from colorimetric kit and GLMSD platform. (c) Raw data of HK2 activity in four concentrations of compound 10 detected by GLMSD platform.

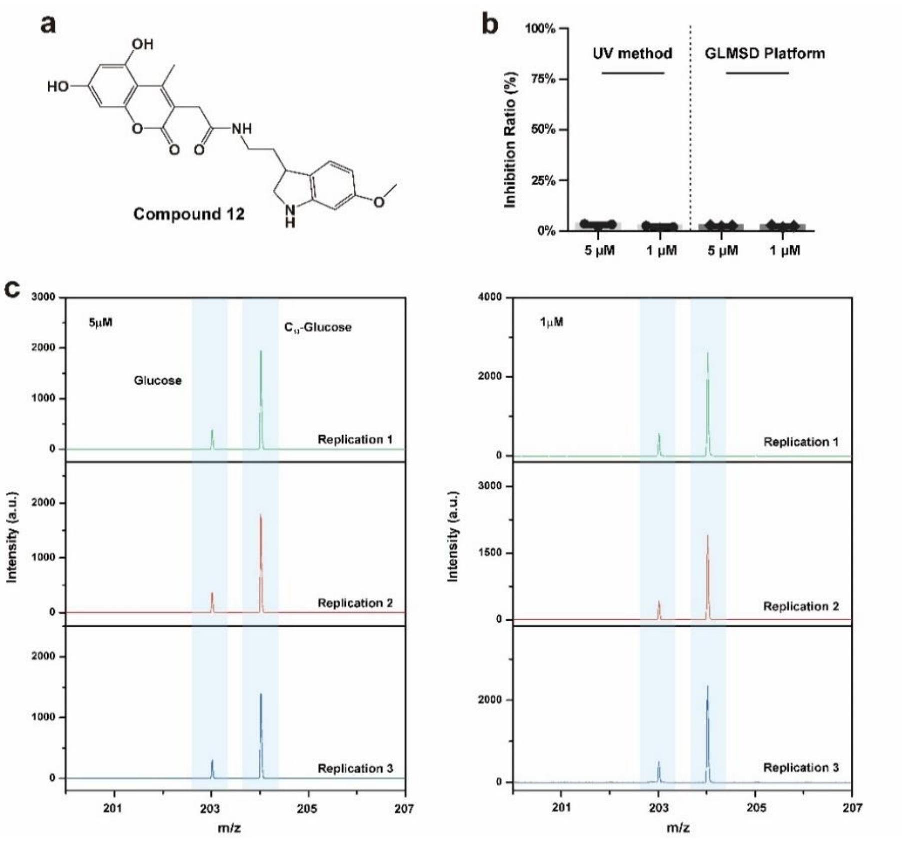

(a) Chemical structures of compound 12. (b) Inhibition profiles of compound 12 against the HK2 enzyme obtained from colorimetric kit and GLMSD platform. (c) Raw data of HK2 activity in four concentrations of compound 12 detected by GLMSD platform.

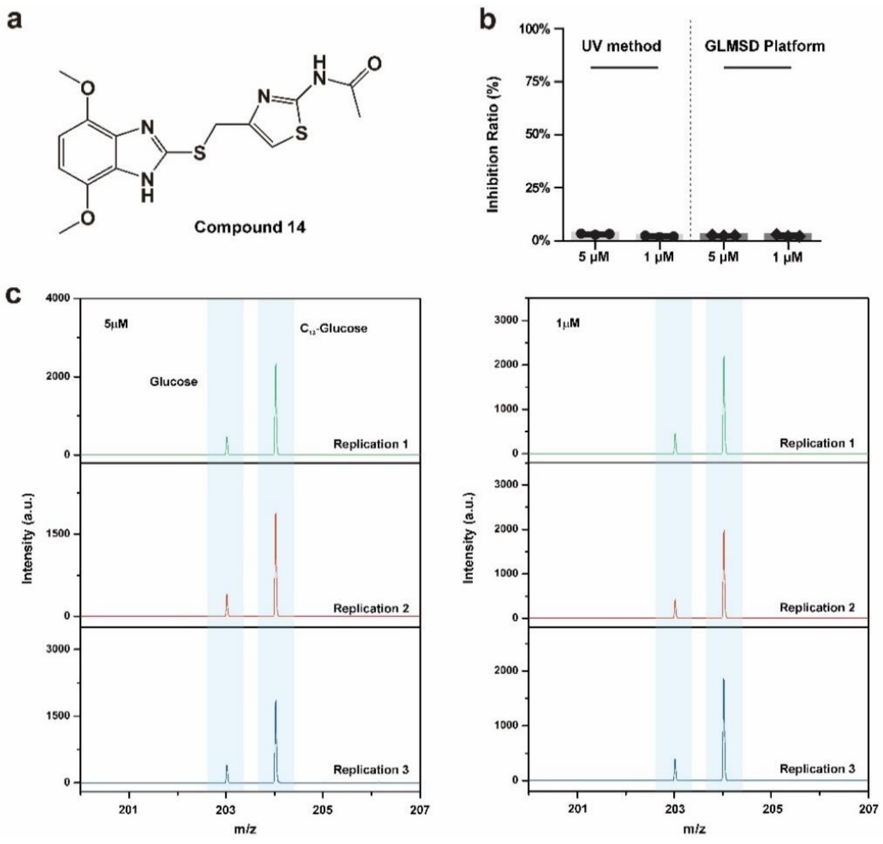

(a) Chemical structures of compound 14. (b) Inhibition profiles of compound 14 against the HK2 enzyme obtained from colorimetric kit and GLMSD platform. (c) Raw data of HK2 activity in four concentrations of compound 14 detected by GLMSD platform.

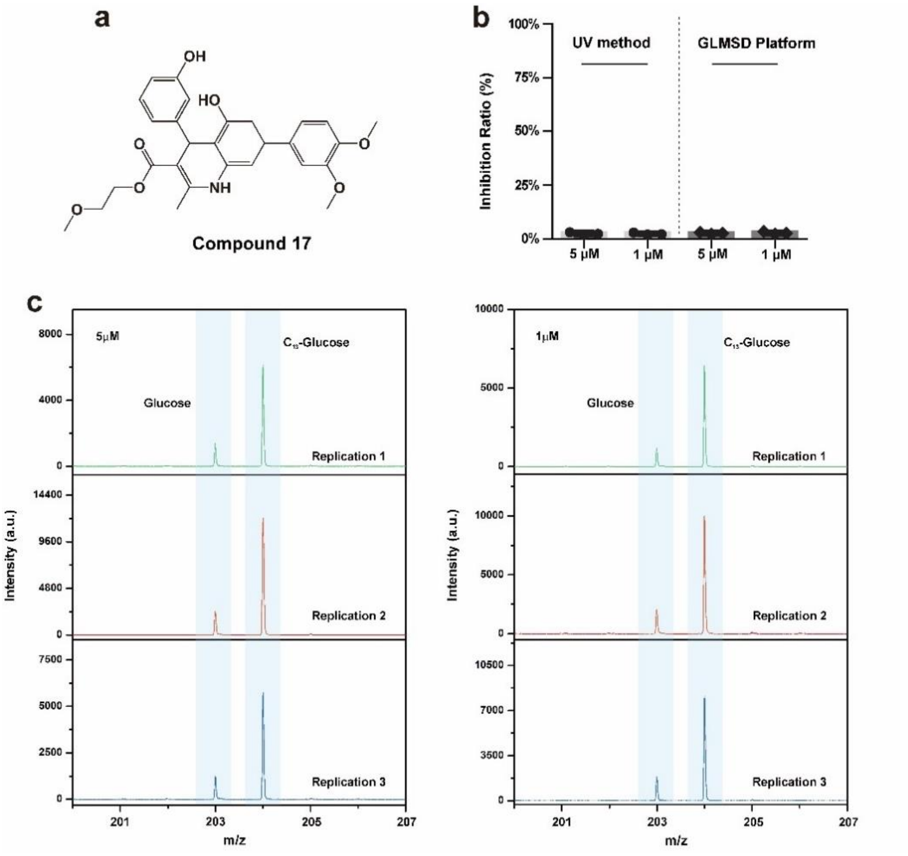

(a) Chemical structures of compound 17. (b) Inhibition profiles of compound 17 against the HK2 enzyme obtained from colorimetric kit and GLMSD platform. (c) Raw data of HK2 activity in four concentrations of compound 17 detected by GLMSD platform.

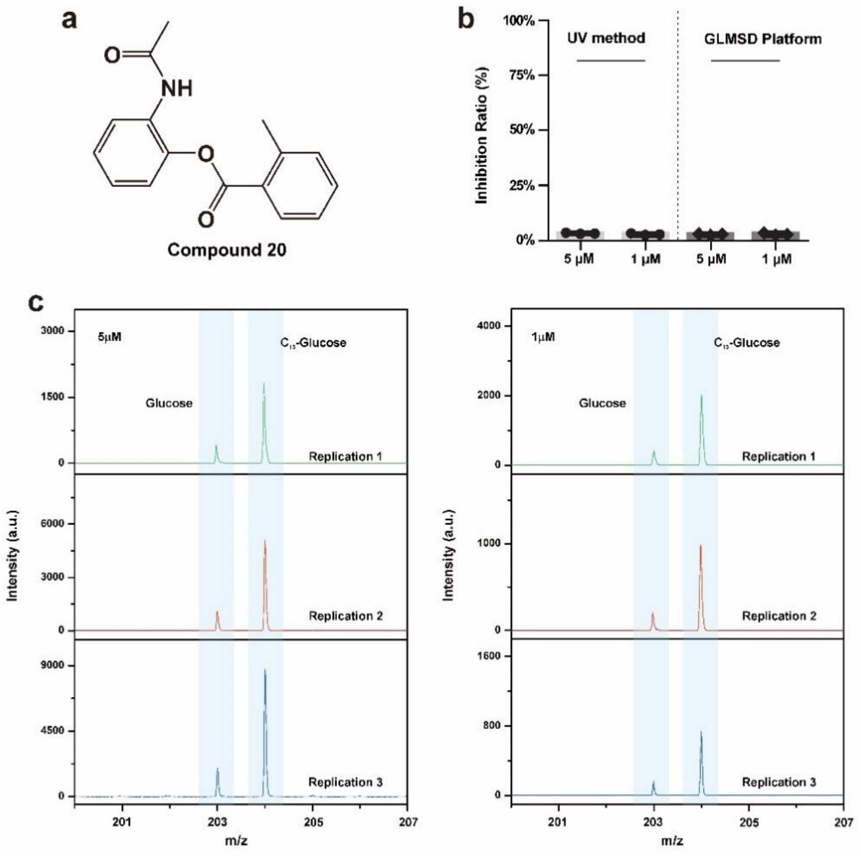

(a) Chemical structures of compound 20. (b) Inhibition profiles of compound 20 against the HK2 enzyme obtained from colorimetric kit and GLMSD platform. (c) Raw data of HK2 activity in four concentrations of compound 20 detected by GLMSD platform.

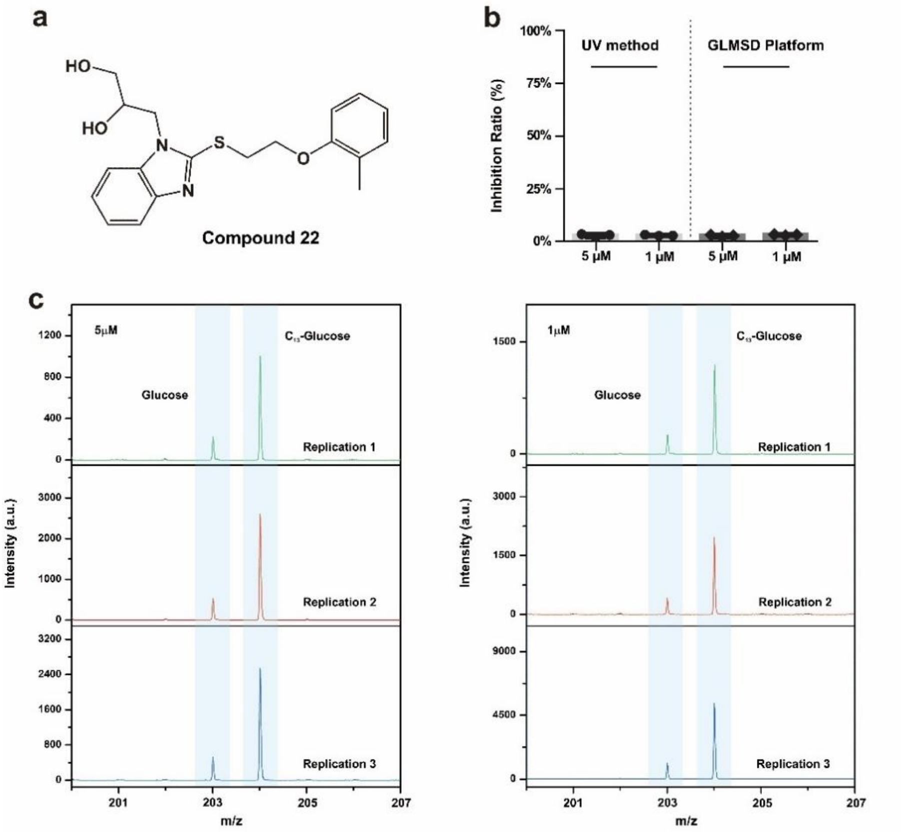

(a) Chemical structures of compound 22. (b) Inhibition profiles of compound 22 against the HK2 enzyme obtained from colorimetric kit and GLMSD platform. (c) Raw data of HK2 activity in four concentrations of compound 22 detected by GLMSD platform.

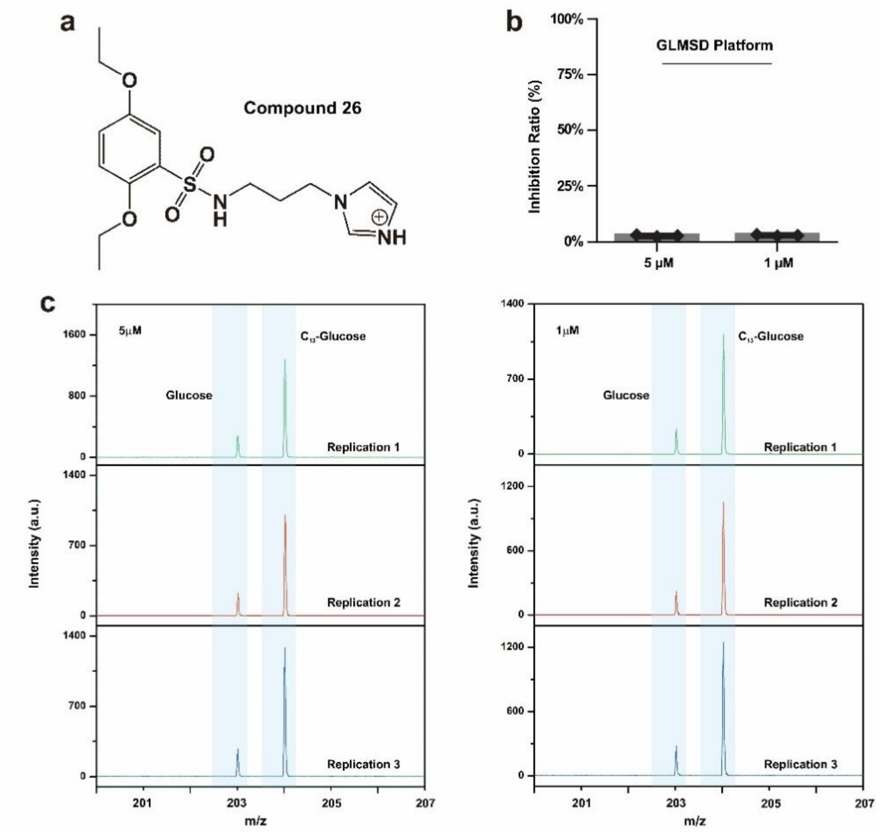

(a) Chemical structures of compound 26. (b) Inhibition profiles of compound 26 against the HK2 enzyme obtained from GLMSD platform. (c) Raw data of HK2 activity in four concentrations of compound 26 detected by GLMSD platform.

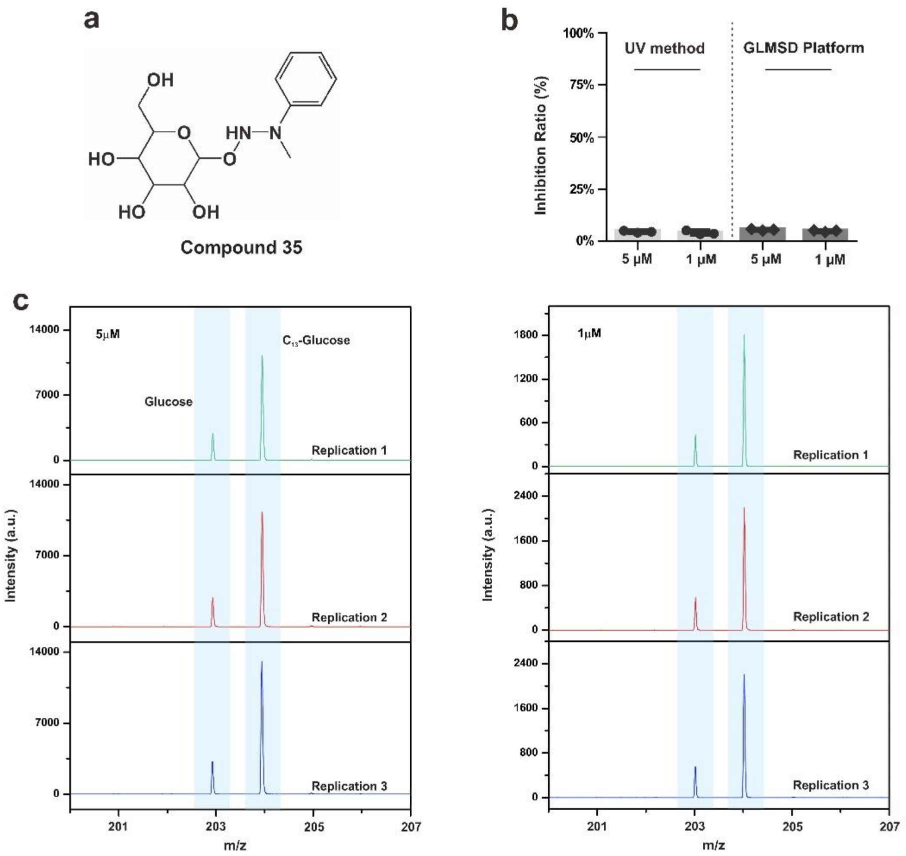

(a) Chemical structures of compound 35. (b) Inhibition profiles of compound 35 against the HK2 enzyme obtained from colorimetric kit and GLMSD platform. (c) Raw data of HK2 activity in four concentrations of compound 35 detected by GLMSD platform.

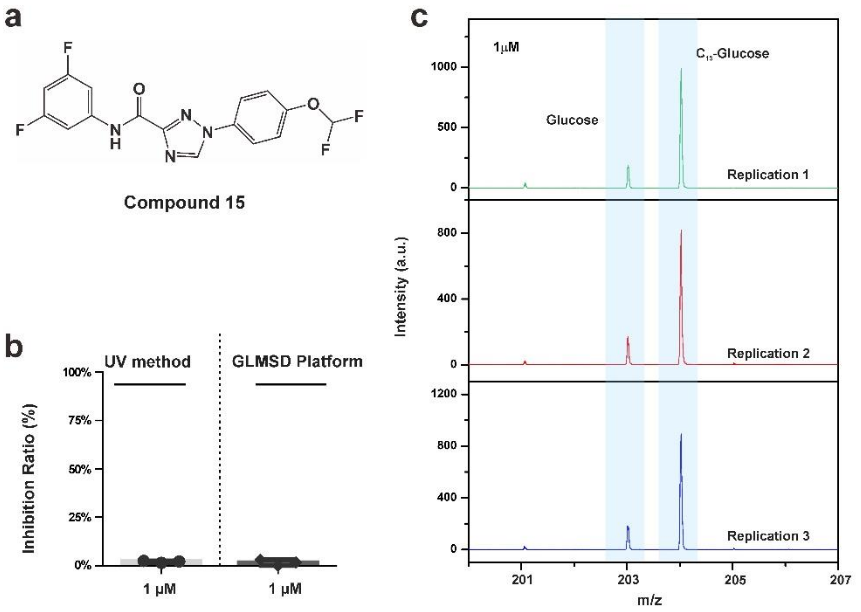

(a) Chemical structures of compound 15. (b) Inhibition profiles of compound 15 against the HK2 enzyme obtained from colorimetric kit and GLMSD platform. (c) Raw data of HK2 activity in four concentrations of compound 15 detected by GLMSD platform.

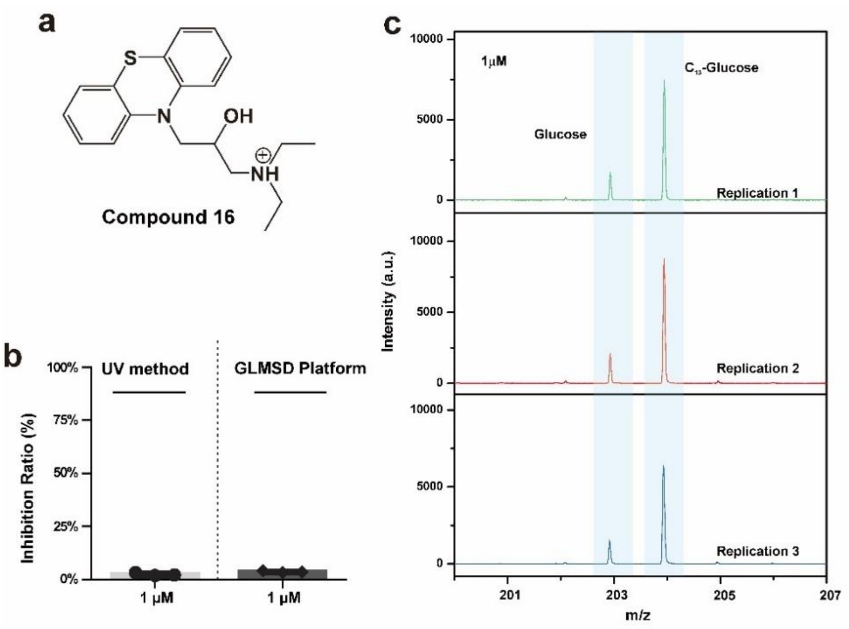

(a) Chemical structures of compound 16. (b) Inhibition profiles of compound 16 against the HK2 enzyme obtained from colorimetric kit and GLMSD platform. (c) Raw data of HK2 activity in four concentrations of compound 16 detected by GLMSD platform.

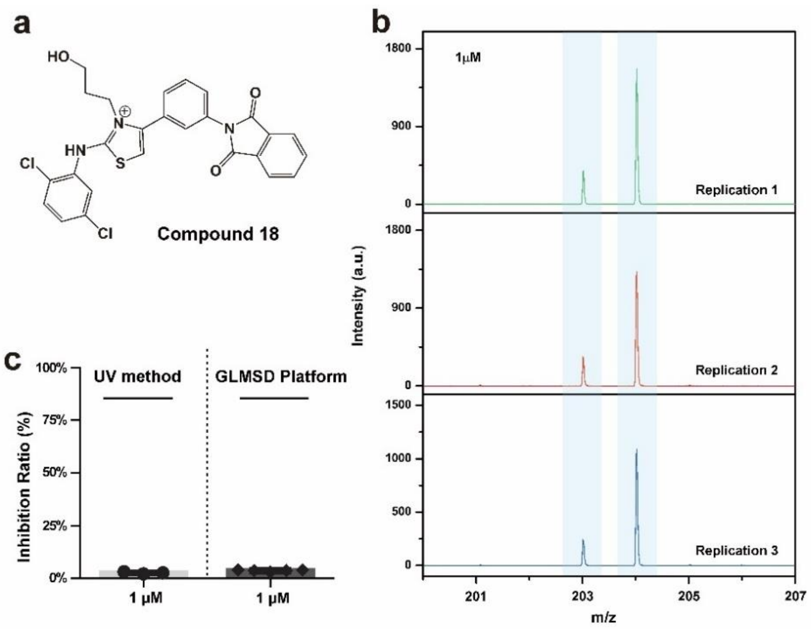

(a) Chemical structures of compound 18. (b) Inhibition profiles of compound 18 against the HK2 enzyme obtained from colorimetric kit and GLMSD platform. (c) Raw data of HK2 activity in four concentrations of compound 18 detected by GLMSD platform.

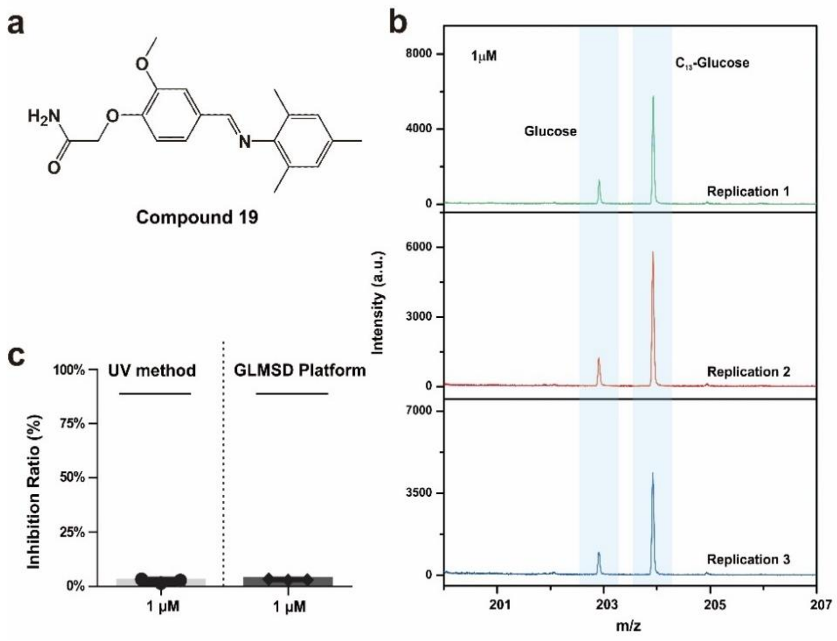

(a) Chemical structures of compound 19. (b) Inhibition profiles of compound 19 against the HK2 enzyme obtained from colorimetric kit and GLMSD platform. (c) Raw data of HK2 activity in four concentrations of compound 19 detected by GLMSD platform.

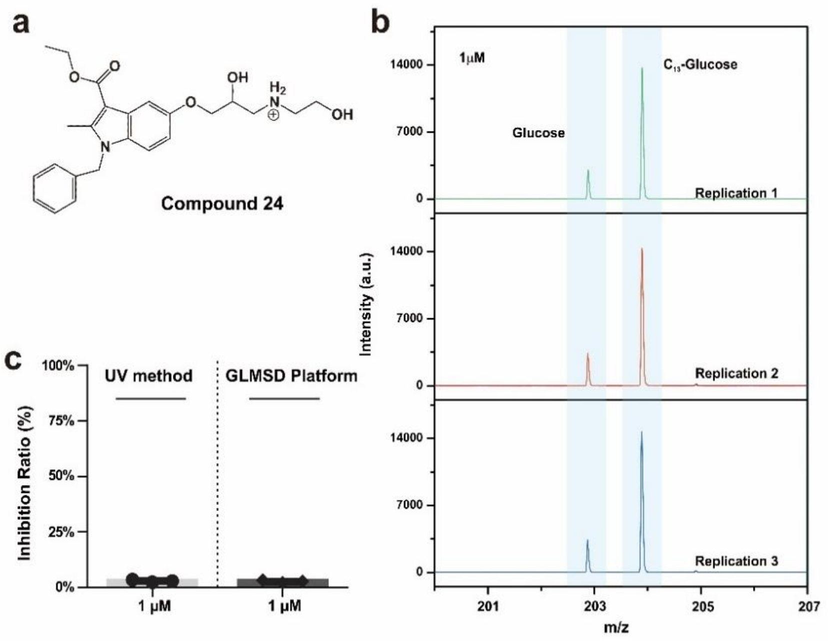

(a) Chemical structures of compound 24. (b) Inhibition profiles of compound 24 against the HK2 enzyme obtained from colorimetric kit and GLMSD platform. (c) Raw data of HK2 activity in four concentrations of compound 24 detected by GLMSD platform.

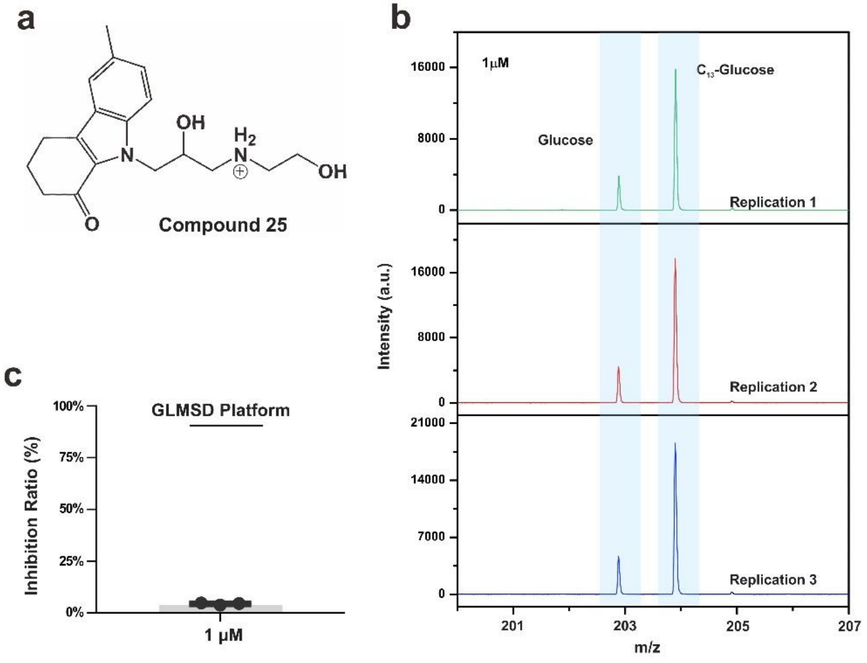

(a) Chemical structures of compound 25. (b) Inhibition profiles of compound 25 against the HK2 enzyme obtained from GLMSD platform. (c) Raw data of HK2 activity in four concentrations of compound 25 detected by GLMSD platform.

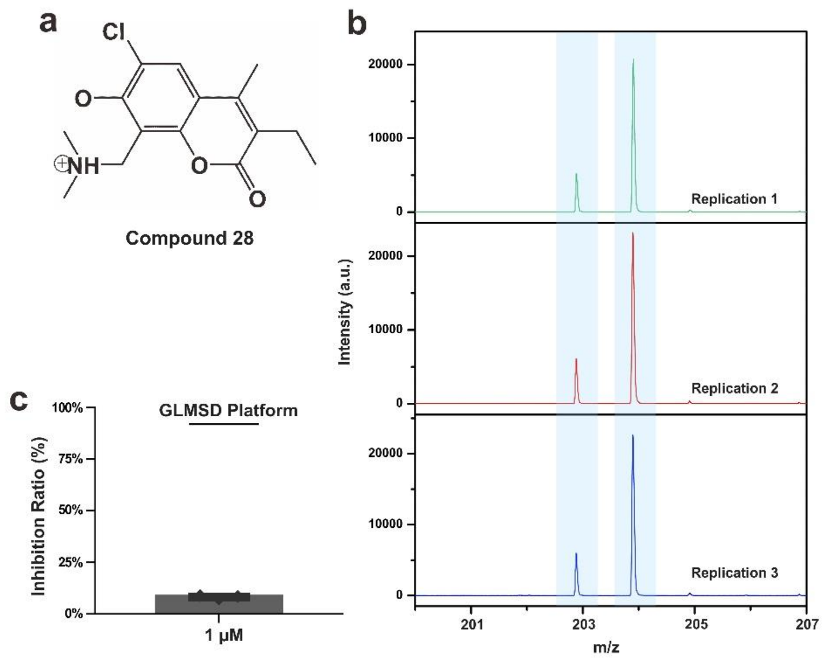

(a) Chemical structures of compound 28. (b) Inhibition profiles of compound 28 against the HK2 enzyme obtained from GLMSD platform. (c) Raw data of HK2 activity in four concentrations of compound 28 detected by GLMSD platform.

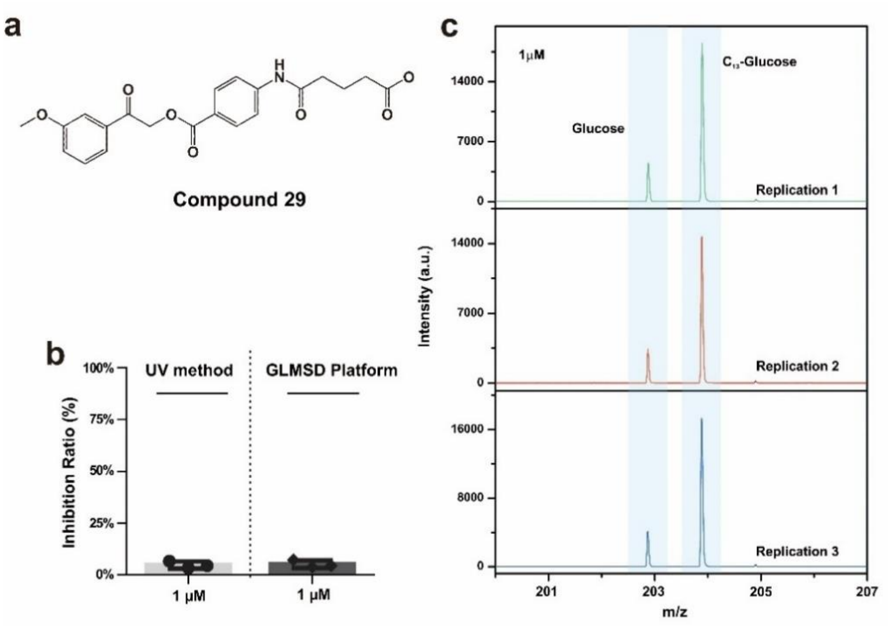

(a) Chemical structures of compound 29. (b) Inhibition profiles of compound 29 against the HK2 enzyme obtained from GLMSD platform. (c) Raw data of HK2 activity in four concentrations of compound 29 detected by GLMSD platform.

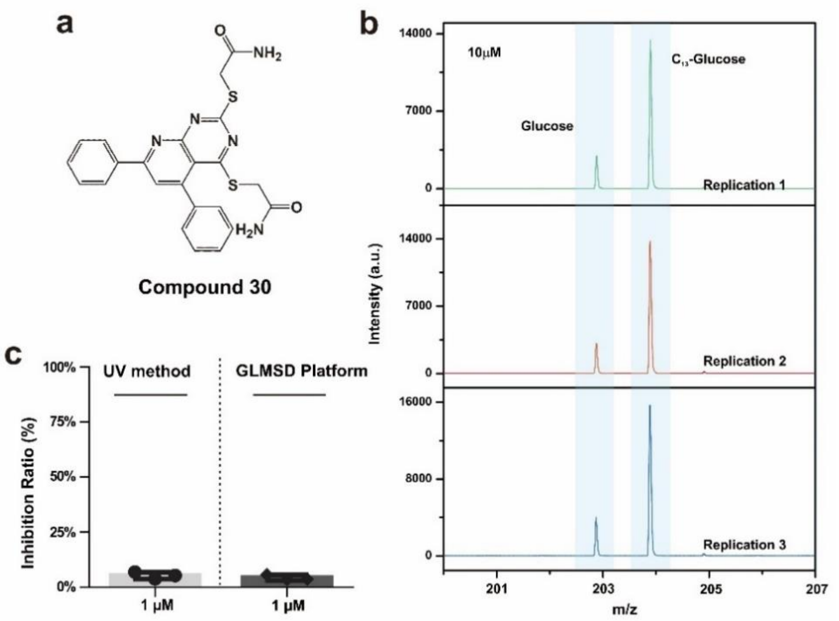

(a) Chemical structures of compound 30. (b) Inhibition profiles of compound 30 against the HK2 enzyme obtained from GLMSD platform. (c) Raw data of HK2 activity in four concentrations of compound 30 detected by GLMSD platform.

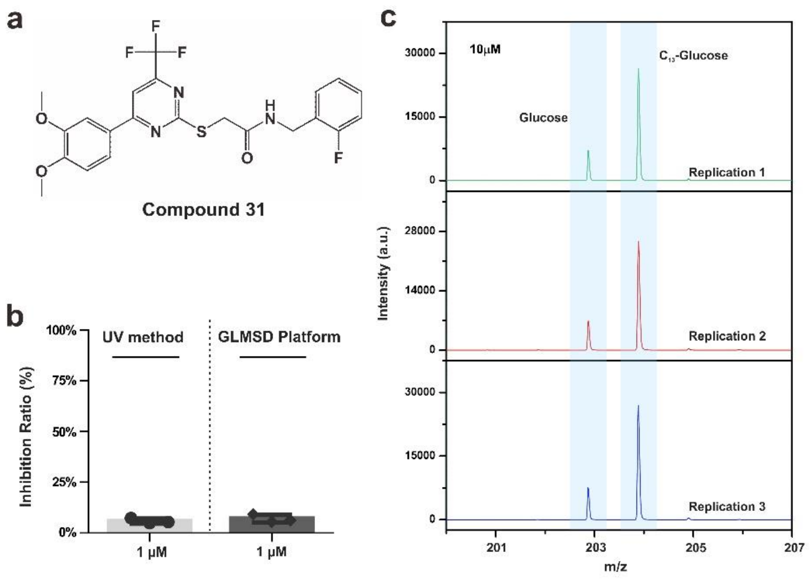

(a) Chemical structures of compound 31. (b) Inhibition profiles of compound 31 against the HK2 enzyme obtained from colorimetric kit and GLMSD platform. (c) Raw data of HK2 activity in four concentrations of compound 31 detected by GLMSD platform.
